# Comprehensive Characterization of the Genetic Landscape of African Swine Fever Virus: Insights into Infection Dynamics, Immunomodulation, Virulence and Genes with Unknown Function

**DOI:** 10.3390/ani14152187

**Published:** 2024-07-26

**Authors:** Dhithya Venkateswaran, Anwesha Prakash, Quynh Anh Nguyen, Muhammad Salman, Roypim Suntisukwattana, Waranya Atthaapa, Angkana Tantituvanont, Hongyao Lin, Tapanut Songkasupa, Dachrit Nilubol

**Affiliations:** 1 Swine Viral Evolution and Vaccine Development Research Unit, Department of Veterinary Microbiology, Faculty of Veterinary Science, Chulalongkorn University, Bangkok 10330, Thailand; 2 Department of Pharmaceutic and Industrial Pharmacies, Faculty of Pharmaceutical Sciences, Chulalongkorn University, Bangkok 10330, Thailand; 3 MSD Animal Health Innovation Pte Ltd., Singapore 718847, Singapore; 4 National Institute of Animal Health, Department of Livestock Development, 50/2 Kasetklang, Phahonyothin 45-15, Chatuchak, Bangkok 10900, Thailand

**Keywords:** African Swine Fever Virus (ASFV), structure, genetic composition, immunomodulation, vaccine targets, virulence genes

## Abstract

**Simple Summary:**

African Swine Fever (ASF) is a viral disease that affects pigs, caused by the African Swine Fever Virus (ASFV). With no available treatment or commercial vaccine, ASF poses a severe threat, with 100% mortality in acute cases. The virus spreads through direct and indirect contact, and current control measures include early detection, isolation, and culling of infected pigs. ASFV has a complex genome with genes and encoded proteins that play a key role in viral replication and survival. This review examines the structure and function of these proteins, highlighting both newly discovered and previously known genes involved in different stages of infection and immune response. It also discusses virulence genes and those with unknown functions, proposing potential future interventions. The findings from this study aim to advance our understanding of ASFV, guiding the development of effective vaccines and control strategies, which are essential for protecting the swine industry and supporting global food security.

**Abstract:**

African Swine Fever (ASF) is a lethal contagious hemorrhagic viral disease affecting the swine population. The causative agent is African Swine Fever Virus (ASFV). There is no treatment or commercial vaccine available at present. This virus poses a significant threat to the global swine industry and economy, with 100% mortality rate in acute cases. ASFV transmission occurs through both direct and indirect contact, with control measures limited to early detection, isolation, and culling of infected pigs. ASFV exhibits a complex genomic structure and encodes for more than 50 structural and 100 non-structural proteins and has 150 to 167 open reading frames (ORFs). While many of the proteins are non-essential for viral replication, they play crucial roles in mediating with the host to ensure longevity and transmission of virus in the host. The dynamic nature of ASFV research necessitates constant updates, with ongoing exploration of various genes and their functions, vaccine development, and other ASF-related domains. This comprehensive review aims to elucidate the structural and functional roles of both newly discovered and previously recorded genes involved in distinct stages of ASFV infection and immunomodulation. Additionally, the review discusses the virulence genes and genes with unknown functions, and proposes future interventions.

## 1. Introduction

African Swine fever (ASF) is a lethal contagious hemorrhagic viral disease caused by African Swine Fever Virus (ASFV) [1]. The disease is currently a threat and has a major impact on the global pig industry and a country’s economy at large, as the disease severely affects the livestock industry, with a mortality rate in acute cases of 100%, including natural death and slaughtering. With a decline in the domestic pigs, pork meat or its products cannot be sold or exported, leading to economic tension. Agriculture is consequently affected, as the feed demand is reduced due to the reduced pig population, and because ASFV can also be transmitted to healthy pigs from the infected ones. The fear of consuming infected meat affects the buyers psychologically. This also impacts revenue from tourism in places where pork is the primary food as buyers might not prefer traveling to these places [2]. 

ASFV strains are classified into 24 genotypes based on the partial nucleotide sequence of the C-terminal end of the B646L gene encoding for the major capsid protein p72 [3,4]. All 24 genotypes of ASFV are prevalent in sub-Saharan Africa. Only genotypes I and II have been reported outside the African continent, including European and Asian countries [5]. ASF was first reported in 1921 in Kenya [6]. Subsequently, the disease spread to several sub-Saharan African countries [7], becoming endemic in the African continent. The ASFV genotype I was introduced into Europe in 1957 and reintroduced after re-entry in 1960 from Portugal. The strain, attributed to ASFV genotype I, later spread into South America and the Caribbean [8]. Fortunately, effective efforts led to the eradication of this genotype from those regions, except for Sardinia, where it persists as an endemic problem. This is unlike the emergence of the ASFV genotype II in Georgia in 2007, which has been prevalent in Europe and Asia since its first emergence [9,10], causing tremendous losses to the global pig industry. The genotype has demonstrated a remarkable capacity for cross-border transmission [11]. Among all 24 prevalent ASFV genotypes, genotype II is responsible for the ongoing pig pandemic in European and Asian countries since its first introduction in Georgia in 2007. China reported the first emergence of ASF in June 2018. Since then, Asian countries have encountered rapidly spreading ASFV outbreaks [12]. Within a brief 9-month period, the virus had reached neighboring Asian countries, threatening the massive >1.2 billion domestic pig population [13,14]. Presently, ASFV outbreaks have been reported in various countries in the Southeast Asia region [15,16,17]. Thailand officially reported the first detection of ASF in 2022 [18]. By the end of 2023, more than 50 countries had been affected by this pathogenic infection [19,20]. The chances of its introduction to newer regions and reintroduction in already evacuated regions are high because of the ease of viral transmission [21].

Presently, the prevention of ASF depends solely on biosecurity. Early detection and culling of infected pigs to restrict the spread of the virus are the only existing control and eradication strategies. Vaccines are commercially available. However, the safety issue is of concern. The first ASFV vaccine, named NAVET-ASFVAC, was commercially available in Vietnam in 2022. It is a live attenuated vaccine (LAV), and the vaccine strain was produced by deleting the *I177L* gene from the highly virulent ASFV isolate Georgia 2007/1 (ASFV-G) [22]. The other commercialized ASF vaccine in Vietnam is AVAC ASF LIVE, based on the ASFV-G-Δ-MGF strain [23]. Though effective, ASFV-G-ΔI177L has a low level of nasal virus shedding, raising concerns for long-term biosecurity prevention, safety, control, and efficacy [24]. LAVs for ASF may not achieve sterilizing immunity, creating room for infection if exposed to wild ASF strains or different genotypes with varying virulence. Although these vaccines have shown safety in experimental settings, there are concerns about potential adverse effects in real-world conditions. Additionally, current evaluations are restricted to weaned piglets with specific age recommendations for vaccination [25]. Early detection, isolation of the infected pigs, and culling in order to restrict the spread of the virus are the existing biosecurity, control, and eradication strategies.

ASFV is a double-stranded DNA virus that encodes for more than 150 structural and host-induced immunoregulatory proteins [21]. ASFV belongs to the genus *Asfivirus*, the only member of the family *Asfaviridae*, and to the phylum of Nucleocytoviricota, classifying ASFV as one of the nucleocytoplasmic large DNA viruses (NCLDV) [26]. Both the intra- and extra-cellular forms of the virus are infectious [27], with monocytes and macrophages as the primary target. Domestic pigs and wild boars of all ages are susceptible, with a mortality rate approaching 100 percent [28,29]. ASFV is transmitted through contact with infected animals and materials. The soft ticks of the genus *Ornithodoros* are vectors known to transmit ASFV from warthogs to domestic pigs.

Due to its classification as a DNA virus, ASFV is genetically rather conserved. However, recombination between different genotypes has been evident. Genetic recombination plays a significant role in the evolution of African Swine Fever Virus (ASFV) genomes, as evidenced by recombination events identified in ASFV strains from East Africa, where diverse virus transmission cycles occur. Phylogenetic reconstructions have indicated ASFV recombination events in MGF, *E183 L*, *B602 L*, *EP153R*, and *EP402R* (CD2v) genes [30]. ASFV serotype-specific proteins, such as EP153R and CD2v, frequently undergo recombination, particularly in regions with multiple transmission cycles. These recombination breakpoints are often found in immunologically important proteins, which highlights their role in driving virus evolution. The adaptive evolution signature of EP153R and CD2v, with a pN/pS ratio greater than 1, suggests substantial selective pressure on these proteins, supporting the presence of breakpoints and recombination events [31]. Furthermore, the CD2v (EP402R) protein sequence from the Georgia 2007/1 sequence clusters more closely with the Malawi Lil20/1 and Kenya 1950 isolates, whereas the EP153R protein sequence from the Georgia 2007/1 isolate clusters more closely with the warthog isolate. This suggests that past recombination events may have occurred, although there are no longer contiguous genome segments where recombination between genomes is evident [26].

Recent evidence of ASFV genetic recombination was additionally reported in China. Recombinant ASFVs with mosaic genomes of genotype I and II viruses were detected in pigs in China. These recombinants were genetically similar to low-virulence NH/P68-like genotype I, but contained genetic material from high-virulence Georgia07-like genotype II; specifically, 10 discrete fragments making up over 56% of their genomes had evolved as a new branch in the phylogenetic tree. Animal studies with one of the recombinant viruses demonstrated high lethality and transmissibility in pigs. Deletion of the virulence-related genes MGF_505/360 and EP402R, derived from virulent genotype II virus, significantly reduced its virulence. The live attenuated vaccine derived from genotype II ASFV was not protective against the challenge of the recombinant virus, indicating the potential threat posed by these naturally occurring recombinants to the global pig industry [32].

Although several review studies are available on ASFV, a constant upgrade is necessary as this is a very active field of research, with various genes and their functions being explored at a rapid pace. Therefore, this review presents and discusses in detail the structural features, genetic composition, and functional role of newly recorded genes of ASFV involved in various stages of ASFV infection, in addition to the existing genes. The review brief discusses the mechanism of action of potential virulence genes. The latter section of this review discusses the immunomodulatory mechanism of ASFV and sheds light on the genes with unknown functions.

## 2. Structure

**Figure 1:**
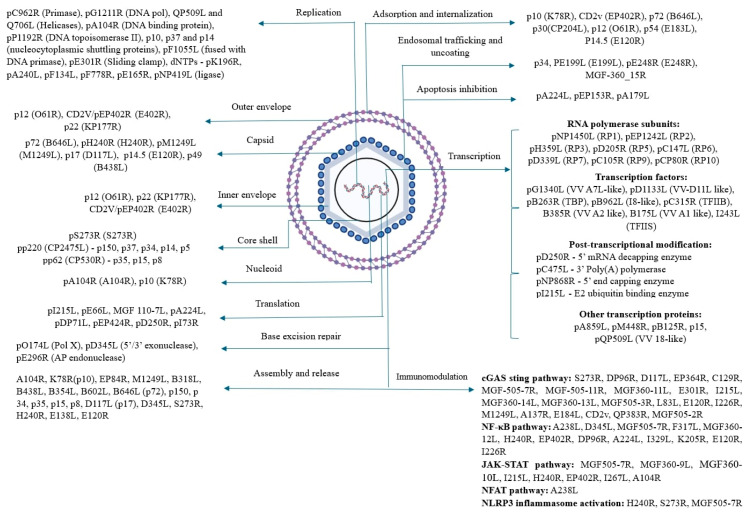
Genes and encoded proteins of ASFV categorized based on the structural layers, role in the infection cycle, immunomodulation, apoptosis inhibition, and virulence. The genes whose functions are unknown is also described. VV—Vaccinia virus; RP—RNA polymerase, DNA pol—DNA polymerase; dNTPs—deoxynucleotide triphosphate; TFIIB—Transcription factor IIB; TFIIS—Transcription elongation factor.

ASFV is a large double-stranded enveloped DNA virus with icosahedral symmetry. Its structure comprises five different layers, namely an outer envelope, capsid, inner envelope, core shell, and nucleoid. Each layer consists of different genes and proteins spanning the different layers (Figure 1) [1]. The ASFV genome ranges from 170 to 194 kilobases (kb) in length, containing 150 to 167 ORFs. The variation in the number of genes depends on the strain of the virus [33]. Within this genome, 150 to 200 proteins are encoded, consisting of 68 structural proteins and over 100 non-structural proteins (Supplementary Table S1) [34]. While many of these proteins are not essential for viral replication, they play significant roles in host interaction to facilitate its survival and transmission [35].

### 2.1. Outer Membrane/Envelope

The outer membrane or envelope is the outermost layer of ASFV. The existence of the external envelope membrane protects the virus and retains the infectivity of the virus. 

Presently, at least three different proteins have been reported in association with the outer membrane. The three proteins are p12, CD2V (pE402R), and p22 which are encoded by *O61R*, *EP402R*, and *KP177R* genes, respectively [1,35]. Firstly, p12, encoded by the *O61R* gene, is a conserved viral attachment protein, synthesized during the late phase of the infection cycle. It functions in promoting viral adsorption by facilitating the host cellular receptor recognition, based on specific polypeptide binding [36]. Secondly, CD2V, encoded by the *EP402R* gene, is a type I transmembrane protein. This CD2V protein is considered a marker molecule, as it is detectable on the extracellular surface of the ASFV [37,38]. The protein is named CD2V as its structure is similar to the host adhesion protein CD2, expressed on T cells and NK cells [38]. CD2v is a structural transmembrane glycoprotein. It is crucial for hemadsorption. CD2V is expressed on the surface of infected cells. This CD2v interacts with sialic acid residues on the surface of RBCs, allowing infected cells to bind to the RBCs [39]. Thirdly, the protein p22, encoded by *KP177R*, is a viral structural transmembrane protein transcribed during early stage of infection. p22 is a conserved protein located on the ASFV inner membrane. Upon infection, it is observed on the surface of infected host cells. The function of p22 is unknown [40].

### 2.2. Capsid

Capsid is the protein shell that encloses the core shell and nucleoid of the virus. This protein shell has a diameter of approximately 250 nm, harboring 2760 pseudo-hexameric capsomers and 12 pentameric capsomers [1]. The viral capsid structure comprises 8280 copies of p72, the major capsid protein (MCP), 60 copies of the penton protein pH240R, and a minimum of 8340 copies of stabilizing minor capsid proteins, including pM1249L, p17, p14.5, and p49. The capsid is icosahedral in shape, and contains the major capsid protein and penton protein located in the outer capsid shell. The major capsid protein (p72) is located on the surface of the capsid. The penton protein (pH240R) is concentrated at the vertex. Below the outer capsid shell is the network formed by the minor capsid proteins [41]. Five penton proteins can construct a pentameric capsome [42]. At present, the known capsid proteins include p72, pH240R, pM1249L, p17, p14.5, and p49 encoded by the genes *B646L*, *H240R*, *M1249L*, *D117L*, *E120R*, and *B438L*, respectively.

p72, encoded by *B646L*, is a protein highly expressed during the late stage of infection. The trimeric p72 protein consists of two jelly-roll-folded domains that interact with the inner lipid membrane. This interaction results in the formation of pseudo-hexameric capsomers [43]. p72 accounts for about one-third of the total mass of the virion (~31–33%). Hence, it is among the major antigens detected in infected pigs [41]. The protein p72 is highly conserved, underscoring its antigenic stability and robust immunogenicity. These two properties render p72 a pivotal antigen for serological diagnosis [44]. p72 is involved in morphogenesis and viral entry [45].

pH240R, encoded by *H240R*, is a pentameric structural protein [42]. It is confined in a p72 shell in the cytoplasm with a jelly roll and a globular cap homologous to p72. This cap forms an apex, crucial for the capsid structure [42]. pH240R is primarily involved in the formation of viral assembly. The pH240R has critical roles in inducing ASF virulence and pathogenicity through NF-κB and JAK-STAT pathways. It also mediates infection-induced inflammatory responses through NLRP3 inflammasome activation [46,47].

pM1249L, encoded by *M1249L*, is a minor capsid protein with 1249 amino acids (aas). pM1249L has main functions in immunomodulation. Firstly, it functions in inhibiting IFN-β promoter activity and transcriptional activity. Additionally, the protein degrades IRF3 through lysosomal pathways and suppresses TBK1 phosphorylation, thereby inhibiting the cGAS-STING pathway [48]. p17, encoded by *D117L*, is positioned at the 140–150 kb central region and close to the right variable region of the genome. The protein forms trimers and is located between three consecutive pseudo-hexameric capsomers at the interface of the central gap region. p17 binds tightly to the base domain of p72, encircling each p72 capsomer within the inner capsid shell. This tight binding thereby firmly anchors to the inner membrane, which is vital for the viability and stability of the virus. p17 plays a key role in capsid assembly and maturation and inhibits the cGAS-STING pathway [49,50]. p14.5, encoded by *E120R*, interacts with p72. The interaction between these two proteins facilitates the release and transport of mature ASFV virions from the virus factory (VF) to the plasma membrane of the host cells. Upon reaching the host plasma membrane, the ASFV virions are released from the host cell to infect the host system. p14.5 needs to bind to p72 to perform the release and transport functions. Therefore, p14.5 could potentially serve as another marker for the ASFV capsid [51]. p49, encoded by *B438L*, is a trans-prenyltransferase. It has a key role in morphogenesis for the vertex formation of the icosahedral capsid [26,52].

### 2.3. Inner Membrane/Envelope

The inner envelope is a double-membrane lipid domain that encompasses the icosahedral capsid. The proper assembly of the core shell relies on the formation of both the outer capsid and the inner membrane [42]. The inner membrane consists of p54, pE248R, pH108R, and pE199L which are encoded by the genes *E183L*, *E248R*, *H108R*, and *E199L*, respectively.

p54, encoded by *E183L*, is a type II structural protein [53]. This protein facilitates the entry of the virus to the perinuclear area of the cells by binding to the LC8 chain of motor protein dynein to form the p54–dynein complex. The proteins p54 and p30 have a synergistic role in binding ASFV to the target host cells. This p54–dynein complex induces caspase-3 activation and apoptosis. In the late stage of the ASFV infection, it is essential for the recruitment of envelope precursor fragments. Both p30 and p54 are commonly used as diagnostic antigens for detecting antibodies against African Swine Fever Virus (ASFV). However, p30 is typically preferred due to its higher specificity and sensitivity compared to p54 [53,54]. pE248R, encoded by *E248R*, is a late structural protein. It is a myristoylated integral membrane protein. pE248R is an important component of the redox pathway required for disulfide bond formation. Although dispensable for virus assembly, pE248R plays a crucial role in virus-cell fusion and early virus infectivity post-entry. Additionally, pE248R is necessary for the viral core transfer to the cytoplasm [55,56]. pH108R, encoded by *H108R*, is a transmembrane structural protein present intracellularly in the viral factories of swine. It plays a key role in ASF virulence [57]. pE199L, encoded by *E199L*, is a cysteine-rich integral transmembrane structural polypeptide with cytosolic intramolecular disulfide bonds. This protein is required for membrane fusion, viral uncoating, and viral core entry [58].

### 2.4. Core Shell

The core shell of ASFV is a 30 nm dense layer of protein, accounting for around one-third of the mass of ASFV. The core shell protein accounts for approximately 25% of the total protein content of ASFV structural proteins [59]. The core shell protein carries pS273R encoded by *S273R* genes and mature proteins derived from two precursor polyproteins, pp220 and pp62, which are encoded by the largest two genes, *CP2475L* and *CP530R*, respectively [60]. The polyprotein products of pp220 include p150, p37, p34, p14, and p5, and those of pp62 include p15, p35, and p8.

Firstly, pS273R, encoded by the *S273R* gene, is a cysteine protease that is a late protein expressed during the late stage of infection. It belongs to the SUMO-1-specific protease family. This protein is found to exist in cytoplasmic viral factories. The pS273R structure comprises an N-terminal arm domain and the core domain. The former spans its amino acid residues from M1 to N83. This is a distinctive feature exclusive to ASFV. This domain plays a critical function in preserving the enzymatic activity of pS273R. On the other hand, the “core domain” encompasses amino acid residues from N84 to A273 [61]. pS273R plays a key role in facilitating the maturation and infectivity of the ASFV particle [62]. In mature virions, polyproteins pp220 and pp62 are proteolytically processed by the cysteine ubiquitin-protease pS273R [63]. This proteolysis of pp220 and pp62 is p72-expression-dependent. pp220 expression forms the base for pp62 processing. ASFV virions may present without a core or lose their infectivity if the processing of pp220 and pp62 is inhibited [36]. pp220 is cleaved to yield p150, p37, p34, p14, and p5, while pp62 yields p15, p35, and p8 [64].

Secondly, pp220 yields five proteins, p150, p37, p34, p14, and p5. Each of the protein products have different functions. p150 is a highly immunogenic structural protein. It is the second abundant virion protein by mass after p72, with amino acid residues 894 to 2476. It helps in viral assembly by forming matrix structures [44]. p37 is a structural protein which is initially distributed throughout the infected host cells. At early stages of infection, it becomes concentrated in specific nuclear regions of the cells and later detected in the cytoplasm of infected cells at the late stage of infection [65]. In the early infection phase, p37 helps in viral entry and accumulation in the cytoplasm. It is the first nucleocytoplasmic shuttling protein encoded by ASFV that facilitates transport between the nucleus and cytoplasm. Remarkably, CRM1 exportin is not needed for nucleocytoplasmic shuttling function of p37 [1]. Additionally, it is essential for replication [36]. The p34 protein is the key core shell protein of ASFV. The protein is highly conserved and immunogenic due to its ability to react with swine convalescent sera. Additionally, p34 possesses a detectable T-cell epitope on its surface, suggesting its capacity to induce a cell-mediated immune response. Previous research suggests that mutations in p34 can be attributed to replication and assembly of the ASF virion [66,67]. p14 is a structural protein. It plays a role in the replication of ASFV by facilitating nuclear transport activity [1,59]. p5 is a structural protein. It contains a distinctive tryptic peptide that spans 43% of its amino acid sequence [35].

Thirdly, pp62 yields three proteins, p15, p35, and p8. Each of the proteins has a different function. p15 is a matrix protein. It spans the nucleoid and the inner envelope. p15 is involved in viral assembly. It may be involved in viral transcription. It is highly possible that p15 interacts with other components during the virus assembly process and facilitates the stabilization of the mature virus particle [68]. It binds with dsDNA and is involved in viral genome packaging. The functions of p15 in terms of the viral infection cycle need further study [69]. p35 is characterized by a tightly packed structure comprising eleven α-helices and four 310-helices. p35 acts as a docking scaffold. It assists in drawing the host membrane and other components to the core shell during the assembly of the virus, thus stabilizing the mature virion [60]. p8 is a 67-amino-acid mature structural protein with low immunogenicity and rapid degradation [35].

### 2.5. Inner Core/Nucleoid

The nucleoid or inner core is the protected innermost layer of this five-layered virus. This is the central region of the virus particle where the genetic material is located. The ends of the viral genome are characterized by covalently cross-linked hairpin loops. This region is essential for the replication of the virion as it contains the genetic blueprint essential for the virus to control the host cell machinery and reproduce [1]. The nucleoid consists of two proteins, pA104R and p10, encoded by the *A104R* gene and *K78R* gene, respectively.

Firstly, pA104R is a type II DNA-binding protein expressed during the late stage of infection. Functionally, it is similar to bacterial HU (histone-like protein from *E. coli* strain U93) and Integration Host Factor (IHF). pA104R exhibits ATP-independent binding to single- or double-stranded DNA [70]. Binding with topoisomerase II, pA104R induces DNA supercoiling [71]. The viral infection is disrupted by interfering with this process. This disruption yields viral particles lacking DNA or with abnormal DNA structures that can still elicit an immune response for host immunization [72]. The proteins are also involved in genome packaging as a part of viral assembly [73]. pA104R is also involved in inhibition of JAK-STAT pathway by attenuating STAT1 phosphorylation, which results in epigenetic modifications contributing to pA104R pathogenicity. This identifies a novel role for pA104R in ASFV evasion of host innate immunity [74]. Secondly, p10 is a structural DNA-binding protein with similar affinity for both dsDNA and ssDNA [44]. This binding affinity aids in viral replication [75]. It is also involved in viral entry and DNA packaging [76].

## 3. The Stages of the ASFV Infection

The stages of ASFV infection of host macrophages are classified as early, intermediate, and late based on the time of occurrence. The early stage of ASFV infection occurs between 0 min post-infection (mpi) and 8 h post-infection (hpi). The intermediate stage of ASFV infection occurs in the range of 8–16 hpi. The late stage of ASFV infection occurs at 16–24 hpi.

The ASFV genes are classified as early, intermediate, and late genes based on stage of viral infection cycle when they are transcribed. Early genes are expressed early in the viral infection cycle and encode proteins involved in regulating viral gene expression, modifying host cell functions, and initiating viral DNA replication. Intermediate genes are expressed after early genes’ encoding proteins involved in various aspects of viral replication, such as DNA replication, transcription, and protein processing. Intermediate gene products facilitate the production of viral genomes and structural components necessary for assembling new virions. Late genes are expressed later in the viral replication cycle, typically after viral DNA replication has occurred. They encode structural proteins that form the viral capsid, along with proteins crucial for viral assembly and egress. These gene products play a vital role in packaging viral genomes into newly formed virions and aiding their release from infected cells.

Within 30 min of infection, the virion undergoes internalization either by endocytosis or the macropinocytosis pathway, subsequently entering early endosomes. Within 30 to 90 mpi, the virions are transported to the late endosomes, followed by uncoating and release. Between 4 and 6 h post-infection (hpi), early genes are expressed. These early genes, including genes that play crucial roles in replication, late transcription-related protein, and MGF-related proteins with host-immune evasion, are expressed at 4–6 hpi. The replication of the ASFV genome takes place within 6–8 hpi. ASFV belongs to the phylum Nucleocytoviricota, which includes nucleocytoplasmic large DNA viruses (NCLDVs). NCLDVs can replicate in both the nucleus and cytoplasm. Thus, replication of the ASFV genome occurs in both the nucleus and cytoplasm of the host cells. However, a major portion of replication takes place in the cytoplasm [58]. At 8–16 hpi, the intermediate genes and late genes are synthesized. These genes encode for viral structure-related proteins responsible for budding virus formation and transcription factor packaging. Genes expressed early in infection exhibit higher levels of expression compared to genes expressed later in the infection process. The late stage of ASFV infection involves assembly of the virus factory (VF). After 24 h, there is viral shedding from the cell membrane to outside the cell [1].

### 3.1. Adsorption and Penetration/Internalisation

The adsorption and internalization of ASFV into the host cells is facilitated primarily through dynamin-dependent clathrin-mediated endocytosis (CME). In the case of a lack of a host cell receptor at the binding site, ASFV facilitates receptor-independent internalization through macropinocytosis. CME and macropinocytosis require actin rearrangement to perform virion internalization [77]. The internalization of ASFV by CME and macropinocytosis can occur simultaneously. Thus, these two internalization processes are not mutually exclusive [73]. In general, the internalization of viruses into the host cell via CME usually takes place in small to medium-sized viruses (<150 nm). Larger viruses (>150 nm) internalize through macropinocytosis or phagocytosis. CME and macropinocytosis are not mutually exclusive; instead, they cooperate with each other [73].

There is also a recorded interaction between the mentioned endocytosis pathways, wherein the viral entry through actin reorganization and the ruffling that occurs in micropinocytosis can trigger and aid the CME process [78].

#### 3.1.1. Cellular Receptors Involved in Adsorption and Internalization

Presently, no specific receptors or attachment proteins for ASFV identified in the adsorption process have been reported. However, it has been identified that some host cellular factors act as receptors for binding to the virus and facilitating viral entry. The cellular factors of macrophages, including CD163, dynamin, clathrin, EGFR, PI3K, actin, sodium/proton exchanger (Na/H), EGFR, PKC, phosphorylated PI3K, Pak1, tyrosine kinase, microtubules, Rab7, CD45, CD203a, MHC II, and activation of Rho-GTPase Rac1 [79], induce virion adsorption and internalization.

The cysteine-rich scavenger receptor CD163, expressed only in macrophages and monocytes, is a receptor of ASFV involved in macrophage maturation. However, its sole involvement in ASF pathogenesis is questionable, as previous experiments with CD163-knockout pigs have demonstrated that these pigs can still be infected by ASFV [80]. 

CD45 is strongly expressed in adherent porcine bone marrow cells upon ASFV infection. Therefore, CD45, along with MHCII, is a key receptor for macrophage invasion by ASFV [81]. CD203a, a cell surface marker, has been linked to the vulnerability of myeloid cells to ASFV. The role of CD203a in ASFV entry may be associated with the maturity and differentiation of porcine blood monocytes into macrophages in vitro [82]. During this process, CD163 and CD203a are upregulated, contributing to increased ASFV infection. Interestingly, while the expression of CD203a rises during monocyte–macrophage differentiation, its levels do not necessarily correlate with the extent of ASFV infection. These observations underscore the potential involvement of CD203a in ASFV entry. Further investigations are required to understand the specific mechanisms of CD203a and its significance in the infection process [82].

Dynamin and clathrin are involved in the scission process, and the assembly of clathrin-coated pits in the clathrin-mediated endocytosis (CME) pathway, respectively [83]. Rab7 is involved in maturation of CCV from early to late endosomes [80]. EGFR, sodium/proton exchanger (Na/H), PKC, phosphorylated PI3K, Pak1, Rac1, and tyrosine kinase are involved in various stages of the actin-mediated micropinocytosis pathway [84].

Microtubule is a cytoskeletal structure that plays a crucial role in the entry of ASFV. Upon reaching the replication site near the microtubule organizing center (MTOC), incoming ASFV virions rely on the microtubule network for efficient trafficking. Endosomal maturation, which is essential for successful viral entry, requires the movement of endosomes towards the perinuclear area via microtubules. Disruption of microtubules using depolymerizing agents such as nocodazole hampers ASFV trafficking, emphasizing the importance of microtubules in the viral entry. Additionally, the activation of Rac1, which facilitates microtubule stabilization, is crucial during the early stages of ASFV infection. These findings emphasize the complex interplay between ASFV and the microtubule cytoskeleton for effective viral entry and subsequent infection [78].

#### 3.1.2. Viral Internalization through Clathrin-Mediated Endocytosis

Clathrin-mediated endocytosis (CME) is a well-studied viral entry mechanism characterized by the formation of clathrin-coated pits (CCPs) that consequently transform into coated vesicles in the cytoplasm to facilitate the receptor-mediated internalization of the virus [80].

In the presence of a cholesterol flux, ASFV internalizes the host. CME begins with specification of the assembly site, followed by CCP assembly by the hetrotetrameric adaptor protein-2 (AP2) complex (α adaptins, β2 adaptins, μ2 subunit, σ2 subunit). Two AP-2 complexes are required for the CCP assembly. Amphiphysin-recruited dynamin regulates the initiation, stabilization, and maturation of clathrin-coated pits (CCPs) and the scission process. During the scission process, dynamin relocates from the clathrin-coated pits (CCPs) to the neck of invaginated CCPs, where it forms helical rings. These rings catalyze membrane fusion between the inner envelope and the late endosomal membrane, ultimately releasing the genome-containing core into the cytoplasm [46,85]. The invagination is facilitated by dynamin, endophilin, and actin, after which the clathrin-coated vesicles lose their coating to form early endosomes [1].

#### 3.1.3. Viral Internalization through Macropinocytosis

Besides viral entry through CME, ASFV can enter the host cells through macropinocytosis. This is a non-selective, non-receptor-mediated process. This process involves intracellular signaling and the formation of actin-dependent folds on the plasma membrane, known as ruffles or blebs. These structures facilitate the formation of a vesicle called a macropinosome, which is invaginated into the cytoplasm [24]. This is a collaborative mechanism that requires Na+/H+ exchanger, EGFR activation, PI3K, Pak1 kinase activation, and small Rho-GTPase Rac1 activation [86].

In the case of ASFV, the virus directly activates receptor tyrosine kinase (RTK), which in turn triggers Ras, a type of small GTPase. This activation initiates three signaling pathways involving phosphoinositide-3-kinase (PI3K), Rac-1, Rho-GTPase (Rac and/or Cdc42), Rab5, and Arf6. These pathways induce actin and microfilament/microtubule rearrangement, resulting in membrane ruffling and closure. Rac-1 can generate three types of membrane ruffles, while Rab5 relocates to circular ruffles alongside its effector, RN-tre. This effector interacts with F-actin, facilitating ruffle-associated actin cross-linking. Additionally, Rabankyrin-5, another Rab5 effector, binds to newly formed macropinosomes, potentially guiding Rab5 to these structures. Moreover, Rab5, along with effectors Rabankyrin, PAK1, and CtBP1, participates in ruffle closure, assisted by the contractile activity of myosin. Arf6 collaborates with PAK1 and its effectors to induce membrane curvature. This collaboration is involved in various processes, including endocytic membrane trafficking, plasma membrane remodeling, and intracellular macropinosome trafficking. The functions of effectors activated by Ras and Arf6 often overlap, suggesting synergistic roles in Ras-mediated macropinocytosis. Furthermore, Pak1, along with its effector CtBP-1/BARS, actively participates in all stages of macropinocytosis. PI3-kinase (PI3K) is an essential molecule that directs endosomal traffic and maturation. The formed macropinosomes are functionally equivalent to early endosomes (EEs) within the cell [87,88].

#### 3.1.4. Proteins Involved in Viral Entry and Internalization

**Figure 2:**
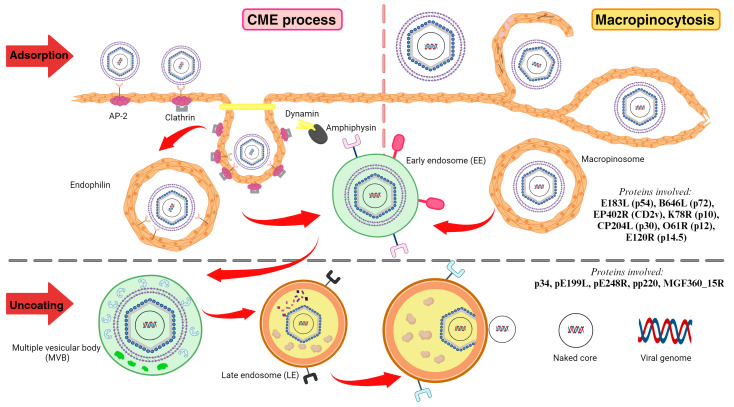
The proteins encoded by the ASFV genes that are involved in adsorption and uncoating. AP-2—adaptor protein-2.

The proteins involved in viral entry and internalization include p54, p72, CD2V, p10, p30, p12, and p14.5 encoded by the genes *E183L*, *B646L*, *EP402R*, *K78R*, *CP204L*, *O61R*, and *E120R*, respectively (Figure 2).

p54 is a structural protein that facilitates adsorption through direct links with LC8, a cytoplasmic 8-kDa light chain of the microtubule motor protein dynein that mediates microtubule-mediated transport to defined subcellular sites of action [78]. This interaction happens in vitro and in vivo, where proteins colocalize at MTOC during infection [89]. Antibodies against p54 protein inhibited the first step of the viral infection cycle (bending), proving their role in viral attachment [36]. Though structurally distinct, the structural protein p30 expressed in early stages of infection performs the same function as p54, which is expressed in the late stages of ASFV infection, especially in viral entry. Despite being categorized as an early antigenic membrane protein and secretory protein, its exact location, whether inner or outer envelope, and role in viral entry, are still unknown [90]. Like p54, antibodies against p30 have also been proven to neutralize the virus by restricting viral internalization [91]. p72 is involved in viral internalization, which is facilitated through viral bending [80]. Studies showed that p72 does this by binding with host membrane protein (factor) CD1d, which recruits EPS15 to form a complex and carry out CME process [92]. The ASFV CD2V facilitates binding of host RBCs to infected macrophages and extracellular viral particles. By interaction with actin-binding adaptor protein SH3P7 and adaptor protein AP1, it involves cell–cell adhesion and endocytosis [93]. p10 is also involved in viral attachment and adsorption [36]. p12 is involved in viral attachment and invasion. It helps in locating the host cell receptor to initiate attachment [66,94]. p14.5 is involved in assembly and mediates intracellular virus transport [72].

### 3.2. Endosomal Trafficking/Uncoating

Endosomal trafficking is a low-pH-dependent process wherein the pH drops from 6.5 to around 5 (membrane acidification) as the virion travels through the endosomal system. After internalization, the early endosomes (macropinosomes) with a pH of 6.5 mature into multivesicular bodies (MVBs) with a pH of 6. At a pH of 5, decapsidation/uncoating of the outer envelope and capsid of virions in the MVBs occurs, resulting in formation of late endosomes carrying uncoated virions [95].

This endosomal transport of virions is characterized by a change in the endosomal markers. The Rab GTPase protein family is the key regulator of the maturation of endosomes. At early stages of infection (5–30 mpi), the markers EEA1 and Rab5 are detected along with the capsid and inner envelope. The decapsidation occurs at 30–45 mpi, and the MVBs are recorded by the presence of MVB marker CD63. The later period of infection (30–90 mpi) is marked by the presence of cellular factor (Rab7) and lysosome markers (Lamp1 and cathepsin L+), along with the exposed inner envelope and core shell proteins, p17 and p150. Rab7 serves as an important regulator of the maturation of early to late endosome [80].

The proteins involved in endosomal trafficking/uncoating include p34, pE199L, pE248R, pp220, and MGF360_15R (Figure 2). pE199L is involved in membrane fusion and core delivery. However, this protein is not involved in assembly. Cells infected with ASFV and containing functional pE199L display porous structures, indicative of core penetration. This was corroborated by the detection of expressed proteins p12 and p150 [58]. The porous structures were 12 times reduced in ASFV infected cells lacking functional pE199L. pE248R protein has sequence similarity with the entry/fusion complex protein L1 of vaccinia virus (VACV). Through experimental study, it was identified that the indicative cytosolic cores were more evident in recombinant viruses carrying functional pE248R. This suggests that this protein is a component of the putative EFC of ASFV situated in the inner membrane [96]. Through the endosomal pathway, there is a gradual weaking of attachment of the core from the inner membrane. pp220 is crucial for making maturation-related structural changes necessary for this core detachment from the inner membrane, resulting in core penetration into the cytoplasm during ASFV infection. The N-myristoylation of pp220, which triggers the required proteolytic processing of pp220 by p237R, is essential for core detachment [97].

The entry-fusion complex of ASFV comprising *E199L*, p34, *MGF360*-15R, and *E248R* was reported recently [98]. There is a structural and mechanism-oriented conservation of entry-fusion proteins within the poxvirus family and, on a larger scale, the NCLDV family. The ASFV proteins pE199L and pE248R have similarities with the entry-fusion complex (EFC) proteins of VACV, a member of the poxvirus family [99,100]. Positive interactions between pE199L and various ASFV proteins indicated the formation of a novel EFC of ASFV. The scrutinization of proteins based on interactions between ASFV protein complex members and cellular proteins, particularly related to Rho GTPases signaling, suggests common functions and cellular targets, shared by proteins *E199L*, p34, *MGF360-15R*, and *E248R*, making p34 (*CP2475L*) and *MGF360-15R* new members of ASFV entry-fusion proteins [98].

### 3.3. Biosynthesis

The exposed inner envelope merges with the inner membrane of the late endosomal membrane, facilitating the release of the free viral core into the cytoplasm. Immediately after release, viral factories are formed. These are replication sites in the perinuclear space of the host cell that contain viral replication organelles and other components required for replication, assembly, and maturation of new ASFV particles. Transport to this perinuclear space from the endosome is facilitated by the activation of the microtubule system of the perinuclear microtubules [51]. p54 facilitates virus entry to the perinuclear area. Host cell phosphatidylinositol-3-phosphate (PtdIns3P) and biphosphate PtdIns (4,6) diphosphorus play crucial roles in the progression of early infection events leading to the initiation of ASFV replication [101].

#### 3.3.1. Transcription

**Figure 3:**
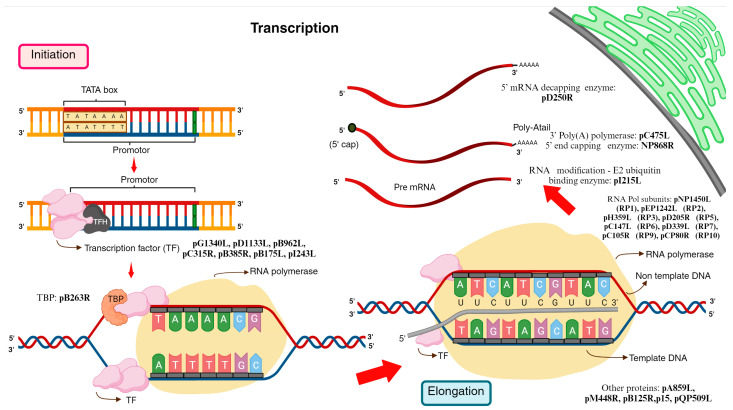
The proteins encoded by ASFV genes that are involved in transcription.

The transcription machinery exhibits consistency across various systems. Previous studies have conclusively shown that the host-independent transcription machinery and components of ASFV, belonging to the NCLDV, resemble those of vaccinia virus (VACV) and the eukaryotic Pol II transcription system. This host independency, resulting from transcription self-sufficiency, enables ASFV proliferation in swine and soft-tick vectors, despite their evolutionary distance. The transcription machinery components encoded by ASFV that are homologous to the eukaryotic Pol II transcription system include general transcription initiation factors such as TFIIB (C315R) and TBP (B263R), along with transcript cleavage/elongation factor TFIIS (I243L) (Figure 3, Table 1). Furthermore, ASFV encodes virus-specific transcription initiation factors that are non-homologous to eukaryotes, directing transcription from distinct viral promoters [102].

Transcription start sites (TSSs) for 151 out of 153 ASFV-BA71V genes have been identified [103]. The initiator element (Inr), a short motif overlapping the TSS, exhibits a distinct bias: TA* for early promoters and TA*TA for late promoters, (*—TSS). The bias in the Inr sequence likely results from direct interactions with the template ssDNA positioned in the RNAP active site. A conserved region was identified upstream of the transcription start site (TSS). This region corresponds to early-promoter motifs (EPMs) and late-promoter motifs (LPMs). The ASFV early-promoter motif (EPM) showed higher conservation compared to the late-promoter motif (LPM) due to some overlap between the sequences of late- and early-gene promoters. The EPM resembled the upstream control element (UCE) of early vaccinia virus (VACV) genes, suggesting a possible interaction with the conserved D6/D7 heterodimeric transcription initiation factor in ASFV which could induce early-promoter transcription. Additionally, the virus-encoded TBP and TFIIB homologs, which are expressed earlier during infection, may be recruited to the late-promoter motif (LPM) to facilitate late or post-replicative transcription. In both the Pol II system and archaea, these factors typically bind to the TATA-box and B-recognition elements upstream of the transcription start site (TSS), resulting in the recruitment of RNA polymerase to form the preinitiation complex (PIC). Given that ASFV encodes TBP and TFIIB homologs, it can be inferred that ASFV-RNAP forms a similar PIC. Despite the T/A-rich nature and upstream location of the ASFV LPM, it did not exhibit significant similarity to the TATA-box consensus. TSS analyses revealed that ASFV can enhance protein diversity and repertoire through the utilization of alternative (intragenic) TSSs, leading to the production of 5′ shortened mRNAs encoding N-terminally truncated proteins [102,103].

It is suggested that a shared evolutionary history exists between viruses of the NCLDV family and Killer plasmids. Some yeast strains harbor “Killer plasmids” encoding a secreted toxin, offering a competitive advantage in resource competition. These plasmid-borne genes are transcribed by a plasmid-encoded ultra-minimal RNA polymerase system related to NCLDVs. Notably, the plasmid promoter motifs show a high degree of similarity to those of the VACV upstream control element (UCE) and the ASFV early-promoter motif (EPM). The UCE-binding D6/D7 early transcription factor, which is highly conserved among NCLDVs, is also partially present in Killer plasmids. This factor, which possesses ATPase activity, likely plays a crucial role in early infection transcription, enabling the plasmid to function independently of the host cell machinery. By employing a third next-generation sequencing (NGS) method for understanding ASFV transcription termination, termination sites were identified for over two-thirds of ASFV genes, showing a ∼6–7 residue polyT (polyU in mRNA) 3′ signature in both early and late genes. This signature is similarly present in archaea and eukaryotic Pol III but with different bacterial terminators. Approximately one-third of the genes of ASFV lacked clear termination motifs, which may indicate a factor-dependent termination mechanism. This mechanism could involve conserved VACV-like RNA helicases that facilitate mRNA release and termination [101]. Following the completion of transcription, the pre-RNA undergoes 5′-capping and 3′-polyadenylation in the cytoplasm. The PolyA tail is added to the 3′ end of the transcript with the help of a polyadenylation enzyme (C475L) encoded by ASFV. 5′triphosphatase (TPase), guanylate transferase (Gtase), and methyltransferase (Mtase) pNP868R are the three enzymes involved in 5′-capping [103].

#### 3.3.2. Translation

**Figure 4:**
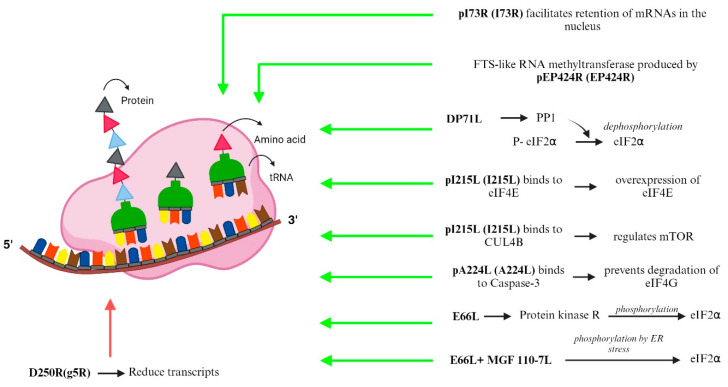
The ASFV genes and proteins that are involved in translation. eIF—elongation initiation factor. The green color arrows depicts the ASFV proteins that enhance translation while the red arrow indicates ASFV proteins that inhibit translation.

The protein synthesis of ASFV is host translation machinery-dependent. Viral translation proteins including pI215L, pE66L, MGF 110-7L, pA224L, pDP71L, pEP424R, pD250R, and pI73R are synthesized by exploiting the host cellular translation apparatus and signaling pathways for viral gene expression (Figure 4, Table 2). ASFV mRNAs exhibit short extensions at the RNA-5′ end post-transcription. These extensions consist of 1–2 copies of an “AU” dinucleotide, which are template-encoded. mRNAs with this feature originate from genes beginning with the A*TA sequence, corresponding to the Inr promoter motif. The transcript slippage of the promoter-associated ASFV RNA polymerase likely generates these extensions.

ASFV hijacks the host translation machinery that can control the induction or suppression of over 2000 host protein synthesis. The entire mechanism is still unknown. However, the partial mechanism has been studied, which is being discussed. Firstly, upon infection with ASFV, the virus manipulates the environment of the host cell to create specialized compartments called viral replication compartments. Within the viral replication compartments, ASFV synthesizes the concentrated translation machinery to preferentially translate its own mRNA efficiently compared to the host mRNA. ASFV causes the accumulation of its own mRNA at the viral replication foci, ensuring a high concentration of viral mRNA for translation within these specialized compartments. The eukaryotic initiation factors (eIFs), such as eIF2, eIF4F, eIF4G, and eIF4E, play crucial roles in initiating the translation process. The eIFs bind with ribosomes to form ribosomal preinitiation complexes around the start codon [1,103]. Secondly, the eIF4F consists of three different subunits, eIF4E, eIF4G, and eIF4A, in which their functions are binding to the 5′ cap of mRNA, acting as a scaffold protein, and unwinding secondary structures in mRNA, respectively. ASFV controls the expression of these eIF4F complex components by manipulating various factors. These factors include host cellular factors, viral proteins, and host regulatory mechanisms. Ensuring the efficient production of viral proteins required for replication and propagation, this manipulation occurs during the early and late stages of infection. By modulating the levels of these components, ASFV enhances the initiation of viral mRNA translation [105]. Thirdly, the protein involved in this process is the early viral ubiquitin-conjugating enzyme, pI215L. It binds to eIF4E, resulting in the overexpression of eIF4E. I215L also binds with Cullin 4B (CUL4B), a component of the Cullin-RING ubiquitin ligase (CRL4) complex involved in the ubiquitination and degradation of target proteins, and which influences regulation of mTOR (mechanistic target of rapamycin) activity, thereby manipulating cellular pathways involved in protein synthesis and other processes, promoting conditions favorable for viral replication and propagation. 

At late stages of infection, the ASFV protein pA224L induces the expression of eIF4G1 and eIF4E subunits of the eIF4F complex [106,107]. The ASFV protein pA224L shares structural and functional similarities with IAPs, and thus acts to inhibit apoptosis in infected cells. Within the eukaryotic translation initiation complex eIF4F, eIF4G serves as a scaffold protein binding mRNA to the ribosome. Additionally, A224L binds with caspase-3, a key executioner caspase involved in apoptosis, thereby inhibiting degradation of eIF4G. This prevents disruption of translation initiation, thereby increasing protein synthesis [108]. eIF2α, a key regulator of translation initiation, participates in the assembly of the translation pre-initiation complex and in the recognition of the start codon on mRNA. However, ASFV can modulate the activity of eIF2α (via phosphorylation), thereby causing host translation arrest, which can aid in translation of viral mRNA, ensuring efficient viral protein synthesis even in the presence of host cell stress responses [109].

The proteins *E66L*, *MGF 110-7L*, and *DP71L* interact with eIF2α by different mechanisms to enhance ASFV translation. *E66L* phosphorylates eIF2α interacting with protein kinase R, resulting in host translational arrest. *MGF110-7L* works with *E66L*, inducing ER stress and thereby enhancing eIF2α phosphorylation, which subsequently activates the kinases PERK and PKR. Cellular proteins such as PDIA3, PSMA4, and TMED4 were identified as interactors of the *MGF110-7L* protein; however, their roles remain unknown [109]. In contrast, *DP71L* promotes viral protein synthesis and translation by targeting protein phosphatase 1 (PP1) to dephosphorylate eIF2α [107]. ASFV employs strategies beyond interacting with eIF2α to manipulate the host cell translation machinery. These strategies are utilized to promote viral protein synthesis while simultaneously inhibiting host protein synthesis. ASFV facilitates phosphorylation of 4E-BP (4E-binding protein) to release eIF4E in early stages, resulting in formation of the eIF4F complex and enhancing viral protein translation. In late stages, ASFV promotes the dephosphorylation of 4E-BP, inhibiting translation and thereby regulating the timing of viral protein synthesis during different stages of infection [77]. ASFV manipulates host cell histones, particularly histone H3, by inducing a hypoacetylated state of lysine residues at positions 9 and 14 (K9/K14). This alteration promotes viral gene expression by restructuring chromatin, thereby facilitating access to viral DNA for transcriptional machinery. *EP424R* encodes pEP424R, which produces a protein resembling FTS-J-like RNA methyltransferase. This protein likely stabilizes ribosomal RNA (rRNA) in host cells, preventing the shutdown of protein synthesis machinery. By maintaining rRNA stability, ASFV ensures the continuous production of viral proteins necessary for viral replication [1]. 250R (g5R) reduces the number of transcripts and inhibits viral translation. It is also involved in the degradation of the host cellular mRNAs. Furthermore, the small RNA-binding protein, I73R, facilitates the withholding of cellular mRNAs within the nucleus. Additional protein–protein interactions (PPIs) between ASFV proteins and host translation factors, including ribosome and ribosome-associated proteins and mRNA-binding proteins, for instance, have been identified, but their functional significance is still unknown [107].

#### 3.3.3. Replication

After reaching the replication site near the microtubule organizing center (MTOC) in the perinuclear region, the uncoated ASFV virus particles move to the cytoplasm to proceed with their replication [95]. Small DNA fragments of ASF are initially synthesized in the nucleus through replication and then channeled to the cytoplasm where large DNA fragments are formed and matured into cross-linked DNA. ASF replication in the nucleus causes viral disruption of the nucleolus and nuclear membrane, followed by the phosphorylation and degradation of laminin A/C. This leads to the recruitment of produced membrane fragments to the replication site and the positioning of nucleoporin p62 at its periphery [26]. ASFV replication is mostly through its own enzymes and gene expression. The replication takes place immediately after the viral core enters the cytoplasm. The viral DNA replication enzymes are DNA polymerase, DNA ligase, DNA topoisomerase, DNA-binding protein, and DNA helicase [81].

**Figure 5:**
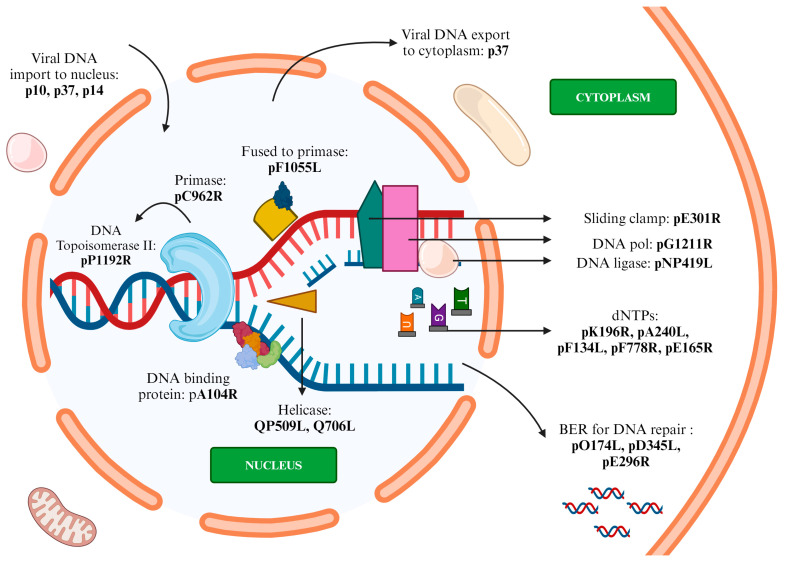
The proteins encoded by ASFV genes that are involved in replication. dNTPs—deoxynucleotide triphosphate; BER—base-excision repair; DNA pol—DNA polymerase.

The proteins involved in replication include pC962R, pG1211R, pQP509L, pQ706L, pA104R, pP1192R, p10, p37, p14, pF1055L, pE301R, pK196R, pA240L, pF134L, pF778R, pE165R, pO174L, pD345L, and pE296R, encoded by the genes *C962R*, *G1211R*, *QP509L*, *Q706L*, *A104R*, *P1192R*, *K78R*, *CP2475L*, *CP2475L*, *F1O55L*, *E301R*. *K196R*, *A240L*, *F134L*, *F778R*, *E165R*, *O174L*, *D345L*, and *E296R*, respectively (Figure 5, Table 3).

The ASFV genome structure closely resembles that of poxviruses. Replication intermediates, such as head-to-head concatemers, suggest a model similar to that of the vaccinia virus. In this model, replication begins with the generation of a single-stranded nick on either one or both ends of the DNA by the primase *C962R*. This process exposes the 3′-OH, which acts as a primer to facilitate replication. *C962R* encodes nucleoside triphosphatase (NTPase) homologues to poxvirus primase (D5) protein, which are involved in replication initiation, lagging strand synthesis, and pattern change. This results in head-to-head concatemers formation, with the ends of the nascent and template strands being self-complementary and folded. These ends create a self-starting hairpin structure that initiates DNA replication. These concatemers develop into terminally cross-linked matured genomes and are packaged into viral factories [1]. The two most important proteins involved in replication are the DNA-binding proteins, pA104R and p10, with the former having higher affinity. pA104R, located in the viral replication site, works together with DNA topoisomerase II (pP1192R) to prevent DNA supercoiling. It plays multiple roles in replication, transcription, genome agglutination, and separation, which catalyze dsDNA transient nicking, thereby promoting translation. This DNA topoisomerase II (pP1192R) is expressed in the porcine macrophage cytoplasm during replication. It then relocates to the viral factory, where it becomes involved in replication processes [73]. p10, encoded by K78R, is a DNA-binding protein that localizes in the nucleus along with p14. p10 plays a key role in the late stage of viral replication, performing active nuclear import by making use of the cellular mechanisms [42]. p37 and p14, produced by the catalyzation of pp220 (pCP2475L) by pS273R, are ASFV-encoded nucleocytoplasmic shuttling proteins involved in nuclear transport. While p37 participates in the transport of viral DNA from the cytoplasm to the nucleus and then to the cytoplasm, p14 nuclear transport is restricted to cytoplasm to nucleus only [65]. This p37 nucleocytoplasmic shuttling is a process mediated by CRM-1 receptors [110]. pF1055L protein homolog to herpes virus UL-9 protein is a helicase and primase that binds to the replication origin and functions as a part of DNA replication initiation. DNA polymerase type A (ORF G1207R), DNA polymerase family B (G1211R), and PCNA-like protein (E301R) act as sliding clamps and participate in replication initiation [73]. Two RNA helicases, QP509L and Q706L, are involved in the synthesis of viral particles and play non-redundant roles in the replication and transcription during the middle and late stage [111]. ASFV uses the base-excision repair pathway for damage repair caused by the free radical abundant macrophage (hyperoxia) and ensures the integrity of the ASFV genome. This repair is facilitated with the DNA glycosylase of the host and its own damage repair system, which includes ASFV-encoded ATP-dependent DNA ligase (pNP419L), PCNA-like protein E301R, Pol X (pO174L), 5′/3′ exonuclease D345L, and AP endonuclease (pE296R) (82). Thymidine kinase (pK196R), thymidylate kinase (pA240L), ribonucleotide reductase subunits (pF134L, pF778R), and uracil deoxyribonucleoside triphosphatase dUTPase (pE165R) increase dNTPs for replication. pE165R has high fidelity and minimal replication errors in a low dUTP environment. pI215L protein (E2 ubiquitin-binding enzyme), besides its role in transcription, also plays a part in ASFV replication [73]. Recent research on genotype II Georgia 2007/1 isolate with deletion of specific MGF360 and MGF505 genes, along with a negative serology marker (K145R), led to reduced replication rates in macrophages and attenuated virulence in pigs [112]. The deletion of MGF360-12L significantly reduced virus replication in macrophages, highlighting its importance for maintaining high replication levels [112].

### 3.4. Viral Assembly and Release

In the process of viral assembly, the newly synthesized viral components amalgamate to become mature virions. The assembled mature virions are then released from the host cell to initiate infection in neighboring cells or new hosts. ASFV achieves viral infection by changing the trans-Golgi endosomal network (TGN) system. The small GTPase ADP-ribosylation factor 1 (Arf1) is manipulated by GTPase-activating proteins (GAPs) and guanine nucleotide exchange factors (GEFs). This manipulation results in the recruitment of the host adaptor protein (AP-1) for transporting proteins from the TGN to the endosome. AP-1 facilitates formation of clathrin-coated vesicles by recruiting clathrin; these vesicles selectively distinguish cargo based on the sorting signals situated at the cytoplasmic tail of integral membrane proteins. Tyrosine (YXXF) and the di-leucine ([D/E]XXXL[L/I]) motif are the two sorting signals reported to be recognized by AP-1. CD2V, through this di-leucine motif, interacts with AP-1, and this results in the new membrane structure formation [79].

Virion replication, assembly, and morphogenesis take place in the viral factory of the cytoplasmic perinuclear region located in the MTOC [44]. Viral factory formation is microtubule-integrity-dependent. Lacking cell organelles and an external restriction membrane, VF is encircled by vimentin, forming a cage-like scaffold to prevent entry of viral particles to the cytoplasm. Invaginated ER is found at the VF terminal [113]. Viral morphogenesis in the VF progresses with infection. At an earlier stage, the virus exists as unlinked small fragments derived from the ER, which evolves to a curved membrane structure. The curved membrane structure further develops into spherical virion precursors with assembled capsid and nucleocapsid domains, and finally aggregates the intermediates to form an icosahedral network structure [51]. VF contains viral genes, host and viral proteins, virus particles (immature and mature), viral replicase, and precursor membrane fragments from ER [95]. The newly synthesized icosahedral virions migrate from the viral factory towards the plasma membrane through microtubule-mediated transport and are released [107].

**Figure 6:**
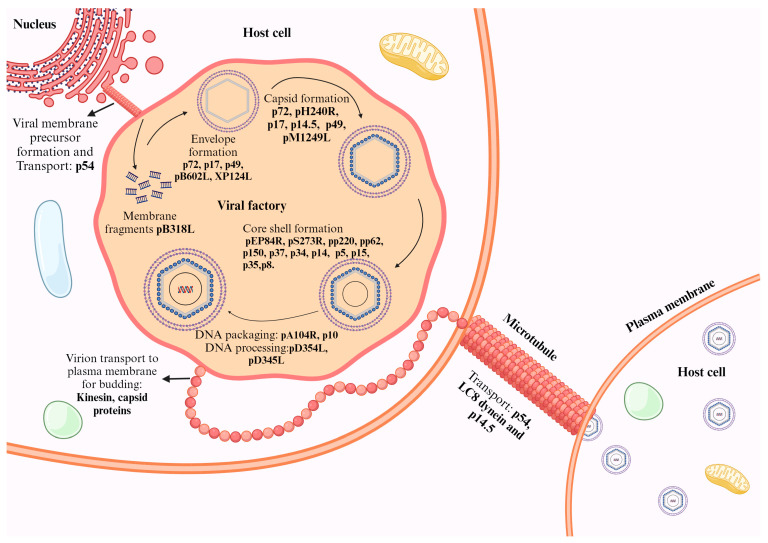
The proteins encoded by ASFV genes that are involved in the assembly and release of ASF virions.

The ASFV proteins involved in the mechanism of viral assembly and release include pA104R, p10, pEP84R, pM1249L, pB318L, pB354L, pB602L, pD345L, pS273R, pH240R, pE120R, p72, p49, p54, p17, p150, p34, p35, p15, and p8, encoded by the genes *A104R*, *K78R*, *EP84R*, *M1249L*, *B318L*, *B354L*, *B602L*, *D345L*, *S273R*, *H240R*, *E120R*, *B646L*, *B438L*, *E183L*, *D117L*, and *CP2475L (p150*, *p34*, *P35*, *P15*, *P8)*, respectively (Figure 6).

The correct assembly relies on several key proteins: p17 (*D117L*), p54 (*E183L*), the major capsid protein p72 (*B646L*) and its chaperone *B602L*, the minor capsid protein p49 (*B438L*), and 15 of the 23 polyproteins pp220 (*CP2475L*) and pp62 (*CP530R*). The proteolytic products of these polyproteins are core-shell components. The interaction between *EP84R* and *CP2475L* drives the start of core-shell formation by channeling the target membrane of the core-shell polyproteins (75). The interaction between polyproteins pp220 and pp62 forms a core shell beneath the internal lipid envelope. This core shell is created using core-shell proteins deposited on the membrane assembly. pp62 and pp220 are pivotal for the assembly and maturation of the viral core [81]. Myristoylation of pp220 plays an important role in membrane anchoring signal transduction by pp220 between the inner membrane and core shell, which are closely attached in their immature form. Upon maturation, the membranes separate as the core shell and inner membrane layers within the capsid. In parallel to the myristoylation of pp220 and assembly of viral particles, ASFV SUMO-like S273R protease-mediated hydrolysis of pp220 and pp62 takes place, where proteolytic hydrolysis cleaves them to become mature virions and be fitted into the core shell [59].

The ASFV capsid comprises pseudo-hexameric and pentameric capsomers. The pseudo-hexameric capsomers are made of three p72 molecules. Pentameric capsomers have five copies of the penton protein H240R. A complex network formed by H240R, p17, p49, and M1249L is located immediately below the outer capsid shell, stabilizing the entire capsid [84]. The homotrimeric structure of p72 consists of each monomer adopting a double jelly-roll configuration. The assembly of the capsid commences as the penton complex docks onto the inner membrane, leading to the recruitment of p72 capsomers to establish the penton core. M1249L then binds to the penton core, while zippers are formed by the interaction of p72 capsomers and p17. Zippers link neighboring penton cores, thus constructing a polyhedral structure. Completion of the framework involves filling trisymmetrons with p72 capsomers, finalizing the capsid assembly [42]. p17 securely anchors p72 capsomers to the inner membrane, ensuring the stability of the capsid. Halting the proteolytic processing of pp220 and pp62 by suppressing the p17 gene leads to the formation of core defective icosahedral particles [73,114]. A non-structural protein, pB602L, encodes a chaperone that mediates folding of the capsid major protein p72 (B646L) and the capsid apex protein p49 (B438L), resulting in the formation of the capsid structure. p49 is also a membrane protein [81]. Lacking p49, the virion was found to have an abnormal tube-like structure [35]. Capsid assembly is mediated by an interaction between E120R and B646L (p72). In the cytoplasm, p72 binds to the membrane of the endoplasmic reticulum (ER) and forms a capsid on the ER membrane’s convex surface. The inner layer of the core shell lies on its concave surface. When the p72 protein of ASFV is inhibited, the pp220 and pp62 proteins cannot undergo proper processing. This leads to the formation of an irregular zipper-like structure. Additionally, the processing of pp62 depends on the presence of the pp220 precursor. This highlights the importance of p72 [73]. Recent revelations about the capsid structure of ASFV highlight the interaction between the previously uncharacterized protein M1249L and both minor and major capsid proteins, namely D117L (p17) and B646L (p72), respectively. These interactions are pivotal in shaping the zipper-like structure of the capsid framework and in determining the overall size of the capsid [107].

The proteolysis products of pp220, namely p34 and p150, are membrane-associated components capable of forming viral matrix structures. Properly processed p150 products are specifically concentrated at the inner capsule membrane. Inhibition of the proteolytic processing of pp62, which includes p35, p15, and p8 protein products, leads to the occurrence of mostly empty virions [73]. p10 (pK78R) and pA104R are DNA-binding proteins located in the core shell and involved in genome packaging [73]. XP124L expression contributes to the enveloping of ASFV [115]. p54 plays a role in collecting ER and facilitating its conversion into the viral membrane precursor. packaging A32L ATPase (B354L) and lambda-like recombinase (D345L) are studied for their involvement in processing DNA ends during recombination, facilitating strand exchange or single-strand annealing [72]. The B318L gene encodes a trans-pentenyl transferase located at the viral assembly site. The association of this pB318L with the precursor viral membrane from the ER is potentially involved in virus factory formation and/or viral assembly [73].

Nucleoid formation is considered to be the last step of virus assembly [81]. Mature virions depend on both kinesin and ASFV capsid proteins for transportation from the viral factory to the cell membrane. The virions exit the host cells by budding through microtubule transport facilitated by p54 [73]. Additionally, P14.5 (pE120R) aids this process. The molecular mechanism of microtubule-mediated virus transport is elucidated by the direct binding of virus structural proteins with the small molecular dynamic complex [1]. Eventually, the virions are released from the cells through budding, acquiring an outer membrane in the process. As a result, extracellular viral particles gain a plasma membrane layer [81].

## 4. Apoptosis Inhibition

ASFV employs protein-driven mechanisms to inhibit apoptosis, allowing the virus to evade host defensive mechanisms, and promoting the replication process and survival of the virus. The ASFV apoptosis inhibitor proteins include pA224L, pEP153R, and pA179L, encoded by the genes *A224L*, *EP153R*, and *A179L*, respectively. Firstly, pA224L triggers the activation of the NF-κB signaling pathway within infected cells. Upon activation, NF-κB translocates into the nucleus. It subsequently binds to specific DNA sequences in the promoter regions of target genes, including those encoding antiapoptotic proteins facilitating cell death evasion [116]. Secondly, pEP153R, a C-type lectin domain containing protein, also has a similar function. The protein interacts with p53, an apoptotic cascade regulator. This interaction causes a reduction in its transactivating activity. This ability to activate the expression of target genes is involved in apoptosis and other cellular processes [117]. Thirdly, *A179L* has similar apoptosis inhibitor functions to those of pEP153R for facilitating viral proliferation [42].

## 5. Immunomodulation

ASFV targets porcine macrophages. The innate immune system serves as the primary line of defense against pathogens, subsequently initiating the adaptive immune response. The pathogen-associated molecular patterns (PAMPs) of ASFV include DNA and RNA. These PAMPs are recognized by the host pattern recognition receptors (PRRs). This recognition activates signaling pathways that induce the innate immune response. This response leads to the production of type I interferon (IFN-Iβ), pro-inflammatory cytokines, and chemokines. The main effectors of innate immunity are macrophages, plasmacytoid dendritic cells (pDCs), and NK cells. pDCs and NK cells release a large number of IFNs and inhibit ASFV infection progression. Macrophages release cytokines (IL-1a, IL-1b, and IL-18) and express MHC I, which activates an adaptive immune response. Both humoral and cell-mediated immune responses are then observed [1].

To resist the immune response and survive and proliferate in the host, ASFV has evolved immune-evasion or immune-suppression strategies. These strategies involve numerous genes and their encoded proteins. Their purposes are to counteract the immune response mechanisms of the host.

### 5.1. Inhibition of cGAS Sting Pathway

In the case of mammals, cytosolic detection of nucleic acids is essential in stimulating the innate system response against viruses. This detection and response are mediated by several adaptor molecules, and endosomal and cytosolic sensors.

In the case of ASFV, the main PRR is the cytosolic DNA sensor cGAS. cGAS (cyclic GMP-AMP (cGAMP) synthase) senses the cytosolic ASFV dsDNA, leading to the production of cGAMP, which leads to subsequent signaling via STING, TBK1, and IRF3, ultimately resulting in the production of type I IFN-β.

cGAS, after sensing dsDNA of ASFV, synthesizes cGAMP, which binds and triggers the adaptor–stimulator of interferon genes (STING). STING moves from ER to the Golgi to the ER–Golgi intermediate compartment (ERGIC), and recruits and phosphorylates TANK-binding kinase 1 (TBK1). TBK1 subsequently phosphorylates IRF3, which then translocates to the nucleus and acts as a transcription factor, resulting in type I IFN-β expression [118].

In a study carried out by García-Belmonte et al. (2019), it was concluded that the virulence of ASFV strains is proportional to their ability to inhibit the type I IFN-β production in the cGAS-STING pathway, thereby controlling the innate immune response [119].

Several ASFV genes and the encoded proteins were found to inhibit the cGAS-STING pathway. These genes include S273R, DP96R, D117L (p17), EP364R, C129R, MGF-505-7R, MGF-505-11R, MGF360-11L, E301R, I215L, MGF360-14L, MGF360-13L, MGF505-3R, L83L, E120R, I226R, M1249L, A137R, E184L, CD2v, QP383R, and MGF505-2R. By inhibition of this pathway, the type I IFN-β is not produced, and hence the innate immune system is suppressed.

The ASFV pS273R protein inhibits the cGAS-STING pathway by co-transfection in 293T cells, which leads to subsequent suppression of ISRE, IFNβ, and NF-κB promoter activity, resulting in failure to elicit an immune response. The presence of pS273R restricts phosphorylation activity of IRF3 and TBK1, thereby causing immunosuppression. It also mediates immunosuppression through interaction with STING and IKK-ε [62]. DP96R restricts the activation of the promotors IFN-β and ISRE, thereby suppressing TBK1 phosphorylation. p17 interacts with STING and inhibits the recruitment of TBK1 and IKKϵ in the cGAS-STING pathway [49]. *EP364R* and *C129R* interact with cGAMP and exert their phosphodiesterase (PDE) activity to cleave 2′,3′-cGAMP blocking cellular cGAMP-mediated antiviral responses [120]. *MGF-505-7R* and *MGF-505-11R* interact with STING and inhibit its expression via autophagy [121]. *MGF360-11L* interacts via the pathways of cysteine, ubiquitin-proteosome, and autophagy, and breaks down TBK1 and IRF7. TBK1 is critical for innate immune signaling, while IRF7 regulates type I interferon responses. Within these pathways, *MGF360-11L* exerts an influence by targeting the degradation of two pivotal proteins, TBK1 and IRF7. By manipulating cysteine metabolism, *MGF360-11L* may impact immune responses, given the essential role of cysteine in cellular processes. Additionally, it interacts with the ubiquitin–proteasome system, leading to the degradation of TBK1 and IRF7. Moreover, *MGF360-11L* likely modulates autophagy to enhance the turnover of TBK1 and IRF7 proteins, potentially impairing host immune defenses and facilitating viral replication, and thereby inhibiting phosphorylation of TBK1 and IRF3 stimulated by cGAS-STING expression. Amino acid residues 167–353 of ASFV *MGF360-11L* can inhibit activation of the IFN-β and ISRE promoters [122]. pE301R interacts with IRF3, hindering phosphorylation, and thereby restricts its translocation by cGAMP and poly(dA:dT), thus suppressing the pathway [123]. I215L binds with RING finger protein 138 (RNF138) and causes a degradative interaction with RNF138 and RNF128, thus inhibiting type I IFN production and K63-linked polyubiquitination of TANK-binding kinase 1 [124]. Similarly, *MGF360-14L* causes K63-linked ubiquitination of IRF3 by interaction with E3 ubiquitin ligase TRIM21, thereby degrading IRF3 and inhibiting type I IFN production [125]. *MGF360-13L* and *MGF505-3R* downregulate the cGAS-STING pathway by degrading STING and TBK1, respectively [126,127]. Through interaction with cGAS and STING, *L83L* mediates autophagy-lysosomal degradation of STING by recruiting TOLLIP. This process blocks the phosphorylation of TBK1, IRF3, and IκBα, consequently reducing IFN-I production [128]. *E120R* and *I226R* bind with IRF3, thereby hindering phosphorylation, while M1249L degrades IRF3 via the lysosomal pathway, both suppressing the cGAS-STING pathway [125]. *A137R* degrades TBK1 by the autophagosome and lysosome-dependent pathways [129]. *E184L* interacts and inhibits the dimerization and oligomerization of STING, restricting the expression of IFN-β and IL-1β. CD2v interacts with STING by blocking its translocation from ER to the Golgi, thus preventing STING activation [107]. QP383R influences cGAS-STING-mediated IFN-I production through the suppression of steps upstream of STING, which interact directly with cGAS. *QP383R* can trigger elevation of palmitoylation levels of cGAS, directly affecting its DNA binding and enzymatic activity. This elevation stimulated alongside poly(dA:dT) inhibits the DNA binding, dimerization, and the enzymatic activity of cGAS [130]. *MGF505-2R* interacts with the STING protein, which regulates IFN-β production. *MGF505-2R* considerably decreases the expression of ISG and IFN-β and phosphorylation of TBK1 [131].

### 5.2. Inhibition of NF-κB Pathway

Once the PAMPs are recognized by PRRs, several innate signal transduction pathways are triggered by a variety of adaptor proteins which phosphorylate transcription of IRF3. However, some pathways activate the transcription factor nuclear factor-kappa B (NF-κB). Upon activation, NF-κB translocates to the nucleus and expresses proinflammatory genes including cytokines and chemokines. IFN-B is also expressed via an autocrine loop mediating IRF7 expression and production of IFN-α subtype genes—IFN-I(α/β), which binds to receptors and initiates the JAK-STAT pathway [132].

NF-κB activation occurs via two major signaling pathways, i.e., canonical and non-canonical. The canonical pathway facilitates the activation of NF-κB1/p50 and RELA/p65, collectively known as canonical NF-κB family members. This pathway is initiated by signals, including those transmitted by innate and adaptive immune receptors. These signals lead to the activation of the IKK complex, consisting of catalytic IKKα and IKKβ subunits, along with regulatory IKKγ subunits and NEMO, by TAK1 through IκBα phosphorylation. Subsequently, IκBα undergoes degradation, resulting in the rapid and transient nuclear translocation (by KPNA2, KPNA3, and KPNA4) and activation of heterodimers NF-κB1/p50 and RELA/p65. This heterodimer activation regulates expression of proinflammatory and cell survival genes [133].

ASFV proliferates and infects the host macrophages by inactivation of the NF-κB pathway through various immunomodulatory proteins, including pA238L, pD345L, MGF505-7R, pF317L, MGF360-12L, pH240R, pEP402R, pDP96R, pA224L, pI329L, pI226R, pK205R, and pE120R.

*A238L* hinders NF-kB interaction with the p65 subunit of NF-kB by inhibiting CBP/p300 co-activators. *D345L*, interacts and disrupts the kinase activity of IKKα and IKKβm, thus suppressing NF-κB activation [134]. *MGF505-7R* interacts with IKKα, inhibiting NF-κB activation and NF-κB-mediated IL-1β transcription. Additionally, F317L reduces the phosphorylation of IKKβ, consequently decreasing the phosphorylation and ubiquitination of IκBα. On the other hand, *MGF360-12L* interacts with nuclear transport proteins KPNA2, KPNA3, and KPNA4, diminishing their nuclear transport function. This disruption in binding between p65 and these nuclear transport proteins results in NF-κB inactivation. Overexpression of cGAS-STING, TBK1, and IKKβ can activate NF-κB, and *MGF-360-12L* can prevent this. *H240R* specifically interacts with and facilitates the autophagic degradation of NF-κB essential modulator (NEMO), a component of the IKK complex. This process results in the inhibition of p65 and IκBα phosphorylation, ultimately impairing NF-κB activation. Additionally, *EP402R*, present on infected cell membranes or released by ASFV-infected macrophages, interacts with CD58, leading to NF-κB activation and induction of IFN-β [107]. *DP96R* also affects the cGAS/STING-mediated NF-κB signaling by blocking the activation of TBK1 and IKKβ. By inhibiting IκB kinase (IKK), cGAS-STING, and TANK-binding kinase 1 (TBK1), *DP96R* inhibits NF-κB. IKK is involved in activating NF-κB by phosphorylating inhibitor of nuclear factor kappa B protein (IκB), causing its degradation and releasing NF-κB. *DP96R* hinders this process by inhibiting IKK, preventing NF-κB activation. Additionally, *DP96R* inhibits cGAS-STING, interfering with the signaling needed for NF-κB activation in response to cytosolic DNA. Furthermore, TBK1, a kinase involved in NF-κB activation, phosphorylates IKKε, which activates IKK and promotes NF-κB activation. By inhibiting TBK1, *DP96R* disrupts this signaling cascade, ultimately inhibiting NF-κB activation [135]. pA224L is also found to inhibit T-cell activation through the NF-κB pathway [117]. The intracellular domain (ICD) of pI329L interrupts the adaptor protein TRIF, which is recruited by TLR3 and TLR4 for their signaling cascade pathways. This indicates the inhibition potential of IFN-β and NF-κB by this gene, but via different mechanisms [136]. *I226R* regulates the NF-κB signaling pathway by the inhibition of upstream regulatory molecules like RIG-I, MAVS, RIP1, TRAF2, TRAF6, and IKKβ, and suppresses phosphorylation of p65, a downstream molecule of the NF-κB signaling pathway [137]. Following proinflammation activated by ER stress, *K205R* phosphorylates NF-κB subunits (IκBα and P65) and upregulates the transcription of proinflammatory cytokines like *Il-6*, *Il-18*, and *Tnfa.* This gene prevented the nuclear translocation of p65 and p50, and inhibited the TBK1-, IKKβ-, and p65-mediated NF-κB pathway activation [138].

### 5.3. Inhibition of JAK-STAT Pathway

After release from the cGAS-STING pathway, interferons regulate transcription of interferon-stimulated gene segments (ISGSs) by triggering Janus Activated Kinase and Signal transducer activator of transcription (JAK-STAT) to create an antiviral state within the host. IFNs include IFN-I, IFN-II, and IFN-III, and JAKs include JAK-I, JAK-II, JAK-III, and TYK2. IFN-1 and IFN-III bind to the type 1 IFN cell surface receptor complexes, IFNAR1/2 and IL10RB/IFNLR1, respectively. This leads to activation of TYK-2 and JAK-1, which phosphorylates STAT1 and STAT2. These two compounds interact with IRF9, forming an STAT1-STAT2-IRF9 complex referred to as interferon-stimulated gene factor-3 (ISGF-3). ISGF-3 translocates to the nucleus and binds to the promotor region of interferon-stimulated response elements (ISREs) to initiate transcription of ISGs, thereby creating an antiviral state. When IFN-II binds to its receptor complex IFNGR1/2, it initiates the activation of JAK1 and JAK2, resulting in the phosphorylation and homodimerization of STAT1. 

The homodimer translocates to the nucleus and attaches to IFN-γ-activated sequence (GAS) elements, initiating the transcription of ISGs and establishing an antiviral state [107].

The ASFV genes involved in the inhibition of JAK-STAT pathway include *MGF505-7R*, *MGF360-9L*, *MGF360-10L*, *I215L*, *H240R*, *EP402R*, *I267L*, and *A104R.*

By upregulating the expression of the E3 ubiquitin ligase RNF125 and downregulating the expression of Hes5, *MGF505-7R* achieves the degradation of JAK1 and JAK2, thus suppressing the activation of IFN downstream signaling. As a result, the activation of the IFN-induced JAK-STAT signaling pathway is inhibited [139]. *MGF360-9L* binds and inhibits STAT1 via the ubiquitin–proteasome pathways [140]. *MGF360-10L* acts as a dose-dependent mediator in the JAK1 degradation process [141]. I215L interacts with IRF9 for autophagic degradation independently of the ubiquitin-conjugating activity [125,142]. pH240R interacts with IFNAR1 and IFNAR2 to disrupt the interaction of IFNAR1-TYK2 and IFNAR2-JAK1, respectively, resulting in the suppression of the import of STAT1 and STAT2 to the nucleus and ISG expression [143]. In a similar mechanism, *EP402R* (CD2V) disrupts the formation of the IFNAR1-TYK2 and IFNAR2-JAK1 complexes. Meanwhile, *S273R* interacts with STAT2, recruiting E3 ubiquitin ligase DCST1. This interaction results in the K48-linked polyubiquitination of STAT2, leading to its proteasomal degradation [107]. It also interacts with CSF2RA to downregulate the JAK2-STAT3 pathway and promotes apoptosis to inhibit ASFV replication [144]. pI267L facilitates degradation of IRF9 and STAT2 [129]. pA104R is also involved in JAK-STAT pathway. It antagonizes the IFN-I-triggered signaling pathway by attenuating STAT1 phosphorylation. This results in epigenetic modifications contributing to pA104R pathogenicity. This identifies a novel role for pA104R in ASFV evasion of host innate immunity [74].

### 5.4. Inhibition of NFAT Pathway

Transcription factor–NFAT proteins are modulated by calcium ions. An increase in the calcium ion level in the cell occurs upon exposure to different stimuli such as TCR activation. This activates the calcium/calmodulin-dependent phosphatase calcineurin, which dephosphorylates NFAT, leading to its translocation into the nucleus, where it becomes transcriptionally active to produce genes involved in immune response [145].

The ASFV protein actively involved in inhibition of this pathway is pA238L. pA238L impacts the phosphatase activity of calcineurin by interacting with its catalytic subunit, consequently inhibiting NFAT activation. Additionally, it has been demonstrated that pA238L suppresses the transcription of COX-2, a potent inducer of lipid-mediated inflammation, in an NFAT-dependent manner [107,117].

### 5.5. Inhibition of NLRP3 Inflammasome Activation

Inflammasomes are large cytosolic multiprotein complexes assembled in response to ASFV PAMPs. Key PRRs responsible for inflammasome activation include NLRs, such as NLRP3 and AIM2. NLRP3, comprising NACHT, LRR, and PYD domains, effectively detects viral invasion, initiating an inflammatory response. Activation of the NLRP3 inflammasome requires two signals. The first is the priming signal, which activates NF-κB, leading to the transcription of proinflammatory genes such as NLRP3, pro-IL-1β, and pro-IL-18. The second signal, called the activation signal, induces the NLRP3 inflammasome assembly. Activated inflammasomes, along with adaptor protein (ASC), lead to activation of caspase-1, which leads to synthesis of proinflammatory cytokines such as IL-1β and IL-18. Additionally, activated caspase-1 cleaves Gasdermin-D (GSDMD), which further associates with the plasma membrane and forms pores and mediates pyroptosis. The dead cells further signal damage-associated molecular patterns (DAMPS), which trigger enhanced immune activation and inflammation [107].

ASFV has evolved to evade inflammasome activity by inhibiting NLRP3 directly or indirectly through targeting NF-κB. Indirect inhibition of NLRP3 by targeting of NF-κB is previously discussed. In this section, we discuss the direct targeting of NLRP3. The genes involved in this inhibitory mechanism include *H240R*, *S273R*, and *MGF505-7R*. *H240R* interacts and suppresses NLRP3 oligomerization, which results in decreased formation of ASC (adaptor protein) and NLRP3 oligomers. *MGF505-7R* interacts with NLRP3 through its NACHT and LRR domains. Its expression disrupts the formation of ASC specks triggered by the NLRP3 inflammasome [107,117]. Through non-canonical cleavage of swine GSDMD to form a shorter GSDMD-NT (N1~107), pS273R can inhibit pyroptosis. Unlike the canonical GSDMD-NT (N1~279) produced by caspase-1, GSDMD-NT (N1~107) loses its pore-forming activity on the cytomembrane and is unable to induce pyroptosis. Therefore, one could reasonably speculate that GSDMD-mediated pyroptosis is significantly inhibited during ASFV infection [47,129].

## 6. Virulence Genes of ASFV

While almost all structural and non-structural genes of the ASFV genome are predicted to assist virulence, current and ongoing research has shed light on a few of the genes involved with their mechanisms, and the impact of their deletion on different ASFV strains (live attenuated vaccines, or LAVs). In this article, such genes have been categorized into the mediators of immunomodulatory pathways, mediators of apoptosis and cell autophagy, mediators of infection and other functions, and multifunctional proteins. For curated vaccine strategies, the virulent genes, as highlighted in this section, become crucial for detailed studies (Supplementary Table S2).

### 6.1. Mediators of Immunomodulatory Pathways

#### 6.1.1. A151R

The *A151R* gene is associated with viral replication and the thioredoxin pathway due to similarities in its cysteine residues [146]. It has been reported that the H102, C109, C132, and C135 amino acid residues in pA151R are responsible for negatively regulating the cGAS-STING-mediated production of IFN-β. Through the inhibition of K63-linked polyubiquitination and phosphorylation of TBK1, this protein hinders both the STING-TBK1-IRF3 and STING-TBK1-NF-κB axes to suppress IFN-β [147].

In a study conducted by Keita et al. using siRNA to repress its expression in BA71V, the inhibition of viral replication was seen. The downregulation of p72 mRNAs was also observed, indicating a possible role in the transcription of the virus [146]. The mutant ASFV-G-∆A151R produced by Ramirez-Medina et al., lacking the *A151R* gene from the highly virulent ASFV-G strain, could replicate normally in vitro, indicating it is a non-essential gene. A drastic decrease in virulence was noticed in vivo, with the attenuated strain protecting 3 out of the 4 experimental pigs against ASFV-G [148].

#### 6.1.2. A238L

The protein encoded by *A238L* can inhibit NF-κB-p65 and the action of the calcium/calmodulin-dependent calcineurin phosphatase [134]. pA238L is shown to control the c-Jun transcription factor (Activator Protein-1 component). These inhibitory mechanisms control the CBP/p300 transcriptional coactivators pathway. This results in the suppression of immunomodulatory genes like TN*F*-α and cyclooxygenase-2 (COX-2) [149,150].

The non-essential nature of *A238L* was demonstrated by Neilan and others by deleting this gene from the Malawi Lil-20/1 strain. This mutant Δ5EL showed no difference in the growth kinetics in vitro or disease progression in vivo from its parental strain [151]. The single deletion experiment on the virulent E70 strain by Salguero et al. saw no significant reductions in viral replication or pathology when compared to the parent strain. A marked increase in the TNF-α mRNA levels, however, was observed in pigs infected with this E70ΔA238L mutant [152]. Another recombination strain, NH/P68ΔA238L, showed a significant reduction in clinical signs seen in the parental NH/P68 strain. It could protect the pigs against the genotype I virulent strain L60 but did not provide adequate protection against the virulent Arm07 [153].

Upon deletion of *A238L* in combination with CD2v from the Arm/07/CBM/c2 parental strain, researchers found that this Arm-ΔCD2v-ΔA238L mutant was successfully attenuated in every experimental pig. Moreover, this mutant could also provide complete immunity against the virulent Paju strain [154]. The single deletion of this gene from the parental ASFV-Ke only moderately attenuated this ASFV-Ke-∆A238L strain in comparison to the double-deletion mutant ASFV-Ke-∆EP402R∆A238L. ASFV-Ke-∆A238L still caused viremia and clinical signs in pigs, whereas ASFV-Ke-∆EP402R∆A238L could limit viremia with only mild clinical signs in the animals. Further, ASFV-Ke-∆EP402R∆A238L partially protected the pigs against a homologous challenge with ASFV-Ke [155].

CRISPR/Cas9 technology has also been used to make recombinant strains lacking the A238L gene. Recombinants were produced from the virulent Pol18/28298/Out111 strain (A238L1Δ and A238L2Δ), and the virulent ASFV-Kenya-IX-1033 (ASFV-Ke-ΔA238L). Due to the non-essential nature of this gene, there was no reduction in the growth kinetics of these mutants, leaving them with the scope to be tested in vivo [156,157].

#### 6.1.3. DP96R

*DP96R* is a highly conserved gene, recognized as an important virulence determinant. It is a non-essential gene coding for the protein p15 expressed early in infected macrophages [158]. A comprehensive study by Dodantenna and others reported that *DP96R* could inhibit the IFN-β promoter. A significant suppression of phosphorylated TBK1 and IRF3, and an ~80% impairment in the nuclear translocation of IRF3 in *DP96R*-expressing PAMs, could also be observed. Such inhibition shows the suppression of the IFN signaling pathway which would, in turn, affect cGAS-STING pathway-induced antiviral gene transcription [159].

Being non-essential, *DP96R*-deleted strains did not affect replication levels in macrophages or viral growth kinetics [22,160]. siRNA-targeted DP96R knocked-down ASFV has been shown to upregulate levels of IFN and ISG transcription in vitro [159]. It was found that pigs infected with the UK gene-deleted recombinant E70 (ΔUK) strain could survive, with viremia reduced by 100–1000 fold, and were completely protected against the parental E70 [160]. However, the single-deletion recombinant strain ASFV-G ΔUK made by Ramirez-Medina et al. did not change the virulence levels when compared to the highly virulent ASFV-G parent strain [161].

Deletion of the UK and NL (*DP71L*) genes from the attenuated strain OUR T88/3 (OUR T88/3ΔDP2) reduced its protective effects by 34% during a challenge with the fatal OUR T88/1 [162]. The UK gene was also deleted from the weakened ASFV-G Δ9GL. This ASFV-G-Δ9GL/ΔUK strain was attenuated in pigs even at doses as high as 10^6^ HAD_50_. This is the first mutant that could protect pigs against the parental ASFV-G “as early as 2 weeks postvaccination”, and the protection has been correlated with anti-ASFV antibodies observed in the serum [163]. Two recombinants have been produced for the UK gene from the lethal HLJ/18. The HLJ/18-CD2v&UK-del mutant lacking the UK and EP402R genes was partially attenuated in pigs. However, deletion of UK with the B119L gene resulted in this HLJ/18-9GL&UK-del mutant being attenuated in pigs, but it failed to protect against the parental strain [164]. Despite BA71∆CD2DP96R displaying delayed infection with lower viral titers in comparison to deletion experiments with other genes in the experiment, this strain neither improved the safety of the attenuated BA71∆CD2 nor protected against a heterologous challenge with ASFV-G [165]. The attenuated strain ASFV-GS-Δ18R/NL/UK, obtained by deleting the UK, DP148R (MGF360-18R), and NL genes from ASFV-G, could not only protect pigs against a homologous challenge with the parental strain, but also downregulate MGF110-7L expression [22].

#### 6.1.4. E120R (p14.5)

p14.5 is responsible for the proper dissemination of the virions [166]. Studies have revealed that this gene prevents the nuclear translocation of p65 and p50, and inhibits the TBK1-, IKKβ-, and p65-mediated NF-κB pathway activation. It also interferes with the IFN-β promoter activity through phosphorylation inhibition of key connectors like STING, TBK1, and IRF3 [167].

The complete deletion of the *E120R* gene by Liu et al. resulted in the virus not being able to be rescued, confirming the gene’s importance for replication. It was found that the 71-to-75-amino-acid section in p14.5 is its potent region for virulence, and the construction of the truncated strain FLAG-E120R-Δ72-73aa lacking the critical 72 and 73 residues in this region showed that the suppression of IFN-β promoter activation by this gene was suspended. Significant expression of IFN-β and ISG15 was seen in the early infection period for this FLAG-E120R-Δ72-73aa. The replication levels of this strain were also 10 times lower than those of its highly virulent parent strain ASFV CN/GS/2018 [168].

#### 6.1.5. I226R

*I226R* encodes for a protein expressed early and late post-infection. This protein has been noted to be similar to *A238L* in terms of its ability to significantly downregulate IFN-β production. This gene inhibits the cGAS/STING-mediated IFN-β and ISRE reporter activation, along with controlling the expression of ISGs like ISG15, ISG20, IFITM1, IFITM3, OASL, and IFIT1. Further, it was also observed that *A238L* could tamper with IRF3 phosphorylation.

*I226R* was also shown to regulate the NF-κB signaling pathway, as evidenced by the observed inhibition mediated by upstream regulatory molecules like RIG-I, MAVS, RIP1, TRAF2, TRAF6, and IKKβ, but not p65. The gene was also seen to suppress phosphorylation of p65, indicating its direct action on downstream components of the NF-κB signaling pathway, such as p65. Specifically, *I226R’s* inhibitory action on NF-κB was reported at the level of the IKK complex. It has been described further that this gene could specifically target the key protein NEMO (IKKγ) of the IKK complex through ubiquitination, without any significant effect on the IKKα and IKKβ protein components [137].

The well-known mutant SY18ΔI226R from the parental SY18 strain did not reduce replication in vitro but was completely attenuated in vivo, with no clinical signs in all inoculated pigs even after a high inoculation of 10^7^ TCID_50_. A robust antibody response was observed in these pigs 9 days post-inoculation, and a reduction in any apparent viremia was noticed. This mutant strain was also able to completely protect immunized pigs against the parental SY18 challenge [169].

#### 6.1.6. I267L

*I267L* is identified to code for a protein of virulent importance. Studies by Ran and others found that this gene can counteract the action of the PRR RIG-I, which recognizes the RNA that is transcribed from AT-rich ASFV DNA by DNA-directed RNA polymerase III (Pol-III). *I267L* can interact with the ligase Riplet. This interaction suppresses the Riplet-mediated K63-linked polyubiquitination, which is an essential post-translation modification required for RIG-I activation. The researchers found that *I267L* overexpression could hinder RIG-I-mediated *IFNB1* expression in in vitro experiments, independent of cGAS [170,171]. The *I267L*-deleted mutant ASFV∆I267L from CN/GS/2018 revealed the non-essential nature of this gene [170]. Further, this virulent gene is a hemorrhage regulator. Transcriptomic analysis of PAMs infected with this deletion mutant revealed the upregulated levels of the tissue factor F3 in a TNF-α-mediated manner, and the collagen-expressing genes COL1A1 and COL1A2. This indicates that this gene can inhibit the coagulation cascade mechanisms and may block the activation of platelets to affect the clotting process [172].

The mutant SY18ΔI267L produced by deleting this gene from the virulent SY18 strain proved to be unattenuated in vivo, as the infected pigs presented the same clinical signs as the parental strain [173]. The deletion recombinant ASFVΔI267L from the CN/GS/2018 strain was moderately attenuated in pigs, and their serum showed higher IFN-β levels. Further, this strain could induce a higher p30 response in the surviving pigs [170].

#### 6.1.7. I329L

*I329L* is highly conserved through strains of varying virulence, and encodes for a non-essential membrane protein. This protein exerts its viral activity by modulating the type I IFN response and pro-inflammatory cytokines. It was found that the extracellular domain plus transmembrane (ECDTM) region can arrest TLR3, TLR4, TLR5, TLR8, and TLR9 activation by obstructing the binding of cytosolic DNA to the dsDNA interaction domain (DID), or the dimerization of the TLRs via the putative dimerization domain plus transmembrane (PDDTM) region. The intracellular domain (ICD) of pI329L interrupts the adaptor protein TRIF, which is recruited by TLR3 and TLR4 for their signaling cascade pathways. This indicates the potential of this gene to inhibit IFN-β and NF-κB, but via different mechanisms [136].

A general trend can be seen for deletion experiments involving I329L not being an adequate attenuation strategy. The mutant OURT88/3ΔI329L from the attenuated OURT88/3 strain has displayed notably higher amounts of IFN-I. However, this strain neither reduced pathology in the inoculated pigs nor protected them against a challenge with OURT88/1. The researchers also produced the GeorgiaΔI329L from ASFV-G, but this mutant failed to be attenuated in pigs [174].

#### 6.1.8. I73R

The protein encoded by *I73R* has been recognized as a conserved early protein localized in the nucleus of cells at 6 hpi (bone marrow-derived macrophages). At later stages post-infection (8–48 hpi), this protein has been noted to translocate to the cytoplasm [175]. It is suggested to belong to the Zα-domain-containing protein family, due to its typical “winged helix-turn-helix domain with two antiparallel β-strands and three α-helices” and a high affinity for host ssDNA with a high-GC content. It was revealed that p173R is significantly capable of restraining the overall protein synthesis of the host. It also can suppress the transcription of the TNF-α (hsa04668) and the NF-κB (hsa04064) downstream signaling pathways, and prevent TNF mRNA from moving to the cytoplasm [176]. Very recently, transfection experiments with this gene also revealed distinctively lower levels of IFN-β mRNA produced through the cGAS-STING pathway. It was concluded that *I73R* interfered with the IFN-β promoter activity mediated by NF-κB and prevented the nuclear translocation of IRF3 [177].

Liu and others also performed a deletion experiment with this gene on the virulent ASFV-GZ to understand its impact in vivo. This ASFV-GZΔ*I73R* mutant was successfully attenuated in pigs, with significantly low viral replication, which was undetectable from 21 dpi. Pigs also only presented a mild fever for a short duration both after infection and after a homologous challenge, indicating complete protection from the virulent parent strain. This deletion mutant could also restore cellular protein levels, further confirming earlier findings. Apart from TNF-α, the proinflammatory cytokine IL-6 and helper T-cell (CD3^+^CD4^+^CD8^−^) levels were also significantly higher in pigs with the mutant when compared to before immunization. [176].

#### 6.1.9. L83L

*L83L* is a conserved, early-expressed gene in the genome. It encodes a protein that interacts with the cGAS-STING pathway and triggers STING for degradation through the recruitment of Tollip, diminishing IFN-β and ISRE expression levels. From the conclusions of the *L83L* knockdown experiment using siRNA, it also affects the levels of ISGs like IFNB1, ISG15, and ISG56 [128]. It was also observed that the proinflammatory cytokine IL-1β is a specific binding partner for *L83L* and the two co-localize in infected cells. When this gene was deleted from ASFV-G, the replication levels of this ASFV-G-ΔL83L recombinant were not significantly lessened in vitro. This mutant remained virulent when tested in pigs in comparison to ASFV-G [178].

#### 6.1.10. MGF505-2R

The cGAS/STING pathway is inhibited by the *MGF505-2R* gene. This inhibition considerably decreases the expression of interferon-stimulated genes (ISGs). *MGF505-2R* specifically interacts with the STING protein by impeding the phosphorylation of TBK1, hence disturbing the cGAS-STING-mediated IFN-β pathway.

Notably, *MGF505-2R* is one of the genes deleted in HLJ/18-6GD (deletion of *MGF505-1R*, *MGF505-2R*, *MGF505-3R*, *MGF360-12L*, *MGF360-13L*, and *MGF360-14L*), and HLJ/18-7GD (additional deletion of CD2v). Both of these mutants were completely attenuated in pigs. Upon being challenged with the parental HLJ/18 strain, the immunized pigs with these mutants were highly protected, with transient fever and low viremia being reported. HLJ/18-7GD could also protect commercial pigs against the parental strain at high doses of 10^6.5^ HAD50 [164].

The Arm/07-ΔMGF505-2R-GFP strain constructed from the virulent Arm07 shows the non-essential nature of this gene in vitro. This recombinant is also shown to be completely attenuated in the experimental pigs. The immunized animals were further found to be partially protected against the parental Arm/07/CBM/c2 strain and the circulating ASFV/Korea/pig/PaJu1/2019 strain [131].

#### 6.1.11. MGF505-3R

*MGF505-3R* suppresses TBK1, IRF3, and IκBα phosphorylation. It has been found that this gene can significantly lower ISRE and IFN-β activity by reducing IFNB1 and IFIT2 mRNA levels. Mechanistically, this gene interacts with TBK1, IRF3, and cGAS, while targeting TBK1 for destruction [126]. This gene is a part of the HLJ/18-6GD and HLJ/18-7GD mutants, as mentioned before [164].

#### 6.1.12. MGF505-11R

*MGF505-11R* reduces the transcription of IFN-β, ISG15, and ISG56. It has also been noted to suppress IFN-β and ISRE activation caused by cGAS, IRF7, IRF3-5D, STING, IKKε, and TBK1. Through lysosomal, ubiquitin-proteasome, and autophagic pathways, *MGF505-11R* interacts with STING to attenuate its expression. Additionally, it prevents TBK1 and IRF3 from becoming phosphorylated when activated by cGAS/STING. Currently, vaccines are yet to be attempted for this gene [179].

#### 6.1.13. MGF360-9L

*MGF360-9L* interacts with JAK1, STAT1, and STAT2, three essential elements of the JAK/STAT pathway. *MGF360-9L* lowers the host’s capacity to develop a potent antiviral response by interfering with these proteins. The interferon (IFN) signaling cascade, which is crucial for antiviral defense, is interfered with by this gene, resulting in the diminished activation of the immune response’s downstream genes.

The ASFV-Δ360-9L recombinant developed from CN/GS/2018 showed a reduction in replication rates in vitro. Upon immunization, this mutant was found to be partially attenuated in pigs, with an 80% survival. These animals presented lower levels of the clinical signs and viremia for this mutant [140].

#### 6.1.14. MGF360-10L

*MGF360-10L* is known to primarily target the host protein JAK1 for degradation. This gene facilitates JAK1’s K48-linked ubiquitination at lysine residues 245 and 269. The E3 ubiquitin ligase HERC5 (HECT and RLD domain-containing E3 ubiquitin protein ligase 5) is recruited during this process.

The *MGF360-10L*- lacking mutant ASFV-Δ10L has been constructed from the virulent CN/GS/2018 strain. When injected intramuscularly with 10 HAD_50_ of this strain, 100% of the pigs survived the 19-day challenge with an increasing p30 antibody response. In addition, lower viremia and clinical signs were observed for this mutant [141].

#### 6.1.15. MGF360-11L

*MGF360-11L* has been found to reduce the IFN-β, ISG15, and ISG56 mRNA production mediated by cGAS, STING, TBK1, IKKε, IRF7, and IRF3-5D. Such inhibition is modulated by the 167–353-amino-acid residues of this gene. By interacting with TBK1 and IRF7, *MGF-360-11L* causes their degradation via the autophagic, ubiquitin–proteasome, and cysteine pathways. Furthermore, this gene prevents the phosphorylation of IRF3 and TBK1 brought on by the overexpression of cGAS-STING [122]. A vaccine is yet to be produced for this gene.

#### 6.1.16. MGF360-12L

*MGF360-12L* has been observed to inhibit the activation of the PRD (III-I) promoter. It also inhibits TBK1 and IRF3-5D’s exogenous expression. Additionally, *MGF360-12L* can prevent the activation of NF-κB due to the overexpression of cGAS-STING, TBK1, and IKKβ. By lowering the total protein level of IRF9, *MGF360-12L* blocks the activation of the ISRE promoter downstream of IFN-β signaling. Furthermore, the antiviral actions of IFN-β can be inhibited by the *MGF360-12L* protein [180,181]. Apart from the HLJ/18-6GD and HLJ/18-7GD recombinants, no other vaccine attempts exist for this gene at present [164].

#### 6.1.17. MGF360-13L

The cGAS-STING-mediated IFN-I pathway is substantially inhibited by the representative inhibitor *MGF360-13L*. It suppresses ISRE response and IFN-β activation brought on by molecules including TBK1, IRF3, and cGAS. The cGAS-STING-mediated IFN-β pathway is hampered by MGF360-13L’s interaction with TBK1 and its targeting for degradation. Further, the production of IL-1β also decreases *MGF360-13L* expression [127]. This gene has been deleted in the HLJ/18-6GD and HLJ/18-7GD mutants already mentioned [164].

#### 6.1.18. MGF360-14L

*MGF360-14L* enhances IRF3 degradation via ubiquitin-mediated proteolysis. It also suppresses the IFN-β promoter activity triggered by cGAS-STING signaling. Furthermore, via aiding the K63-linked ubiquitination of IRF3 by E3 ligase TRIM21, *MGF360-14L* binds with IRF3 and destabilizes it [182]. This gene is one of the deletions in the HLJ/18-6GD and the HLJ/18-7GD mutants [164].

#### 6.1.19. A276R or MGF 360-15R

*A276R* or *MGF 360-15R* has been observed to weaken the host cell IFN-I antiviral response. Correia et al. found that this gene can suppress IFN-β at a transcriptional and/or translational level via both the Poly(I:C)-stimulated TLR3 induction pathway and the IRF3-mediated cGAS-STING pathway. However, such suppression is not mediated by the IRF7 pathway. It should also be noted that such suppression is NF-κB-independent and “had no effect on the JAK-STAT pathway in response to either type I or type II IFN” [183].

The recombinant NH/P68ΔA276R produced from the attenuated NH/P68 strain showed no significant reduction in replication levels, both in vitro and in vivo, when compared to NH/P68. This mutant also failed against a heterologous challenge with the Armenia07 parental strain [153].

#### 6.1.20. MGF_300-2R

*MGF300-2R* uses the selective autophagy pathway to interact with and destroy IKKα and IKKβ. IKKα and IKKβ are polyubiquitinated in a K27-linked manner throughout by this gene. The ubiquitinated IKK proteins are recognized by the cargo receptor Tollip, which then facilitates their specific autophagic destruction. *MGF300-2R* promotes selective autophagic degradation of IKKβ by recruiting the E3 Ubiquitin Ligase TRIM21 [184,185].

*MGF300-2R* has been recognized as essential for viral replication in vitro. Further, the researchers tested the pathogenicity of the HLJ/18 strain lacking the *MGF300-2R* gene. This mutant, designated as Δ2R, is reported to be attenuated in vivo in a lower dose of 10^2^ TCID_50_. The inoculated pigs show lower viremia and higher levels of IL-1β and TNF-α, substantiating the gene’s virulence mechanism [185].

#### 6.1.21. MGF_300-4L

The *MGF 300-4L* gene, also known as J182L or L2HL, is one of the three MGF 300 genes (1L, 2R, and 4L) in the left variable region (LVR) of the genome [185,186,187]. This conserved gene has recently been described to suppress IL-1β and TNF-α levels, consequently suppressing NF-κB activation. To counter NF-κB activation, *MGF 300-4L* interacts with IKKβ and IκBα, targeting the former for lysosomal degradation via chaperone-mediated autophagy (CMA) and competitively preventing the β-TrCP-mediated ubiquitination destruction of the latter. MGF 300-4L has also been found to prevent the phosphorylation of IκBα and p65, interfering with the translocation of p65 to the nucleus.

MGF 300-4L has further been demonstrated by Wang and others to be essential for the virus. A significant reduction in replication kinetics has been observed both in vitro and in vivo for the deletion strain Del4L produced from the fatal HLJ/18. A heightened expression of immune signaling pathways was observed in PAMs infected with this deletion strain, such as NF-κB, TNF, MAPK, JAK-STAT, and PI3K-AKT. With regards to the pathogenicity, Del4L provided 50% protection to the inoculated pigs. In agreement with the in vitro findings, Del4L induced notably higher levels of IL-1β and TNF-α in vivo. The IFN-α levels for Del4L were similar to those of the parental strain in vitro, and lower than the HLJ/18-infected pigs [188].

#### 6.1.22. MGF-110-9L

While the virulence mechanism for *MGF110-9L* remains to be discussed, the mutant ASFV-Δ9L from the parental CN/GS/2018 displayed 5-to-10-fold lower growth kinetics in PAMs. This mutant saw low attenuation in pigs, with 2 out of the 5 experimental animals experiencing acute clinical signs and viremia. Transient moderate symptoms and viremia were observed for the remaining three pigs, with a progressively increasing p30 antibody response. IgG and IgM responses were also detected in the immunized animals [189].

#### 6.1.23. QP383R

*QP383R* has been reported as one of the genes to interfere with NLRP3 and AIM2-mediated inflammation [190]. *QP383R* was also found by Hao and others to influence the cGAS-STING-mediated IFN-I production through the suppression of steps upstream of STING, and the direct interaction with cGAS. Further investigation revealed a novel mechanism of inhibition, where *QP383R* could trigger a rise in the palmitoylation levels of cGAS, directly affecting its DNA-binding and enzymatic activity. Further, *QP383R* was also found to suppress dsDNA-induced downstream antiviral genes like *IFNB1*, *ISG54*, *CXCL10*, and *IL-6* [130].

Li et al. claimed that intramuscular inoculation of the single deletion ASFV-ΔQP383R mutant resulted in partial attenuation in the experimental pigs. The researchers also deleted this gene in conjunction with the *QP509L* gene from the virulent CN/GS/2018 strain. This double-deletion ASFV-ΔQP509L/QP383R mutant displayed a 10-to-80-fold reduction in viral titers in both in vitro and in vivo experiments. The immunized pigs also presented reduced levels of IFN-β, along with low anti-p30 antibody levels. However, this *QP383R*- and *QP509L*-deleted strain could not provide protection when challenged with the virulent parent strain [191].

### 6.2. Mediators of Apoptosis and Cell Autophagy

#### 6.2.1. A179L

*A179L* is known to code for an early-expressing anti-apoptosis protein homologous to the apoptosis regulator Bcl-2 through the BH1, BH2, and BH4 domains. It is seen to interact with “pro-apoptotic BCL-2 proteins (such as Bid, Bim, and Bad) with varying affinities, in addition to the autophagy-associated protein Beclin-1” [192,193]. This gene interferes with the activation of the pore-forming pro-apoptotic proteins Bax and Bak [193]. The association with Beclin-1 was also confirmed through the localization of pA179L with this protein in the mitochondria and ER. It has also been noted that A179L may bind to the BH3 motif of Beclin-1 [194,195]. Transfection experiments by Shi et al. demonstrated that the enzymatic activity of the apoptosis initiator caspase 8 was inhibited by *A179L* independently of its effects on BH3-containing pro-apoptotic proteins. This suggests that *A179L* acts on the extrinsic pathway of apoptosis, as caspase 8 is activated by external stimuli like TNF-α, Fas ligands, and Toll-like receptors (TLR). *A179L* can also enhance necroptosis triggered by TNF-α and the DNA virus HSV-1 [196].

A deletion experiment with *A179L* was carried out by Reis et al. involving the virulent isolate Benin 97/1, resulting in BeninΔA179L. This mutant had replication reduced by 10-fold in PAMs with a low MOI of 0.01, and showed higher apoptotic cells than infection with the parental strain at 16 hpi. It was highly attenuated in pigs even after a booster dose, but could not protect against a homologous challenge with Benin 97/1 [193].

#### 6.2.2. A224L

The *A224L* protein has been reported to bind with caspase-3′s proteolytic fragment, interfering with proapoptotic protein expression [107]. It also triggers NF-κB activation through cooperation with the TNF-R-associated protein TRAF-2. This stimulates IKK activity, as evidenced by the co-induction of ΙKKβ in cells transfected with this gene [197].

pA224L was confirmed to be a late-expressed anti-apoptotic protein by Nogal et al. in their deletion experiment involving the virulent Ba71V. Cells infected with the deletion mutant Ba71VΔIAP (ΔIAP) were seen to undergo apoptosis from 13 hpi. The mutant was also seen to induce caspase-3 processing at the early time of 13 hpi and significantly upregulate its expression from 15 hpi, suggesting that this gene prevents the infected cells in the late stages from dying [108]. Further, this mutant has been shown to induce the activation of translation initiation complex components like eIF4G1 and eIF4E [145]. Another deletion mutant, NH/P68ΔA224L, from the parental NH/P68, only produced mild clinical symptoms in pigs. This recombinant strain could protect against a homologous challenge with the related isolate L60, but failed against a heterologous Arm07 challenge [153].

#### 6.2.3. DP71L

*DP71L* (NL) has been described to regulate ER stress-mediated apoptosis. The protein encoded by this gene is similar to cellular GADD34 (growth arrest and DNA damage-inducible protein 34) and the Herpes simplex protein ICP34.5 as it can recruit PP1 (phosphase 1) to dephosphorylate the eIF2α protein (prevents the formation of the translation initiation complex). Phosphorylated eIF2α activates downstream proteins like ATF4 and CHOP, transcription factors for stress and pro-apoptotic protein, respectively. CHOP suppresses “the anti-apoptotic protein Beclin 2, depletes cellular glutathione and increases the production of reactive oxygen species, sensitizing the cell to ER stress and apoptosis”. The specific V^16^ and F^18^ residues in the PP1 binding site of pDP71L have been reported to be important. Further, the LSAVL motif between the 52–66-protein residues was identified as critical for eIF2α dephosphorylation and inhibition of CHOP nuclear localization [198].

A few deletion experiments have been carried out with NL. When deleted from the virulent E70 strain, the E70/43 recombinant had the same replication levels as the parental strain. However, this strain was attenuated in pigs except for a transitory fever and could protect against the parental E70 strain without any clinical symptoms or viremia presented [199]. This gene was also deleted from the attenuated OUR T88/3 strain in an attempt to attenuate it further. The new mutant OUR T88/3ΔDP2 did not show any noticeable reduction in growth kinetics when compared to the parent strain. Although this vaccine attempt had lower viral loads, it was declared futile since only 66% of the inoculated animals survived [162].

The mutant ASFV-G-ΔNL made by Ramirez-Medina and others from the ASFV-G isolate had a slight 10-fold decrease in replication kinetics at 96 hpi. In vivo, this strain saw moderate attenuation, with the pigs presenting delayed subclinical symptoms. Further, deletion of the NL gene from the ASFV-G-Δ9GL/ΔUK strain saw a 1000-fold reduction in replication levels compared to ASFV-G. This new ASFV-G-Δ9GL/ΔNL/ΔUK mutant was attenuated in pigs without any clinical symptoms, but could not protect against the parental ASFV-G [161]. Further, the combination deletion ASFV-GS-Δ18R/NL/UK, as discussed before, has been noted to be highly attenuated in pigs and can protect against the ASFV-G strain [22].

#### 6.2.4. EP153R

*EP153R* is reported to be involved in cell-to-cell adhesion and hemadsorption (HAD) through a cell attachment (RGD) sequence. It is a homolog of the N-terminal region of the CD44 glycoprotein (key role in adhesion and T-cell activation). It is also reported to inhibit apoptosis by controlling caspase-3 levels [200].

The deletion of this gene from the BA71V isolate (ΔEP153R) showed heightened levels of caspase-3 activation and an increase in cell death, confirming the gene’s anti-apoptotic effects [200]. The deletion mutant NH/P68ΔEP153R created by Gallardo and others from the attenuated NH/P68 did not have a noteworthy reduction in replication levels in vitro, and pigs immunized with this mutant only presented mild symptoms. This mutant could protect against a homologous L60 strain but provided no protection against the fatal Arm07 strain [153].

Petrovan and others deleted this gene singularly or in conjunction with *EP402R* (CD2v) from the attenuated strain BeninΔDP148R. The results showed that the lone deletion of *EP153R* from the mutant did not affect its multiplication rates in vitro, nor decrease its virulence or the persistence period in the blood in vivo. The strain BeninΔDP148RΔEP153RΔEP402R produced through the deletion of this gene with EP402R resulted in better attenuation, as no viremia was detected post-immunization. However, this combined deletion strain could only provide 75% protection to pigs after challenge with the parent strain Benin 97/1 [38].

Despite indicating that pEP153R has a synergistic action with CD2v, its deletion from the attenuated ASFV-G-Δ9GL strain proved counter-intuitive. This ASFV-G-Δ9GL/ΔCD2v/ΔEP153R mutant stripped the protective effects of this strain after a homologous challenge with ASFV-G, even though the growth kinetics and viremia rates post-immunization were lower than with ASFV-G-Δ9GL [201]. A similar result was found when this gene, in conjunction with CD2v, was deleted from the attenuated BA71∆CD2 mentioned. The BA71∆CD2EP153R mutant did not boost the efficiency of BA71∆CD2 [165].

#### 6.2.5. E199L

*E199L* (j18L) encodes for a late-expressing protein located in viral factories. Apart from viral entry and fusion, transfection experiments with *E199L* revealed that this gene is also associated with complete cell autophagy due to the formation of LC3 fluorescent spots, accumulation of LC3-II, and degradation of p62, all of which are indicators of autophagy. Additionally, the downregulation of pyrroline-5-carboxylate reductase-2 (PYCR2) transcription and translation was seen, confirming its role in the induction of autophagy [202,203]. In vitro experiments revealed that *E199L* had a significant induction of cell apoptosis by triggering the mitochondrial apoptotic pathway, which was apparent due to the mitochondrial apoptosis-related protein cyto C released from dysfunctional mitochondria into the cytoplasm, along with significant activation. Upon further investigation, *E199L* was found to competitively interfere with BAK (pro-apoptotic protein) and BCL-X_L_ (anti-apoptotic protein) interaction, ultimately disrupting the appropriate level of cell death and survival [204]. Deletion experiments with this gene are yet to be conducted.

#### 6.2.6. K205R

Located in viral factories, *K205R* is an early-expressed protein reported to be an ER stressor. Wang et al. (2022) found that this gene can activate the IRE1, PERK, and ATF6 ER stress signal pathways, and aid in ER stress-triggered autophagy by decreasing the expression of the selective autophagy receptor P62 (indicates autophagic flux) and the AKT/mTOR pathway (downregulates unc-51 like autophagy activating kinase 1). Following ER stress activating proinflammation, *K205R* could phosphorylate NF-κB subunits like IκBα and P65, and further upregulate the transcription of proinflammatory cytokines like *Il-6*, *Il-18*, and *Tnfa* [138]. A notable vaccine is yet to be attempted with this gene.

### 6.3. Mediators of Infection Cycle and Other Functions

#### 6.3.1. A240L

While its virulence mechanisms remain to be elaborated, this gene, which encodes for thymidine kinase, has been involved in a few deletion experiments. The deletion of this gene from the virulent Malawi LiL-20/1V and ASFV Haiti H811 strains by Moore et al. produced ASFV v5.3 and ASFV vH53, respectively. These mutants had diminished replication levels in macrophage cell lines. In vivo experimentation with the ASFV v5.3 mutant demonstrated attenuation in pigs and its ability to partially protect against a homologous challenge [205]. ASFV-G lacking this gene was reported to have minimized growth kinetics in cell cultures and did not cause any disease, even at high titers of inoculation into pigs. However, this mutant, referred to as ASFV-G/P-ΔTK, could not protect the pigs against the wild-type parental strain [206].

#### 6.3.2. B119L

*B119L* encodes for the highly conserved 9GL protein, which has been reported to be a late non-structural protein necessary for virion maturation [207]. It has been reported as a homolog of the flavin adenine dinucleotide (FAD) redox factor-dependent Erv1p/Alrp sulfhydryl oxidase family. In infected cells, oxidized pB119L contains a disulfide bond and interacts first with pA151R, which contains a CXXC redox motif, indicating that pA151R can participate in thiol–disulfide exchange reactions. pB119L, in turn, interacts with the late myristoylated pE248R protein. Since pE248R contains disulfide bridges and is related to the vaccinia virus redox pathway substrate L1R, it could be concluded that it is a substrate in pB119L’s redox pathway [208].

When this gene was deleted from the virulent Malawi Lil-20/1 isolate, up to 90–99% reduction in replication was seen in infected macrophages. Additionally, improper condensation and an undefined core shell were observed for the replicated virus, with nearly 90% of this Δ9GL mutant showing acentric nucleoids (ACNs). Δ9GL was substantially attenuated in pigs with only mild symptoms presented and a 100-to-1000-fold reduction in viremia. Further, no viremia or clinical signs were noticed after a homologous challenge with the parental strain in the groups that received high doses of the mutant (10^4^ or 10^6^ TCID_50_) [209]. The deletion of this gene was also studied for the Pr4 isolate, and the resulting attenuated Pr4Δ9GL mutant could protect all animals when challenged with the homologous parental strain without any clinical symptoms and a 10^6^-fold decrease in viremia levels [210].

The single deletion of B119L from ASFV-G displayed a 10-to-10,000-fold reduction in viral replication in PAMs. Pigs inoculated with low 10^2^ or 10^3^ HAD_50_ doses of this ASFV-G-Δ9GL mutant did not show clinical symptoms, but showed heterogenous viremia levels. Further, immunized pigs were also protected when exposed to the parental ASFV-G [211]. However, attempts at increasing the attenuation levels of this mutant by additional deletions of EP153R and CD2v, as described above, have proved ineffective [201]. The deletion of the UK gene from ASFV-G-Δ9GL, as mentioned before, saw the attenuation of this ASFV-G-Δ9GL/ΔUK mutant in pigs and could protect against the parental ASFV-G. [163] The HLJ/18-9GL&UK-del mutant, as mentioned before, was attenuated in pigs but had no protective effects against the parental strain [164].

CRISPR/Cas9 also has been employed to generate the deletion mutants 9GL1Δ and 9GL2Δ from the ASFV/Pol18/28298/O111 strain. These mutants did not display any difference in growth kinetics from the parent strain [156].

#### 6.3.3. DP148R

*DP148R* or *MGF 360-18R* is a non-essential, early-expressed gene during infection. While its virulence mechanism remains to be elucidated, Reis and others deleted this gene to produce the BeninΔDP148R strain from the virulent Benin 97/1 strain. This mutant caused only mild clinical signs and could successfully protect pigs when challenged with the parent strain. Additionally, the induction of IFN-γ-producing cells was observed in the immunized pigs [212]. However, the effects were opposite in the Georgia 2007/1 (ASFV-G) mutant Georgia∆DP148R, and all pigs had to be euthanized due to clinical signs similar to being infected with the parent strain [213]. Deletion of this gene from the virulent HLJ/18 (HLJ/18-DP148R-del) also failed to attenuate its virulence and proved fatal (Chen et al., 2020). When deleted in combination with other genes in the aforementioned ASFV-GS-Δ18R/NL/UK, this mutant could not only attenuate the virus, but also protect against the parent strain (Qi et al., 2023 [22]).

#### 6.3.4. H108R

The highly conserved H108R or J5R gene has been reported to encode a transmembrane protein in the inner envelope [214]. Among other virulence genes, this has been studied relatively less. It was involved by Vuono et al. in the production of a mutant ASFV-G-ΔH108R strain. This mutant is drastically attenuated in inoculated pigs and has stabilized replication levels. It could also provide absolute protection to a homologous challenge with ASFV-G, with a strong anti-ASFV antibody response [57].

#### 6.3.5. O61R

*O61R* is considered immunogenic, as 91.3% of pigs that survived in ASFV-affected Vietnamese farms had anti-p12 antibodies [215]. Duan and others have noted that p12 “allows membrane proteins on the cell surface to act as ASFV receptors”, indicating *O61R* is a major virulent gene [73].

### 6.4. Multifunctional Proteins

#### 6.4.1. A528R

A conserved member of the MGF multi-gene family, *A528R* or *MGF505-7R* is a gene that encodes a multifunctional protein. It inhibits TBK1 and IRF3 from phosphorylation. Additionally, specific regions of its encoded protein target the IFN-β promoter at the STING level. This protein has been observed to lead STING into autophagic degradation, as evidenced by the heightened levels of LC3II (autophagic marker protein) and ULK1 expression (autophagy initiation regulator protein) [121]. A528R can upregulate the expression of RNF125 (E3 ubiquitin ligase) and hinder Hes5 expression to degrade JAK1 and JAK2 of the IFN-γ- mediated JAK-STAT1 pathway [139].

This gene has also been identified to significantly reduce the IκB kinase component IKKε and IRF7 expression [216]. Further, an inclusive study by Liu et al. shows that pA528R targets the phosphorylation of the main signaling protein NF-κB p65 (RelA) of the TLR8 pathway, preventing its translocation to the nucleus and suppressing its promoter activity. An interaction between the predicted Ankyrin (ANK) regions of this gene and the Rel homology domain (RHD) of p65 was identified for the inhibition of p65 phosphorylation, ultimately affecting the antiviral innate immune response [217]. Consequently, A528R was found to suppress ISRE production and B-DNA stimulated downstream antiviral genes like “*Ifnb1*, *Tnfa*, *Il6*, and *Cxcl10*” [121].

A deletion experiment of this gene using the ASFV HLJ/18 strain (ASFV-Δ7R) showed incomplete attenuation in pigs and comparatively higher IL-1β and IFN-β levels. It was deduced that *MGF 505-7R* binds to NLRP3, disrupting the initiation of the inflammasome complex and preventing the maturation of IL-1β. Additionally, the higher IFN-β levels were attributed to the TLRs/NF-κB signaling pathway, as pMGF505-7R can interact with IKKα. This interaction reduces the levels of IFN-β as a result of inactive NF-κB [218]. When deleted in combination with *MGF 110-9L* from CN/GS/2018, this ASFV-Δ110-9L/505-7R strain had reduced replication rates compared to its parent strain, and the inoculated pigs only showed mild symptoms in high doses (10^5^ and 10^6^ HAD_50_) without severe viremia. It was observed that this mutant could protect against a challenge with CN/GS/2018 at high doses of 10^5^ and 10^6^ HAD_50_ without significant viremia and that two doses provided better protection. This protection has been associated with the anti-p30 antibody response observed [219]. Following the mechanism of *A528R*, this mutant showed suppression of the autophagy pathway with upregulated levels of TBK1, and the heightened expression of *Cxcl10*, *Isg56*, and *Isg15* [220].

#### 6.4.2. EP402R

The *EP402R* gene or 8-DR encodes the protein C2Dv, named due to its similarity to the host T-cell surface binding receptor cluster differentiation 2 protein involved in cell adhesion and signaling. This gene causes the signature rosette formation seen in RBCs around infected cells [221]. This can be explained by the immunoglobulin-like domains of CD2v, which have an affinity for specific receptors on the surface of RBCs, leading to hemadsorption (HAD) [222]. Studies have shown that HAD is dependent on the N-glycosylation of two Asp residues (N79 and N104) on the Nt domain of CD2v, both of which are critically dependent on a specific signal peptide [39]. CD2v has also been described to inhibit IFN-I production by interacting with STING’s transmembrane domain and hindering its translocation to the Golgi apparatus. Further, it can also interfere with the IFNAR1-TYK2 and IFNAR2-JAK1 interactions to inhibit ISG production [93]. CD2v can interact with actin-binding homolog SH3P7 (mAbp1 or HIP55), which is associated with “endocytosis, vesicle trafficking through the Golgi and signal transduction” [223]. Further, CD2v has also been reported to bind to the adaptor protein-1 (AP-1), disrupting its functions of protein transport to the endosomes from the trans-Golgi network (TGN) [224]. It has also been suggested that CD2v may play a role in the persistence of the virus in the host [38].

*EP402R* has been proven to be a non-essential gene as the replication levels of deletion mutants like Δ8-DR, BA71ΔCD2, ASFV-ΔEP402R, ASFV-G-Δ8DR, and ΔCongo-v_CD2v are not significantly reduced in vitro [93,225,226,227,228]. This gene has been involved in deletion experiments for different virulent strains. The mutant Δ8-DR constructed from the virulent Malawi Lil-20/1 strain showed delayed viremia in pigs but did not have a significantly different disease progression in comparison to the parental strain [225]. The most successful deletion vaccine is BA71ΔCD2 from the virulent BA71 (genotype I) strain. This mutant is highly attenuated in vivo, with undetectable clinical symptoms or viremia. This strain is not only able to protect the animals against a challenge with the homologous parental isolate, but also a challenge with the heterologous E75 (genotype I) isolate. Such cross-protection has been linked with the mutant triggering specific CD8^+^ T cells, which can recognize both the genotype I viruses. Further, this mutant was found to be capable of inhibiting disease caused by the genotype II Georgia 2007/1 strain, displaying protection across genotypes [227]. The deletion mutant ASFV-ΔEP402R presents a loss of HAD ability and induces higher levels of IFN-α, IFN-β, and ISG56 expression. This strain was also mildly weakened in pigs, with a 40% survival rate in comparison to the parental HLJ/18 strain [93].

Deletion attempts for this gene have also been made for ASFV-G. The mutant ASFV-G-Δ8DR by Borca et al., despite the reduced viremia levels, had no significant reduction in virulence in pigs [226]. Combination deletions have also been performed with this strain, and the mutants ASFV-G-Δ9GL/ΔCD2v and ASFV-G-Δ9GL/ΔCD2v/ΔEP153R were attenuated in vivo with significantly reduced viremia, but could not provide any protection against the parental ASFV-G. This indicates the varied influence of this gene in different isolates [201].

The vaccine from the ASFV-Kenya-1033 genotype IX strain has been attempted by Hemmink and others. This mutant was moderately attenuated, with the pigs showing reduced clinical symptoms and significant IFN-γ levels. The immunized pigs showed normal clinical conditions after a challenge with the parental strain [221]. When deleted in combination with *A238L* from ASFV-Kenya-1033, the ASFV-Ke-∆EP402R∆A238L mutant displayed attenuation in pigs, with only mild clinical scores and limited replication, but the attenuation was less than that with the single deletion mutant [155].

Strains from Congo have been involved in making *EP402R* deletion mutants. The mutant ΔCongoCD2v from the weak Congo-a strain lost its HAD properties, and no clinical signs or viremia were detected. However, this mutant did not confer protection against a virulent Congo-v challenge [229]. The mutant ΔCongo-v_CD2v from the lethal Congo-v strain had no attenuation in terms of disease progression in pigs but displayed delayed viremia [228].

Three mutants deleting this gene have been constructed by Chen and others from the virulent HLJ/18. The single deletion HLJ/18-CD2v-del and aforementioned HLJ/18-CD2v&UK-del mutant were both slightly attenuated in pigs. The HLJ/18-7GD strain, however, is reported to be highly attenuated in pigs and can protect against homologous challenge with the parental strain [164]. Two mutants have been produced by Petrovan et al. through the single or combination deletion of *EP402R* from BeninΔDP148R. Pigs immunized with these mutants resulted in shorter periods of viral persistence, supporting their theory as mentioned above. The BeninΔDP148RΔEP402R mutant presented with moderate clinical signs in the host but could completely protect pigs against the wild-type Benin 97/1 and had strong immune responses. The BeninΔDP148RΔEP153RΔEP402R strain was completely attenuated in pigs but could not protect against the virulent challenge [38]. The vaccine candidate ASFV-ΔECM3 has been obtained through the deletion of *EP402R* and four other genes (*EP153R*, *MGF_360-12L*, *MGF_360-13L*, and *MGF_360-14L*) from the virulent GZ201801. This mutant displayed successful attenuation in the experimental units, with no clinical signs detected. However, ASFV-ΔECM3 failed to protect the pigs against a challenge with the parental strain [230].

#### 6.4.3. S273R

S273R is a multifunctional protein, aiding virulence from different angles. pS273R has been found to inhibit IFN-β production mediated by the cGAS-STING pathway. By interacting with IRF3, pS273R interrupts TBK1 from phosphorylating IRF3 [231]. It also interferes with cGAS-STING by disrupting the association between IKKε and STING through IKKε de-sumoylation [62]. A study by Ma et al. revealed that this gene can degrade the host factor FoxJ1, dampening its immunostimulatory effects [232]. Additionally, *S273R* has been noted to negatively influence the JAK-STAT pathway, which regulates the expression of ISGs. pS273R forms a complex with STAT2 and recruits the E3 ubiquitin ligase DCST1, which catalyzes the attachment of K48-linked polyubiquitin chains to the K55 residue of STAT2 [233].

This gene has also been found to regulate pyroptosis, another programmed cell death mechanism, associated with the activation of inflammatory caspases in response to ligands like cytosolic viral DNA. The pyroptosis mediator gasdermin D (GSDMD) was found to be a novel target for proteolytic cleavage by pS273R. GSDMD is initially cleaved by caspase-1 at D279 (GSDMD-N_1–279)_ which triggers pyroptosis. This protein can cleave GSDMD at G107, and the resulting GSDMD-N_1–107_ does not induce pyroptosis in infected cells in vitro, which affects the production of inflammatory cytokines [61].

Li et al. found that pS273R further targets the production of cytoplasmic stress granules, which have been regarded as an “immune signaling platform in antiviral defense”. This inhibition happens due to pS273R cleaving its nucleating protein, G3BP1, at Gly140 [234]. The multi-suppression potential *S273R* exerts on the host’s immunity has significant importance, making it a good candidate for vaccine production.

## 7. Genes with Unknown Function

Although a majority of genes and the encoded proteins of ASFV are recorded, a significant portion of the ASFV genome comprises genes with unknown or least recorded functions, which are listed in Table 4. Despite their identification, the precise roles of these genes remain elusive. This represents a critical area of research in understanding the anatomy of the virus, and its mechanism of causing infection in the host. Some genes have only been reportedly used as targets in gene knockout experiments. In these experiments, the gene knockout resulted in a reduction of one or more key mechanisms of ASFV. This reduction suggests the potential role of the gene in that specific mechanism and thus in the pathogenicity of ASFV [235,236]. However, the understanding of the function of these genes remains limited, with no definitive conclusions drawn from existing research. Further exploration and in-depth studies are required to unravel the complexities of ASFV genes with unknown functions and their contributions to the virus’s pathogenesis. For instance, though the Multigene family (MGF) proteins are collectively involved in virulence transmission, their individual roles are still unknown.

## 8. Conclusions

Understanding the intricate mechanisms underlying African Swine Fever Virus (ASFV) infection is crucial for the development of effective control strategies. This review has highlighted several key aspects of ASFV, encompassing its infection cycle, structural features, immunomodulatory tactics, genes with unknown functions, and advancements in vaccine development.

Structurally, ASFV is a large, complex virus with a unique morphology. Its multilayered structure comprises an inner nucleoid core surrounded by a proteinaceous capsid, an inner and outer lipid envelope, and various structural proteins. Understanding the structural components of ASFV is essential for elucidating its interactions with host cells and the immune system to develop better counteractive approaches.

The infection cycle of ASFV involves a series of orchestrated events, beginning with viral entry into the host cell. The virus utilizes various strategies and protein-encoding genes that work individually or as a cluster in single or multiple mechanisms to penetrate the host cell membrane, uncoating to release its genetic material, and initiating the transcription, translation, and replication process and viral assembly and release. Despite extensive research into the replication process of ASFV, there remain gaps in understanding specific details of its mechanism, necessitating further investigation. The virulence genes of ASFV and their interactions with the host remain unclear. Therefore, conducting a more comprehensive analysis of ASFV genotypes through high-quality whole-genome sequencing is necessary to enhance our understanding of variation. This includes epidemiological tracing of the disease, exploring the evolutionary relationships and phenotypic differences among different ASFV strains, and investigating factors related to genomic variation [81].

Notably, the viral genome encodes numerous proteins involved in modulating the host cell machinery to promote the evasion of host immune responses. ASFV employs sophisticated immunomodulatory strategies to evade host immune surveillance and establish persistent infection. These include the inhibition of antiviral pathways and interference with antigen presentation mechanisms. By subverting host immune responses, ASFV can evade clearance and establish systemic infection, leading to severe disease manifestations in susceptible hosts.

Despite significant progress in understanding ASFV pathogenesis, a considerable portion of its genome remains uncharacterized, with many genes having unknown functions. Exploring the roles of these genes could provide valuable insights into viral replication, host interactions, and pathogenesis, ultimately facilitating the development of targeted therapeutic interventions.

Vaccine development against ASFV presents significant challenges due to the virus’s complex biology and genetic diversity. Traditional approaches such as live attenuated and inactivated vaccines have shown limited efficacy and safety concerns. Recent advances in vaccine technologies, including vectored vaccines, subunit vaccines, and gene editing strategies, offer promising avenues for the development of next-generation ASFV vaccines. However, the development of a safe and effective vaccine against ASFV remains a critical priority for global efforts to control the spread of this devastating disease.

Constant and accelerated research on ASFV is imperative, encompassing investigations into animal, human, and environmental factors. Comprehensive understanding of these factors and their contribution in virus transmission is essential for implementing targeted prevention and control measures. Similarly, emphasis on ASF pathological diagnosis and advancements in laboratory diagnostic technologies is critical for effective ASF management. ASF prevention and control challenges require concerted efforts and collaboration with the international community, and also necessitate collective action and shared expertise.

## Figures and Tables

**Table 1:** ASFV proteins involved in transcription and their functions.

**RNA polymerase subunits**		
pNP1450L (RP1)	viral transcription	[73]
pEP1242L (RP2)	viral transcription	[73]
pH359L (RP3)	viral transcription	[73]
pD205R (RP5)	viral transcription	[73]
pC147L (RP6)	viral transcription	[73]
pD339L (RP7)	Viral transcription	[73]
pC105R (RP9)	Viral transcription	[103]
pCP80R (RP10)	Viral transcription	[73]
**Transcription factors**		
pG1340L (VV A7L-like)	Transcription initiation	[103]
pD1133L (VV-D11L like)	Transcription initiation, ATPase activity, RNA modification	[103]
pB263R (TBP)	Transcription initiation, TATA-box binding	[103]
pB962L (I8-like)	Transcription initiation	[103]
pC315R (TFIIB)	RNAP recruitment to form transcription initiation complex	[103]
B385R (VV A2 like)	Transcription initiation	[103]
B175L (VV A1 like)	Transcription initiation, RNA modification	[104]
I243L (TFIIS)	elongation and release of arrested RNAP to initiate transcription from a different site	[103]
**Post-transcriptional modification**		
pD250R	5′ mRNA decapping enzyme	[104]
pC475L	3′ Poly(A) polymerase	[104]
NP868R	5′ end capping enzyme	[104]
pI215L	RNA modification—E2 ubiquitin binding enzyme	[104]
**Other transcription proteins**		
pA859L	Helicase protein	[104]
pM448R	RNA ligase for splicing	[104]
pB125R	Viral gene transcription and RNA modification	[81]
p15	Viral gene transcription	[68]
pQP509L (VV A18-like)	Transcription termination, transcript release	[42]

**Table 2:** ASFV proteins involved in translation and their functions.

Proteins	Functions
pI215L	Translation initiation—by overexpression of eIF4E through CUL4B binding
pE66L	Host translational arrest and facilitates ASFV translation
MGF 110-7L	enhances eIF2α phosphorylation and facilitates ASFV translation
pA224L	Inhibits apoptosis and induces expression of elongation initiation factors
pDP71L	dephosphorylates eIF2α and facilitates ASFV translation
pEP424R	post-translational RNA modification– capping enzyme—stabilize rRNA
pD250R	Reduce transcript count and prevent over translation
pI73R	Retention of cellular mRNAs within the nucleus

**Table 3:** ASFV proteins involved in replication and their functions.

Functions	Proteins
Primase	pC962R
DNA polymerase	pG1211R
Helicases	QP509L and Q706L
DNA-binding proteins	pA104R
DNA topoisomerase II	pP1192R
Import to nucleus	p10, p37 and p14
Import to cytoplasm	p37
Protein fused with DNA primase	pF1055L
Sliding clamp	pE301R
Deoxyribonucleotide triphosphate (dNTPs)	Thymidine kinase—pK196R Thymidylate kinase—pA240L Ribonucleotide reductase subunits—pF134L, pF778R Uracil deoxyribonucleoside triphosphatase dUTPase—pE165R
DNA ligase	pNP419L
Base-excision repair	Pol X—pO174L 5′/3′ exonuclease—pD345L AP endonuclease—pE296R

**Table 4:** ASFV genes with unknown or least recorded functions.

Gene	Function
*MGF_360-1L*	Unknown function
*MGF_360-3L*	Unknown function
*MGF_360-8L*	Unknown function
*MGF_360-10L*	Unknown function
*MGF_360-13L*	Unknown function
*MGF_360-16R*	Unknown function
*MGF_360-18R*	Unknown function
*MGF_360-19R*	Unknown function
*MGF_360-21R*	Unknown function
*MGF_300-1L*	Unknown function
*MGF_110-1L*	Unknown function
*MGF_110-2L*	Unknown function
*MGF_110-3L*	Unknown function
*MGF 110-4L*	Unknown function
*MGF_110-5L-6L*	Unknown function
*MGF_110-8L*	Unknown function
*MGF_110-10L—MGF_110-14L FUSION*	Unknown function
*MGF_110-11L*	Unknown function
*MGF_110-12L*	Unknown function
*MGF_110-13L*	Unknown function
*MGF_100-1R*	Unknown function
*MGF_100-1L*	Unknown function
*MGF_100-3L*	Unknown function
*MGF_505-1R*	Unknown function
*MGF_505-2R*	Unknown function
*MGF_505-4R*	Unknown function
*MGF_505-5R*	Unknown function
*MGF_505-6R*	Unknown function
*MGF_505-9R*	Unknown function
*MGF_505-10R*	Unknown function
*MGF_505-11L*	Unknown function
*ASFV_G_ACD_00090*	Non-expressed ORFs in HG2018 [237]
*ASFV_G_ACD_00120*	Unknown function
*ASFV_G_ACD_00160*	Non-expressed ORFs in GRG2007, HG2018 [237]
*ASFV_G_ACD_00190*	Non-expressed ORFs in HG2018 [237]
*ASFV_G_ACD_00210*	Non-expressed ORFs in HG2018 [237]
*ASFV_G_ACD_00240*	Unknown function
*ASFV_G_ACD_00300*	Unknown function
*ASFV_G_ACD_00320*	Unknown function
*ASFV_G_ACD_00330*	Unknown function
*ASFV_G_ACD_00350*	Unknown function
*ASFV_G_ACD_00360*	Non-expressed ORFs in GRG2007, HLJ2018, HG2018 [237]
*ASFV_G_ACD_00520*	Non-expressed ORFs in HG2018 [237]
*ASFV_G_ACD_00600*	Unknown function
*ASFV_G_ACD_01020*	Non-expressed ORFs in HLJ2018, HG2018 [237]
*ASFV_G_ACD_01760*	Non-expressed ORFs in GRG2007, HLJ2018 [237]
*ASFV_G_ACD_01870*	Non-expressed ORFs in HG2018 [237]
*ASFV_G_ACD_01940*	Unknown function
*ASFV_G_ACD_01960*	Unknown function
*ASFV_G_ACD_01980*	Unknown function
*ASFV_G_ACD_01990*	Non-expressed ORFs in GRG2007, HG2018 [237]
*285L*	May be relevant for replication or virulence in swine [236]
*A118R*	Unknown function
*B169L*	Unknown function
*B475L*	Unknown function
*B117L*	This viroporin, structured as a small membrane protein, aids ASFV infection by permeabilizing the ER-derived envelope. Activation happens in the low pH of the endosome during cell entry [238].
*B407L*	Unknown function
*B66L*	Unknown function
*CP123L*	Membrane proteins with single putative transmembrane segment. Unknown function [35]
*CP312R*	Unknown function
*C84L*	Deletion reduces virulence [235]
*C717R*	Belongs to a PK-like protein superfamily. Unknown function [239]
*C122R*	Codes a structural component of the virus particle [239]
*C257L*	Transmembrane region and putative signal peptide [81]
*C62L*	Unknown function
*DP60R*	Non-expressed ORFs in GRG2007, HG2018 [237]
*D129L*	Non-expressed ORFs in GRG2007 [237]
*D79L*	Unknown function
*DP79L*	Non-expressed ORFs in GRG2007 [237]
*DP238L*	Unknown function
*DP63R*	Non-expressed ORFs in GRG2007 [237]
*E423R*	Unknown function
*E146L*	Unknown function. Located in the transmembrane domain
*E111R*	Unknown function
*EP152R*	Unknown function. Located in the transmembrane domain
*F165R*	Unknown function
*H171R*	Unknown function
*H124R*	Unknown function
*H339R*	Alpha-NAC binding protein [35]
*H223R*	Non-expressed ORFs in HG2018 [237]
*I7L*	Confusion with I7L or L7L
*I8L*	Unknown function
*I9R*	Unknown function
*I10L*	Unknown function
*I196L*	Transmembrane region and putative signal peptide [81]
*K145R*	Late gene. May be involved in virion morphogenesis and immune evasion [236]
*K421R*	Unknown function
*KP177R*	Produces p22/p24. Unknown function
*L11L*	Unknown function
*L60L*	Could be involved in virulence and replication [240]
*R298L*	Unknown function
*X69R*	Non-expressed ORFs in HG2018 [237]

## Data Availability

Not applicable.

## Supplementary Materials

The following supporting information can be downloaded at: https://www.mdpi.com/article/10.3390/ani14152187/s1, Table S1. A list of all the genes of ASFV and their encoded proteins, their protein type, the process involved, and the functions performed by the respective genes/proteins in specific processes. Table S2. A list of the virulence genes of the genome categorized into Mediators of Immunomodulatory Pathways, Mediators of Apoptosis and Cell Autophagy, Mediators of Infection Cycle, and Other Functions and Multifunctional Proteins. Shown are their mechanism as virulence genes, the designed deletion mutants (based on previous literature), and year of deletion mutant. The resultant attenuation (No, moderate, high, complete) and the homologous/heterologous protection (No, partial, high, complete) are also tabulated.

## References

[1] Wang Y., Kang W., Yang W., Zhang J., Li D., Zheng H. (2021). Structure of African Swine Fever Virus and Associated Molecular Mechanisms Underlying Infection and Immunosuppression: A Review. Front. Immunol..

[2] Stancu A. (2019). ASF evolution and its economic impact in Europe over the past decade. USV Ann. Econ. Public Adm..

[3] Bastos A.D., Penrith M.-L., Cruciere C., Edrich J., Hutchings G., Roger F., Couacy-Hymann E., Thomson G.R. (2003). Genotyping field strains of African swine fever virus by partial p72 gene characterisation. Arch. Virol..

[4] Njau E.P., Machuka E.M., Cleaveland S., Shirima G.M., Kusiluka L.J., Okoth E.A., Pelle R. (2021). African swine fever virus (ASFV): Biology, genomics and genotypes circulating in sub-Saharan Africa. Viruses.

[5] Sauter-Louis C., Conraths F.J., Probst C., Blohm U., Schulz K., Sehl J., Fischer M., Forth J.H., Zani L., Depner K. (2021). African swine fever in wild boar in Europe—A review. Viruses.

[6] Dixon L., Islam M., Nash R., Reis A. (2019). African swine fever virus evasion of host defences. Virus Res..

[7] Couacy-Hymann E. (2019). African swine fever in sub-Saharan African countries. Transbound. Anim. Dis. Sahel. Afr. Connect. Reg..

[8] Fauquet C.M., Mayo M.A., Maniloff J., Desselberger U., Ball L.A. (2005). Virus Taxonomy: VIIIth Report of the International Committee on Taxonomy of Viruses.

[9] Frant M., Lyjak M., Bocian L., Barszcz A., Niemczuk K., Wozniakowski G. (2020). African swine fever virus (ASFV) in Poland: Prevalence in a wild boar population (2017–2018). Vet. Med..

[10] Pavone S., Iscaro C., Dettori A., Feliziani F. (2023). African swine fever: The state of the art in Italy. Animals.

[11] Wu K., Liu J., Wang L., Fan S., Li Z., Li Y., Yi L., Ding H., Zhao M., Chen J. (2020). Current state of global African swine fever vaccine development under the prevalence and transmission of ASF in China. Vaccines.

[12] Lu G., Pan J., Zhang G. (2020). African swine fever virus in Asia: Its rapid spread and potential threat to unaffected countries. J. Infect..

[13] Zhang Y.Y., Zhang J.Y., Yang J.J., Yang J.M., Han X., Mi L.J. (2023). Identification of a natural variant of African swine fever virus in China. Chin. J. Vet. Sci..

[14] Kim G., Park J.E., Kim S.J., Kim Y., Kim W., Kim Y.K., Jheong W. (2022). Complete genome analysis of the African swine fever virus isolated from a wild boar responsible for the first viral outbreak in Korea, 2019. Front. Vet. Sci..

[15] Mighell E., Ward M.P. (2021). African Swine Fever spread across Asia, 2018–2019. Transbound. Emerg. Dis..

[16] Senthilkumar D., Rajukumar K., Venkatesh G., Singh F., Tosh C., Kombiah S., Dubey C.K., Chakravarty A., Barman N.N., Singh V.P. (2022). Complete genome analysis of African swine fever virus isolated from domestic pigs during the first ASF outbreaks in India. Transbound. Emerg. Dis..

[17] Le V.P., Ahn M.J., Kim J.S., Jung M.C., Yoon S.W., Trinh T.B.N., Le T.N., Kim H.K., Kang J.A., Lim J.W. (2023). A Whole-Genome Analysis of the African Swine Fever Virus That Circulated during the First Outbreak in Vietnam in 2019 and Subsequently in 2022. Viruses.

[18] World Organization for Animal Health (WOAH) African Swine Fever Situation Report. https://wahis.woah.org/#/in-review/4236?reportId=158536&fromPage=event-dashboard-url.

[19] https://wahis.woah.org/#/event-management.

[20] Zhang Y., Wang Q., Zhu Z., Wang S., Tu S., Zhang Y., Zou Y., Liu Y., Liu C., Ren W. (2023). Tracing the Origin of Genotype II African Swine Fever Virus in China by Genomic Epidemiology Analysis. Transbound. Emerg. Dis..

[21] Sanchez E.G., Perez-Nunez D., Revilla Y. (2019). Development of vaccines against African swine fever virus. Virus Res..

[22] Qi X., Feng T., Ma Z., Zheng L., Liu H., Shi Z., Shen C., Li P., Wu P., Ru Y. (2023). Deletion of DP148R, DP71L, and DP96R attenuates African swine fever virus, and the mutant strain confers complete protection against homologous challenges in pigs. J. Virol..

[23] Juszkiewicz M., Walczak M., Woźniakowski G., Podgórska K. (2023). African swine fever: Transmission, spread, and control through biosecurity and disinfection, including polish trends. Viruses.

[24] Liu Y., Xie Z., Li Y., Song Y., Di D., Liu J., Gong L., Chen Z., Wu J., Ye Z. (2023). Evaluation of an I177L gene-based five-gene-deleted African swine fever virus as a live attenuated vaccine in pigs. Emerg. Microbes Infect..

[25] Hsu C.-H., Chang C.-Y., Otake S., Molitor T.W., Perez A. (2024). Strategies for Transboundary Swine Disease Management in Asian Islands: Foot and Mouth Disease, Classical Swine Fever, and African Swine Fever in Taiwan, Japan, and the Philippines. Vet. Sci..

[26] Dixon L.K., Chapman D.A., Netherton C.L., Upton C. (2013). African swine fever virus replication and genomics. Virus Res..

[27] Urbano A.C., Ferreira F. (2022). African swine fever control and prevention: An update on vaccine development. Emerg. Microbes Infect..

[28] Zhao D., Liu R., Zhang X., Li F., Wang J., Zhang J., Liu X., Wang L., Zhang J., Wu X. (2019). Replication and virulence in pigs of the first African swine fever virus isolated in China. Emerg. Microbes Infect..

[29] Medrano M. (2023). A Literature Review to Gather the Scientific Evidence for an African Swine Fever virus (ASFV) Exposure Assessment of US Domestic Pigs Raised in Total Confinement and/or with Outdoor Access to ASFV-Infected Feral Swine. https://conservancy.umn.edu/items/4c8f4b8a-c992-4d4f-a911-53096ccabf77.

[30] Malogolovkin A., Kolbasov D. (2019). Genetic and antigenic diversity of African swine fever virus. Virus Res..

[31] Nefedeva M., Titov I., Tsybanov S., Malogolovkin A. (2020). Recombination shapes African swine fever virus serotype-specific locus evolution. Sci. Rep..

[32] Zhao D., Sun E., Huang L., Ding L., Zhu Y., Zhang J., Shen D., Zhang X., Zhang Z., Ren T. (2023). Highly lethal genotype I and II recombinant African swine fever viruses detected in pigs. Nat. Commun..

[33] Lacasta A., Monteagudo P.L., Jimenez-Marin A., Accensi F., Ballester M., Argilaguet J., Galindo-Cardiel I., Segales J., Salas M.L., Dominguez J. (2015). Live attenuated African swine fever viruses as ideal tools to dissect the mechanisms involved in viral pathogenesis and immune protection. Vet. Res..

[34] Giammarioli M., Alessandro D., Cammà C., Masoero L., Torresi C., Marcacci M., Zoppi S., Curini V., Rinaldi A., Rossi E. (2023). Molecular characterization of the first African swine fever virus genotype II strains identified from mainland Italy, 2022. Pathogens.

[35] Alejo A.M.T., Guerra M., Andrés G. (2018). A proteomic atlas of the African swine fever virus particle. J. Virol..

[36] Jia N., Ou Y., Pejsak Z., Zhang Y., Zhang J. (2017). Roles of African Swine Fever Virus Structural Proteins in Viral Infection. J. Vet. Res..

[37] Rodríguez J.M.Y.R., Almazán F.E., Viñuela E.L., Rodríguez J.F. (1993). African swine fever virus encodes a CD2 homolog responsible for the adhesion of erythrocytes to infected cells. Virol. J..

[38] Zhang M., Lv L., Luo H., Cai H., Yu L., Jiang Y., Gao F., Tong W., Li L., Li G. (2023). The CD2v protein of African swine fever virus inhibits macrophage migration and inflammatory cytokines expression by downregulating EGR1 expression through dampening ERK1/2 activity. Vet. Res..

[39] Pérez-Núñez D., García-Belmonte R., Riera E., Fernández-Sesma M.H., Vigara-Astillero G., Revilla Y. (2023). Signal peptide and N-glycosylation of N-terminal-CD2v determine the hemadsorption of African swine fever virus. J. Virol..

[40] Vuono E.A., Ramirez-Medina E., Pruitt S., Rai A., Espinoza N., Velazquez-Salinas L., Gladue D.P., Borca M.V. (2021). Evaluation of the function of the ASFV KP177R gene, encoding for structural protein p22, in the process of virus replication and in swine virulence. Viruses.

[41] Liu S., Luo Y., Wang Y., Li S., Zhao Z., Bi Y., Sun J., Peng R., Song H., Zhu D. (2019). Cryo-EM Structure of the African Swine Fever Virus. Cell Host Microbe.

[42] Yang S., Miao C., Liu W., Zhang G., Shao J., Chang H. (2023). Structure and function of African swine fever virus proteins: Current understanding. Front. Microbiol..

[43] Chang Z., Du Y., Li R., Sun X., Chen Y., Li M., Fan L., Liu S., Wang S., Ding P. (2023). Development and characterization of monoclonal antibody against the critical loop structure of african swine fever virus P72 protein. Vet. Microbiol..

[44] Munoz A.L., Tabares E. (2022). Characteristics of the major structural proteins of African swine fever virus: Role as antigens in the induction of neutralizing antibodies. A review. Virology.

[45] Liu Q., Ma B., Qian N., Zhang F., Tan X., Lei J., Xiang Y. (2019). Structure of the African swine fever virus major capsid protein p72. Cell Res..

[46] Zhou P.L.L., Zhang K., Wang B., Tang L., Li M., Wang T., Sun Y., Li S., Qiu H.J. (2022). Deletion of the H240R Gene of African Swine Fever Virus decreases infectious progeny virus production due to aberrant virion morphogenesis and enhances inflammatory cytokine expression in porcine macrophages. Virol. J..

[47] Huang L.L.H., Ye G., Liu X., Chen W., Wang Z., Zhao D., Zhang Z., Feng C., Hu L., Yu H. (2023). Deletion of African swine fever virus (ASFV) H240R gene attenuates the virulence of ASFV by enhancing NLRP3-mediated inflammatory responses. Virol. J..

[48] Cui S., Wang Y., Gao X., Xin T., Wang X., Yu H., Chen S., Jiang Y., Chen Q., Jiang F. (2022). African swine fever virus M1249L protein antagonizes type I interferon production via suppressing phosphorylation of TBK1 and degrading IRF3. Virus Res..

[49] Zheng W., Xia N., Zhang J., Cao Q., Jiang S., Luo J., Wang H., Chen N., Zhang Q., Meurens F. (2022). African Swine Fever Virus Structural Protein p17 Inhibits cGAS-STING Signaling Pathway Through Interacting with STING. Front. Immunol..

[50] Suarez C., Gutierrez-Berzal J., Andres G., Salas M.L., Rodriguez J.M. (2010). African swine fever virus protein p17 is essential for the progression of viral membrane precursors toward icosahedral intermediates. J. Virol..

[51] Aicher S.M., Monaghan P., Netherton C.L., Hawes P.C. (2021). Unpicking the Secrets of African Swine Fever Viral Replication Sites. Viruses.

[52] Galindo I., Vinuela E., Carrascosa A.L. (2000). Characterization of the african swine fever virus protein p49: A new late structural polypeptide. J. Gen. Virol..

[53] Anggy F.P., Nugroho W.S., Irianingsih S.H., Enny S., Srihanto E.A. (2023). Genetic analysis of African swine fever viruses based on E183L (p54) gene, circulating in South Sumatra and Lampung province, Indonesia. Vet. World.

[54] Rodriguez J.M., Garcia-Escudero R., Salas M.L., Andres G. (2004). African swine fever virus structural protein p54 is essential for the recruitment of envelope precursors to assembly sites. J. Virol..

[55] Rodriguez I., Nogal M.L., Redrejo-Rodriguez M., Bustos M.J., Salas M.L. (2009). The African swine fever virus virion membrane protein pE248R is required for virus infectivity and an early postentry event. J. Virol..

[56] Li L., Du N., Chen J., Zhang K., Tong W., Zheng H., Zhao R., Tong G., Gao F. (2022). Establishment and Application of a Quantitative PCR Method for E248R Gene of African Swine Fever Virus. Vet. Sci..

[57] Vuono E.R.-M.E., Silva E., Rai A., Pruitt S., Espinoza N., Valladares A., Velazquez-Salinas L., Gladue D.P., Borca M.V. (2022). Deletion of the H108R gene reduces virulence of the pandemic Eurasia strain of African swine fever virus with surviving animals being protected against virulent challenge. Virol. J..

[58] Matamoros T., Alejo A., Rodriguez J.M., Hernaez B., Guerra M., Fraile-Ramos A., Andres G. (2020). African Swine Fever Virus Protein pE199L Mediates Virus Entry by Enabling Membrane Fusion and Core Penetration. mBio.

[59] Andrés G.S.-M.C., Vinuela E. (1997). Assembly of African swine fever virus: Role of polyprotein pp220. Virol. J..

[60] Li G., Fu D., Zhang G., Zhao D., Li M., Geng X., Sun D., Wang Y., Chen C., Jiao P. (2020). Crystal structure of the African swine fever virus structural protein p35 reveals its role for core shell assembly. Protein Cell.

[61] Zhao G., Li T., Liu X., Zhang T., Zhang Z., Kang L., Song J., Zhou S., Chen X., Wang X. (2022). African swine fever virus cysteine protease pS273R inhibits pyroptosis by noncanonically cleaving gasdermin D. J. Biol. Chem..

[62] Luo J., Zhang J., Ni J., Jiang S., Xia N., Guo Y., Shao Q., Cao Q., Zheng W., Chen N. (2022). The African swine fever virus protease pS273R inhibits DNA sensing cGAS-STING pathway by targeting IKKepsilon. Virulence.

[63] Li H.L.Q., Shao L., Xiang Y. (2023). Structural Insights into the Assembly of the African Swine Fever Virus Inner Capsid. Virol. J..

[64] Liu K., Meng Y., Chai Y., Li L., Sun H., Gao G.F., Tan S., Qi J. (2021). Crystal structure of the African swine fever virus core shell protein p15. Biosaf. Health.

[65] Eulalio A., Nunes-Correia I., Salas J., Salas M.L., Simoes S., Pedroso de Lima M.C. (2007). African swine fever virus p37 structural protein is localized in nuclear foci containing the viral DNA at early post-infection times. Virus Res..

[66] Zhang X., Guan X., Wang Q., Wang X., Yang X., Li S., Zhao X.T., Yuan M., Liu X., Qiu H.J. (2023). Identification of the p34 Protein of African Swine Fever Virus as a Novel Viral Antigen with Protection Potential. Viruses.

[67] Tian Y., Liang C., Zhou J., Sun F., Liu Y., Chen Y., Zhu X., Liu H., Ding P., Liu E. (2023). Identification of a novel B-cell epitope of the African swine fever virus p34 protein and development of an indirect ELISA for the detection of serum antibodies. Front. Microbiol..

[68] Fu D., Zhao D., Zhang W., Zhang G., Li M., Zhang Z., Wang Y., Sun D., Jiao P., Chen C. (2020). Structure of African swine fever virus p15 reveals its dual role for membrane-association and DNA binding. Protein Cell.

[69] Guo F., Shi Y., Yang M., Guo Y., Shen Z., Li M., Chen Y., Liang R., Yang Y., Chen H. (2021). The structural basis of African swine fever virus core shell protein p15 binding to DNA. FASEB J..

[70] Frouco G., Freitas F.B., Coelho J., Leitao A., Martins C., Ferreira F. (2017). DNA-Binding Properties of African Swine Fever Virus pA104R, a Histone-Like Protein Involved in Viral Replication and Transcription. J. Virol..

[71] Freitas F.B., Simoes M., Frouco G., Martins C., Ferreira F. (2019). Towards the Generation of an ASFV-pA104R DISC Mutant and a Complementary Cell Line-A Potential Methodology for the Production of a Vaccine Candidate. Vaccines.

[72] Urbano A.C., Ferreira F. (2020). Role of the DNA-Binding Protein pA104R in ASFV Genome Packaging and as a Novel Target for Vaccine and Drug Development. Vaccines.

[73] Duan X., Ru Y., Yang W., Ren J., Hao R., Qin X., Li D., Zheng H. (2022). Research progress on the proteins involved in African swine fever virus infection and replication. Front. Immunol..

[74] Chen Q., Li L., Guo S., Liu Z., Liu L., Tan C., Chen H., Wang X. (2023). African swine fever virus pA104R protein acts as a suppressor of type I interferon signaling. Front. Microbiol..

[75] Istrate C., Marques J., Bule P., Correia S., Aires-da-Silva F., Duarte M., Reis A.L., Machuqueiro M., Leitao A., Victor B.L. (2022). In Silico Characterization of African Swine Fever Virus Nucleoprotein p10 Interaction with DNA. Viruses.

[76] Nunes-Correia I., Rodriguez J.M., Eulalio A., Carvalho A.L., Citovsky V., Simoes S., Faro C., Salas M.L., Pedroso de Lima M.C. (2008). African swine fever virus p10 protein exhibits nuclear import capacity and accumulates in the nucleus during viral infection. Vet. Microbiol..

[77] Sánchez E.G., Quintas A., Pérez-Núñez D., Nogal M., Barroso S., Carrascosa Á.L., Revilla Y. (2012). African swine fever virus uses macropinocytosis to enter host cells. PLoS Pathog..

[78] Alonso C., Galindo I., Cuesta-Geijo M.A., Cabezas M., Hernaez B., Munoz-Moreno R. (2013). African swine fever virus-cell interactions: From virus entry to cell survival. Virus Res..

[79] Karger A., Perez-Nunez D., Urquiza J., Hinojar P., Alonso C., Freitas F.B., Revilla Y., Le Potier M.F., Montoya M. (2019). An Update on African Swine Fever Virology. Viruses.

[80] Sanchez E.G., Perez-Nunez D., Revilla Y. (2017). Mechanisms of Entry and Endosomal Pathway of African Swine Fever Virus. Vaccines.

[81] Li Z., Chen W., Qiu Z., Li Y., Fan J., Wu K., Li X., Zhao M., Ding H., Fan S. (2022). African Swine Fever Virus: A Review. Life.

[82] Franzoni G., Dei Giudici S., Oggiano A. (2018). Infection, modulation and responses of antigen-presenting cells to African swine fever viruses. Virus Res..

[83] Guo X., Zhang M., Liu X., Zhang Y., Wang C., Guo Y. (2023). Attachment, Entry, and Intracellular Trafficking of Classical Swine Fever Virus. Viruses.

[84] Zhang K., Li S., Liu S., Li S., Qu L., Gao G.F., Qiu H.-J. (2021). Spatiotemporally Orchestrated Interactions between Viral and Cellular Proteins Involved in the Entry of African Swine Fever Virus. Viruses.

[85] Mettlen M., Chen P.H., Srinivasan S., Danuser G., Schmid S.L. (2018). Regulation of Clathrin-Mediated Endocytosis. Annu. Rev. Biochem..

[86] Revilla Y., Perez-Nunez D., Richt J.A. (2018). African Swine Fever Virus Biology and Vaccine Approaches. Adv. Virus Res..

[87] Sobhy H. (2017). A comparative review of viral entry and attachment during large and giant dsDNA virus infections. Arch. Virol..

[88] Mercer J.H.A. (2009). Virus entry by macropinocytosis. Nat. Cell Biol..

[89] Alonso C., Miskin J., Hernáez B., Fernandez-Zapatero P., Soto L., Cantó C., Rodríguez-Crespo I., Dixon L., Escribano J.M. (2001). African swine fever virus protein p54 interacts with the microtubular motor complex through direct binding to light-chain dynein. J. Virol..

[90] Chen X., Chen X., Liang Y., Xu S., Weng Z., Gao Q., Huang Z., Zhang G., Gong L. (2022). Interaction network of African swine fever virus structural protein p30 with host proteins. Front. Microbiol..

[91] Gómez-Puertas P., Rodríguez F., Oviedo J.M., Brun A., Alonso C., Escribano J.M. (1998). The African Swine Fever Virus Proteins p54 and p30 Are Involved in Two Distinct Steps of Virus Attachment and Both Contribute to the Antibody-Mediated Protective Immune Response. Virology.

[92] Chen X., Zheng J., Liu C., Li T., Wang X., Li X., Bao M., Li J., Huang L., Zhang Z. (2023). CD1d facilitates African swine fever virus entry into the host cells via clathrin-mediated endocytosis. Emerg. Microbes Infect..

[93] Huang L., Chen W., Liu H., Xue M., Dong S., Liu X., Feng C., Cao S., Ye G., Zhou Q. (2023). African Swine Fever Virus HLJ/18 CD2v Suppresses Type I IFN Production and IFN-Stimulated Genes Expression through Negatively Regulating cGMP-AMP Synthase–STING and IFN Signaling Pathways. J. Immunol..

[94] Alcamí A., Angulo A., López-Otín C., Muñoz M., Freije J.M., Carrascosa A.L., Viñuela E. (1992). Amino acid sequence and structural properties of protein p12, an African swine fever virus attachment protein. J. Virol..

[95] Galindo I., Alonso C. (2017). African Swine Fever Virus: A Review. Viruses.

[96] Hernáez B., Guerra M., Salas M.L., Andrés G. (2016). African swine fever virus undergoes outer envelope disruption, capsid disassembly and inner envelope fusion before core release from multivesicular endosomes. PLoS Pathog..

[97] Andrés G. (2017). African swine fever virus gets undressed: New insights on the entry pathway. J. Virol..

[98] Urquiza J., Cuesta-Geijo M.Á., García-Dorival I., Fernández Ó., del Puerto A., Díaz J.F., Alonso C. (2024). Identification of a Potential Entry-Fusion Complex Based on Sequence Homology of African Swine Fever and Vaccinia Virus. Viruses.

[99] García-Dorival I., Cuesta-Geijo M.Á., Galindo I., Del Puerto A., Barrado-Gil L., Urquiza J., Alonso C. (2023). Elucidation of the Cellular Interactome of African Swine Fever Virus Fusion Proteins and Identification of Potential Therapeutic Targets. Viruses.

[100] Kao S., Kao C.-F., Chang W., Ku C. (2023). Widespread distribution and evolution of poxviral entry-fusion complex proteins in giant viruses. Microbiol. Spectr..

[101] Gaudreault N.N., Madden D.W., Wilson W.C., Trujillo J.D., Richt J.A. (2020). African Swine Fever Virus: An Emerging DNA Arbovirus. Front. Vet. Sci..

[102] Urbano A., Forth J., Olesen A., Dixon L., Rasmussen T., Cackett G., Werner F., Karger A., Andrés G., Wang X. (2021). African swine fever virus: Cellular and molecular aspects. Understanding and Combatting African Swine Fever: A European Perspective.

[103] Cackett G., Sýkora M., Werner F. (2020). Transcriptome view of a killer: African swine fever virus. Biochem. Soc. Trans..

[104] Rodríguez J.M., Salas M.L. (2013). African swine fever virus transcription. Virus Res..

[105] Chen S., Feng C., Fang Y., Zhou X., Xu L., Wang W., Kong X., Maikel P.P., Pan Q., Yin Y. (2019). The Eukaryotic Translation Initiation Factor 4F Complex Restricts Rotavirus Infection via Regulating the Expression of IRF1 and IRF7. Int. J. Mol. Sci..

[106] Barrado-Gil L., Del Puerto A., Muñoz-Moreno R., Galindo I., Cuesta-Geijo M., Urquiza J., Nistal-Villán E., Maluquer de Motes C., Alonso C. (2020). African Swine Fever Virus Ubiquitin-Conjugating Enzyme Interacts with Host Translation Machinery to Regulate the Host Protein Synthesis. Front. Microbiol..

[107] Dolata K.M., Pei G., Netherton C.L., Karger A. (2023). Functional Landscape of African Swine Fever Virus–Host and Virus–Virus Protein Interactions. Viruses.

[108] Nogal M.A.L., González de Buitrago G., Rodríguez C., Cubelos B., Carrascosa A.L., Salas M.a.L., Revilla Y. (2001). African swine fever virus IAP homologue inhibits caspase activation and promotes cell survival in mammalian cells. J. Virol..

[109] Zhong H., Fan S., Du Y., Zhang Y., Zhang A., Jiang D., Han S., Wan B., Zhang G. (2022). African swine fever virus MGF110-7L induces host cell translation suppression and stress granule formation by activating the PERK/PKR-eIF2α pathway. Microbiol. Spectr..

[110] Eulalio A., Nunes-Correia I., Carvalho A., Faro C., Citovsky V., Simoes S., Pedroso de Lima M. (2004). Two African swine fever virus proteins derived from a common precursor exhibit different nucleocytoplasmic transport activities. J. Virol..

[111] Freitas F.B., Frouco G., Martins C., Ferreira F. (2019). The QP509L and Q706L superfamily II RNA helicases of African swine fever virus are required for viral replication, having non-redundant activities. Emerg. Microbes Infect..

[112] Rathakrishnan A., Connell S., Petrovan V., Moffat K., Goatley L.C., Jabbar T., Sánchez-Cordón P.J., Reis A.L., Dixon L.K. (2022). Differential effect of deleting members of African swine fever virus multigene families 360 and 505 from the genotype II Georgia 2007/1 isolate on virus replication, virulence, and induction of protection. J. Virol..

[113] Muñoz-Moreno R., Barrado-Gil L., Galindo I., Alonso C. (2015). Analysis of HDAC6 and BAG3-aggresome pathways in African swine fever viral factory formation. Viruses.

[114] Alejo A., García-Castey M., Guerra M., Hernáez B., Martín V., Matamoros T., Andrés G. (2023). African swine fever virus transmembrane protein pEP84R guides core assembly. PLoS Pathog..

[115] Netherton C., Rouiller I., Wileman T. (2004). The subcellular distribution of multigene family 110 proteins of African swine fever virus is determined by differences in C-terminal KDEL endoplasmic reticulum retention motifs. J. Virol..

[116] Gao Q., Yang Y., Feng Y., Quan W., Luo Y., Wang H., Zheng J., Chen X., Huang Z., Chen X. (2022). Effects of the NF-κB Signaling Pathway Inhibitor BAY11-7082 in the Replication of ASFV. Viruses.

[117] Zheng X., Nie S., Feng W.H. (2022). Regulation of antiviral immune response by African swine fever virus (ASFV). Virol. Sin..

[118] García-Belmonte R., Pérez-Núñez D., Revilla Y. (2022). Controlling the cGAS-STING pathway: The signature of ASFV virulence. J. Immunol. Sci..

[119] García-Belmonte R., Pérez-Núñez D., Pittau M., Richt J.A., Revilla Y. (2019). African Swine Fever Virus Armenia/07 Virulent Strain Controls Interferon Beta Production through the cGAS-STING Pathway. J. Virol..

[120] Dodantenna N., Ranathunga L., Chathuranga W.G., Weerawardhana A., Cha J.-W., Subasinghe A., Gamage N., Haluwana D., Kim Y., Jheong W. (2022). African swine fever virus EP364R and C129R target cyclic GMP-AMP to inhibit the cGAS-STING signaling pathway. J. Virol..

[121] Li D., Yang W., Li L., Li P., Ma Z., Zhang J., Qi X., Ren J., Ru Y., Niu Q. (2021). African swine fever virus MGF-505-7R negatively regulates cGAS–STING-mediated signaling pathway. J. Immunol..

[122] Yang K., Xue Y., Niu H., Shi C., Cheng M., Wang J., Zou B., Wang J., Niu T., Bao M. (2022). African swine fever virus MGF360-11L negatively regulates cGAS-STING-mediated inhibition of type I interferon production. Vet. Res..

[123] Liu X., Liu H., Ye G., Xue M., Yu H., Feng C., Zhou Q., Liu X., Zhang L., Jiao S. (2022). African swine fever virus pE301R negatively regulates cGAS-STING signaling pathway by inhibiting the nuclear translocation of IRF3. Vet. Microbiol..

[124] Huang L., Xu W., Liu H., Xue M., Liu X., Zhang K., Hu L., Li J., Liu X., Xiang Z. (2021). African swine fever virus pI215L negatively regulates cGAS-STING signaling pathway through recruiting RNF138 to inhibit K63-linked ubiquitination of TBK1. J. Immunol..

[125] He W.-R., Yuan J., Ma Y.-H., Zhao C.-Y., Yang Z.-Y., Zhang Y., Han S., Wan B., Zhang G.-P. (2022). Modulation of host antiviral innate immunity by African swine fever virus: A review. Animals.

[126] Cheng M., Luo J., Duan Y., Yang Y., Shi C., Sun Y., Lu Y., Wang J., Li X., Wang J. (2022). African swine fever virus MGF505-3R inhibits cGAS-STING-mediated IFN-β pathway activation by degrading TBK1. Anim. Dis..

[127] Luo J., Cheng M., Duan Y., Xing X., Lu M., Sun Y., Shi C., Wang J., Lu Y., Li X. (2023). African swine fever virus encoded protein MGF360-13L inhibits cGAS-STING-mediated IFN-I signaling pathway. Gene.

[128] Cheng M., Kanyema M.M., Sun Y., Zhao W., Lu Y., Wang J., Li X., Shi C., Wang J., Wang N. (2023). African swine fever virus L83L negatively regulates the cGAS-STING-mediated IFN-I pathway by recruiting tollip to promote STING autophagic degradation. J. Virol..

[129] Yu L., Zhu Z., Deng J., Tian K., Li X. (2023). Antagonisms of ASFV towards host defense mechanisms: Knowledge gaps in viral immune evasion and pathogenesis. Viruses.

[130] Hao S., Zheng X., Zhu Y., Yao Y., Li S., Xu Y., Feng W.-h. (2023). African swine fever virus QP383R dampens type I interferon production by promoting cGAS palmitoylation. Front. Immunol..

[131] Sunwoo S.Y., Garcia-Belmonte R., Walczak M., Vigara-Astillero G., Kim D.M., Szymankiewicz K., Kochanowski M., Liu L., Tark D., Podgorska K. (2024). Deletion of MGF505-2R Gene Activates the cGAS-STING Pathway Leading to Attenuation and Protection against Virulent African Swine Fever Virus. Vaccines.

[132] Portugal R., Leitão A., Martins C. (2018). Modulation of type I interferon signaling by African swine fever virus (ASFV) of different virulence L60 and NHV in macrophage host cells. Vet. Microbiol..

[133] Sun S.-C. (2011). Non-canonical NF-κB signaling pathway. Cell Res..

[134] Miskin J.E., Abrams C.C., Dixon L.K. (2000). African swine fever virus protein A238L interacts with the cellular phosphatase calcineurin via a binding domain similar to that of NFAT. J. Virol..

[135] Wang X., Wu J., Wu Y., Chen H., Zhang S., Li J., Xin T., Jia H., Hou S., Jiang Y. (2018). Inhibition of cGAS-STING-TBK1 signaling pathway by DP96R of ASFV China 2018/1. Biochem. Biophys. Res. Commun..

[136] Correia S., Moura P.L., Ventura S., Leitão A., Parkhouse R.M.E. (2023). I329L: A dual action viral antagonist of TLR activation encoded by the African swine fever virus (ASFV). Viruses.

[137] Hong J., Chi X., Yuan X., Wen F., Rai K.R., Wu L., Song Z., Wang S., Guo G., Chen J.-L. (2022). I226R protein of African swine fever virus is a suppressor of innate antiviral responses. Viruses.

[138] Wang Q., Zhou L., Wang J., Su D., Li D., Du Y., Yang G., Zhang G., Chu B. (2022). African swine fever virus K205r induces ER stress and consequently activates autophagy and the NF-κB signaling pathway. Viruses.

[139] Li D., Zhang J., Yang W., Li P., Ru Y., Kang W., Li L., Ran Y., Zheng H. (2021). African swine fever virus protein MGF-505-7R promotes virulence and pathogenesis by inhibiting JAK1-and JAK2-mediated signaling. J. Biol. Chem..

[140] Zhang K., Yang B., Shen C., Zhang T., Hao Y., Zhang D., Liu H., Shi X., Li G., Yang J. (2022). MGF360-9L is a major virulence factor associated with the African swine fever virus by antagonizing the JAK/STAT signaling pathway. MBio.

[141] Li D., Peng J., Wu J., Yi J., Wu P., Qi X., Ren J., Peng G., Duan X., Ru Y. (2023). African swine fever virus MGF-360-10L is a novel and crucial virulence factor that mediates ubiquitination and degradation of JAK1 by recruiting the E3 ubiquitin ligase HERC5. Mbio.

[142] Riera E., García-Belmonte R., Madrid R., Pérez-Núñez D., Revilla Y. (2023). African swine fever virus ubiquitin-conjugating enzyme pI215L inhibits IFN-I signaling pathway through STAT2 degradation. Front. Microbiol..

[143] Ye G., Zhang Z., Liu X., Liu H., Chen W., Feng C., Li J., Zhou Q., Zhao D., Zhang S. (2024). African swine fever virus pH240R enhances viral replication via inhibition of the type I IFN signaling pathway. J. Virol..

[144] Gao Q., Yang Y., Luo Y., Chen X., Gong T., Wu D., Feng Y., Zheng X., Wang H., Zhang G. (2023). African swine fever virus envelope glycoprotein CD2v interacts with host CSF2RA to regulate the JAK2-STAT3 pathway and inhibit apoptosis to facilitate virus replication. J. Virol..

[145] Sánchez E.G., Quintas A., Nogal M., Castelló A., Revilla Y. (2013). African swine fever virus controls the host transcription and cellular machinery of protein synthesis. Virus Res..

[146] Keita D., Heath L., Albina E. (2010). Control of African swine fever virus replication by small interfering RNA targeting the A151R and VP72 genes. Antivir. Ther..

[147] Li Y., Huang L., Li H., Zhu Y., Yu Z., Zheng X., Weng C., Feng W.-h. (2024). ASFV pA151R negatively regulates type I IFN production via degrading E3 ligase TRAF6. Front. Immunol..

[148] Ramirez-Medina E., Vuono E., Pruitt S., Rai A., Espinoza N., Valladares A., Spinard E., Silva E., Velazquez-Salinas L., Gladue D.P. (2022). ASFV gene A151R is involved in the process of virulence in domestic swine. Viruses.

[149] Granja A.G., Nogal M.L., Hurtado C., Del Aguila C., Carrascosa A.L., Salas M.L., Fresno M., Revilla Y. (2006). The viral protein A238L inhibits TNF-α expression through a CBP/p300 transcriptional coactivators pathway. J. Immunol..

[150] Granja A.G., Nogal M.L., Hurtado C., Vila V., Carrascosa A.L., Salas M.L., Fresno M., Revilla Y. (2004). The viral protein A238L inhibits cyclooxygenase-2 expression through a nuclear factor of activated T cell-dependent transactivation pathway. J. Biol. Chem..

[151] Neilan J., Lu Z., Kutish G., Zsak L., Lewis T., Rock D. (1997). A Conserved African Swine Fever Virus IκB Homolog, 5EL, Is Nonessential for Growthin Vitroand Virulence in Domestic Swine. Virology.

[152] Salguero F., Gil S., Revilla Y., Gallardo C., Arias M., Martins C. (2008). Cytokine mRNA expression and pathological findings in pigs inoculated with African swine fever virus (E-70) deleted on A238L. Vet. Immunol. Immunopathol..

[153] Gallardo C., Sánchez E.G., Pérez-Núñez D., Nogal M., de León P., Carrascosa Á.L., Nieto R., Soler A., Arias M.L., Revilla Y. (2018). African swine fever virus (ASFV) protection mediated by NH/P68 and NH/P68 recombinant live-attenuated viruses. Vaccine.

[154] Pérez-Núñez D., Sunwoo S.-Y., García-Belmonte R., Kim C., Vigara-Astillero G., Riera E., Kim D.-m., Jeong J., Tark D., Ko Y.-S. (2022). Recombinant African swine fever virus Arm/07/CBM/c2 lacking CD2v and A238L is attenuated and protects pigs against virulent Korean Paju strain. Vaccines.

[155] Abkallo H.M., Hemmink J.D., Oduor B., Khazalwa E.M., Svitek N., Assad-Garcia N., Khayumbi J., Fuchs W., Vashee S., Steinaa L. (2022). Co-deletion of A238L and EP402R genes from a genotype IX African swine fever virus results in partial attenuation and protection in swine. Viruses.

[156] Woźniakowski G., Mazur-Panasiuk N., Walczak M., Juszkiewicz M., Frant M., Niemczuk K. (2020). Attempts at the development of a recombinant African swine fever virus strain with abrogated, and gene structure using the CRISPR/Cas9 system. J. Vet. Res..

[157] Abkallo H.M., Svitek N., Oduor B., Awino E., Henson S.P., Oyola S.O., Mwalimu S., Assad-Garcia N., Fuchs W., Vashee S. (2021). Rapid CRISPR/Cas9 editing of genotype IX African swine fever virus circulating in eastern and central Africa. Front. Genet..

[158] Swine D. (1998). A Nonessential African Swine Fever Virus Gene. J. Virol..

[159] Dodantenna N., Cha J.-W., Chathuranga K., Chathuranga W.G., Weerawardhana A., Ranathunga L., Kim Y., Jheong W., Lee J.-S. (2024). The African Swine Fever Virus Virulence Determinant DP96R Suppresses Type I IFN Production Targeting IRF3. Int. J. Mol. Sci..

[160] Zsak L., Caler E., Lu Z., Kutish G., Neilan J., Rock D. (1998). A nonessential African swine fever virus gene UK is a significant virulence determinant in domestic swine. J. Virol..

[161] Ramirez-Medina E., Vuono E., O’Donnell V., Holinka L.G., Silva E., Rai A., Pruitt S., Carrillo C., Gladue D.P., Borca M.V. (2019). Differential effect of the deletion of African swine fever virus virulence-associated genes in the induction of attenuation of the highly virulent Georgia strain. Viruses.

[162] Abrams C.C., Goatley L., Fishbourne E., Chapman D., Cooke L., Oura C.A., Netherton C.L., Takamatsu H.-H., Dixon L.K. (2013). Deletion of virulence associated genes from attenuated African swine fever virus isolate OUR T88/3 decreases its ability to protect against challenge with virulent virus. Virology.

[163] O‘Donnell V., Risatti G.R., Holinka L.G., Krug P.W., Carlson J., Velazquez-Salinas L., Azzinaro P.A., Gladue D.P., Borca M.V. (2017). Simultaneous deletion of the 9GL and UK genes from the African swine fever virus Georgia 2007 isolate offers increased safety and protection against homologous challenge. J. Virol..

[164] Chen W., Zhao D., He X., Liu R., Wang Z., Zhang X., Li F., Shan D., Chen H., Zhang J. (2020). A seven-gene-deleted African swine fever virus is safe and effective as a live attenuated vaccine in pigs. Sci. China Life Sci..

[165] Lopez E., Bosch-Camós L., Ramirez-Medina E., Vuono E., Navas M.J., Muñoz M., Accensi F., Zhang J., Alonso U., Argilaguet J. (2021). Deletion mutants of the attenuated recombinant ASF virus, BA71ΔCD2, show decreased vaccine efficacy. Viruses.

[166] Andrés G.N., García-Escudero R.N., Viñuela E., Salas M.A.L., Rodríguez J.M. (2001). African swine fever virus structural protein pE120R is essential for virus transport from assembly sites to plasma membrane but not for infectivity. J. Virol..

[167] Cui S., Wang Y., Chen S., Fang L., Jiang Y., Pang Z., Jiang Y., Guo X., Zhu H., Jia H. (2023). African swine fever virus E120R inhibited cGAS-STING-mediated IFN-β and NF-κB pathways. Anim. Res. One Health.

[168] Liu H., Zhu Z., Feng T., Ma Z., Xue Q., Wu P., Li P., Li S., Yang F., Cao W. (2021). African swine fever virus E120R protein inhibits interferon beta production by interacting with IRF3 to block its activation. J. Virol..

[169] Zhang Y., Ke J., Zhang J., Yang J., Yue H., Zhou X., Qi Y., Zhu R., Miao F., Li Q. (2021). African swine fever virus bearing an I226R gene deletion elicits robust immunity in pigs to African swine fever. J. Virol..

[170] Ran Y., Li D., Xiong M.-G., Liu H.-N., Feng T., Shi Z.-W., Li Y.-H., Wu H.-N., Wang S.-Y., Zheng H.-X. (2022). African swine fever virus I267L acts as an important virulence factor by inhibiting RNA polymerase III-RIG-I-mediated innate immunity. PLoS Pathog..

[171] Deng Y., Wang Y., Li L., Miao E.A., Liu P. (2022). Post-translational modifications of proteins in cytosolic nucleic acid sensing signaling pathways. Front. Immunol..

[172] Wen Y., Duan X., Ren J., Zhang J., Guan G., Ru Y., Li D., Zheng H. (2024). African Swine Fever Virus I267L Is a Hemorrhage-Related Gene Based on Transcriptome Analysis. Microorganisms.

[173] Zhang Y., Ke J., Zhang J., Yue H., Chen T., Li Q., Zhou X., Qi Y., Zhu R., Wang S. (2021). I267L is neither the virulence-nor the replication-related gene of African swine fever virus and its deletant is an ideal fluorescent-tagged virulence strain. Viruses.

[174] Reis A.L., Goatley L.C., Jabbar T., Lopez E., Rathakrishnan A., Dixon L.K. (2020). Deletion of the gene for the type I interferon inhibitor I329L from the attenuated African swine fever virus OURT88/3 strain reduces protection induced in pigs. Vaccines.

[175] Sun L., Miao Y., Wang Z., Chen H., Dong P., Zhang H., Wu L., Jiang M., Chen L., Yang W. (2022). Structural insight into African swine fever virus I73R protein reveals it as a Z-DNA binding protein. Transbound. Emerg. Dis..

[176] Liu Y., Shen Z., Xie Z., Song Y., Li Y., Liang R., Gong L., Di D., Liu J., Liu J. (2023). African swine fever virus I73R is a critical virulence-related gene: A potential target for attenuation. Proc. Natl. Acad. Sci. USA.

[177] Lai D.C., Chaudhari J., Vu H.L. (2024). African swine fever virus early protein pI73R suppresses the type-I IFN promoter activities. Virus Res..

[178] Borca M.V., O’Donnell V., Holinka L.G., Ramírez-Medina E., Clark B.A., Vuono E.A., Berggren K., Alfano M., Carey L.B., Richt J.A. (2018). The L83L ORF of African swine fever virus strain Georgia encodes for a non-essential gene that interacts with the host protein IL-1β. Virus Res..

[179] Yang K., Huang Q., Wang R., Zeng Y., Cheng M., Xue Y., Shi C., Ye L., Yang W., Jiang Y. (2021). African swine fever virus MGF505-11R inhibits type I interferon production by negatively regulating the cGAS-STING-mediated signaling pathway. Vet. Microbiol..

[180] Chen Q., Wang X., Jiang S., Gao X., Huang S., Liang Y., Jia H., Zhu H. (2023). MGF360-12L of ASFV-SY18 is an immune-evasion protein that inhibits host type I IFN, NF-κB, and JAK/STAT pathways. Pol. J. Vet. Sci..

[181] Zhuo Y., Guo Z., Ba T., Zhang C., He L., Zeng C., Dai H. (2021). African Swine Fever Virus MGF360-12L Inhibits Type I Interferon Production by Blocking the Interaction of Importin alpha and NF-kappaB Signaling Pathway. Virol. Sin..

[182] Wang Y., Cui S., Xin T., Wang X., Yu H., Chen S., Jiang Y., Gao X., Jiang Y., Guo X. (2021). African Swine Fever Virus MGF360-14L Negatively Regulates Type I Interferon Signaling by Targeting IRF3. Front. Cell Infect. Microbiol..

[183] Correia S., Ventura S., Parkhouse R.M. (2013). Identification and utility of innate immune system evasion mechanisms of ASFV. Virus Res..

[184] Lu Z., Luo R., Lan J., Chen S., Qiu H.-J., Wang T., Sun Y. (2024). The MGF300-2R Protein of African Swine Fever Virus Promotes IKKβ Ubiquitination by Recruiting the E3 Ubiquitin Ligase TRIM21. Viruses.

[185] Wang T., Luo R., Zhang J., Lu Z., Li L.-F., Zheng Y.-H., Pan L., Lan J., Zhai H., Huang S. (2023). The MGF300-2R protein of African swine fever virus is associated with viral pathogenicity by promoting the autophagic degradation of IKK α and IKK β through the recruitment of TOLLIP. PLoS Pathog..

[186] Chapman D.A., Tcherepanov V., Upton C., Dixon L.K. (2008). Comparison of the genome sequences of non-pathogenic and pathogenic African swine fever virus isolates. J. Gen. Virol..

[187] Yozawa T., Kutish G., Afonso C., Lu Z., Rock D. (1994). Two novel multigene families, 530 and 300, in the terminal variable regions of African swine fever virus genome. Virology.

[188] Wang T., Luo R., Zhang J., Lan J., Lu Z., Zhai H., Li L.-F., Sun Y., Qiu H.-J. (2024). The African swine fever virus MGF300-4L protein is associated with viral pathogenicity by promoting the autophagic degradation of IKK β and increasing the stability of I κ B α. Emerg. Microbes Infect..

[189] Li D., Liu Y., Qi X., Wen Y., Li P., Ma Z., Liu Y., Zheng H., Liu Z. (2021). African swine fever virus MGF-110-9L-deficient mutant has attenuated virulence in pigs. Virol. Sin..

[190] Song J., Li K., Li T., Zhao G., Zhou S., Li H., Li J., Weng C. (2020). Screening of PRRSV-and ASFV-encoded proteins involved in the inflammatory response using a porcine iGLuc reporter. J. Virol. Methods.

[191] Li D., Wu P., Liu H., Feng T., Yang W., Ru Y., Li P., Qi X., Shi Z., Zheng H. (2022). A QP509L/QP383R-deleted African swine fever virus is highly attenuated in swine but does not confer protection against parental virus challenge. J. Virol..

[192] Cackett G., Matelska D., Sýkora M., Portugal R., Malecki M., Bähler J., Dixon L., Werner F. (2020). The African swine fever virus transcriptome. J. Virol..

[193] Reis A.L., Rathakrishnan A., Goulding L.V., Barber C., Goatley L.C., Dixon L.K. (2023). Deletion of the gene for the African swine fever virus BCL-2 family member A179L increases virus uptake and apoptosis but decreases virus spread in macrophages and reduces virulence in pigs. J. Virol..

[194] Hernaez B., Cabezas M., Munoz-Moreno R., Galindo I., Cuesta-Geijo M.A., Alonso C. (2013). A179L, a new viral Bcl2 homolog targeting Beclin 1 autophagy related protein. Curr. Mol. Med..

[195] Banjara S., Shimmon G.L., Dixon L.K., Netherton C.L., Hinds M.G., Kvansakul M. (2019). Crystal structure of African swine fever virus A179L with the autophagy regulator Beclin. Viruses.

[196] Shi J., Liu W., Zhang M., Sun J., Xu X. (2021). The A179L gene of African swine fever virus suppresses virus-induced apoptosis but enhances necroptosis. Viruses.

[197] Rodríguez C.I., Nogal M.A.L., Carrascosa A.L., Salas M.A.L., Fresno M., Revilla Y. (2002). African swine fever virus IAP-like protein induces the activation of nuclear factor kappa B. J. Virol..

[198] Barber C., Netherton C., Goatley L., Moon A., Goodbourn S., Dixon L. (2017). Identification of residues within the African swine fever virus DP71L protein required for dephosphorylation of translation initiation factor eIF2α and inhibiting activation of pro-apoptotic CHOP. Virology.

[199] Zsak L., Lu Z., Kutish G., Neilan J., Rock D. (1996). An African swine fever virus virulence-associated gene NL-S with similarity to the herpes simplex virus ICP34. 5 gene. J. Virol..

[200] Hurtado C., Granja A.G., Bustos M.J., Nogal M.L., de Buitrago G.G., de Yébenes V.G., Salas M.L., Revilla Y., Carrascosa A.L. (2004). The C-type lectin homologue gene (EP153R) of African swine fever virus inhibits apoptosis both in virus infection and in heterologous expression. Virology.

[201] Gladue D.P., O’Donnell V., Ramirez-Medina E., Rai A., Pruitt S., Vuono E.A., Silva E., Velazquez-Salinas L., Borca M.V. (2020). Deletion of CD2-like (CD2v) and C-type lectin-like (EP153R) genes from African swine fever virus Georgia-∆ 9GL abrogates its effectiveness as an experimental vaccine. Viruses.

[202] Chen S., Zhang X., Nie Y., Li H., Chen W., Lin W., Chen F., Xie Q. (2021). African swine fever virus protein E199L promotes cell autophagy through the interaction of PYCR2. Virol. Sin..

[203] Ou R., Zhang X., Cai J., Shao X., Lv M., Qiu W., Xuan X., Liu J., Li Z., Xu Y. (2016). Downregulation of pyrroline-5-carboxylate reductase-2 induces the autophagy of melanoma cells via AMPK/mTOR pathway. Tumor Biol..

[204] Li T., Zhao G., Zhang T., Zhang Z., Chen X., Song J., Wang X., Li J., Huang L., Wen L. (2021). African swine fever virus pE199L induces mitochondrial-dependent apoptosis. Viruses.

[205] Moore D., Zsak L., Neilan J., Lu Z., Rock D. (1998). The African swine fever virus thymidine kinase gene is required for efficient replication in swine macrophages and for virulence in swine. J. Virol..

[206] Sanford B., Holinka L., O‘donnell V., Krug P., Carlson J., Alfano M., Carrillo C., Wu P., Lowe A., Risatti G. (2016). Deletion of the thymidine kinase gene induces complete attenuation of the Georgia isolate of African swine fever virus. Virus Res..

[207] Geng X.-M., Xi Y.-M., Huang X.-M., Wang Y.-L., Wang X.-Y., Ouyang K., Chen Y., Wei Z.-Z., Qin Y.-F., Huang W.-J. (2024). Construction of and evaluation of the immune response to two recombinant pseudorabies viruses expressing the B119L and EP364R proteins of African swine fever virus. Arch. Virol..

[208] Rodríguez I., Redrejo-Rodríguez M., Rodrίguez J.M., Alejo A., Salas J., Salas M.a.L. (2006). African swine fever virus pB119L protein is a flavin adenine dinucleotide-linked sulfhydryl oxidase. J. Virol..

[209] Lewis T., Zsak L., Burrage T., Lu Z., Kutish G., Neilan J., Rock D. (2000). An African swine fever virus ERV1-ALR homologue, 9GL, affects virion maturation and viral growth in macrophages and viral virulence in swine. J. Virol..

[210] Neilan J.G., Zsak L., Lu Z., Burrage T.G., Kutish G.F., Rock D.L. (2004). Neutralizing antibodies to African swine fever virus proteins p30, p54, and p72 are not sufficient for antibody-mediated protection. Virology.

[211] O‘Donnell V., Holinka L.G., Krug P.W., Gladue D.P., Carlson J., Sanford B., Alfano M., Kramer E., Lu Z., Arzt J. (2015). African swine fever virus Georgia 2007 with a deletion of virulence-associated gene 9GL (B119L), when administered at low doses, leads to virus attenuation in swine and induces an effective protection against homologous challenge. J. Virol..

[212] Reis A.L., Goatley L.C., Jabbar T., Sanchez-Cordon P.J., Netherton C.L., Chapman D.A., Dixon L.K. (2017). Deletion of the African swine fever virus gene DP148R does not reduce virus replication in culture but reduces virus virulence in pigs and induces high levels of protection against challenge. J. Virol..

[213] Rathakrishnan A., Reis A.L., Goatley L.C., Moffat K., Dixon L.K. (2021). Deletion of the K145R and DP148R genes from the virulent ASFV Georgia 2007/1 isolate delays the onset, but does not reduce severity, of clinical signs in infected pigs. Viruses.

[214] Bao Y.J., Qiu J., Luo Y., Rodríguez F., Qiu H.J. (2021). The genetic variation landscape of African swine fever virus reveals frequent positive selection and adaptive flexibility. Transbound. Emerg. Dis..

[215] Luong H.Q., Lai H.T., Do L.D., Ha B.X., Nguyen G.V., Vu H.L. (2022). Differential antibody responses in sows and finishing pigs naturally infected with African swine fever virus under field conditions. Virus Res..

[216] Yang K., Xue Y., Niu T., Li X., Cheng M., Bao M., Zou B., Shi C., Wang J., Yang W. (2022). African swine fever virus MGF505-7R protein interacted with IRF7and TBK1 to inhibit type I interferon production. Virus Res..

[217] Liu X., Ao D., Jiang S., Xia N., Xu Y., Shao Q., Luo J., Wang H., Zheng W., Chen N. (2021). African swine fever virus A528R inhibits TLR8 mediated NF-κB activity by targeting p65 activation and nuclear translocation. Viruses.

[218] Li J., Song J., Kang L., Huang L., Zhou S., Hu L., Zheng J., Li C., Zhang X., He X. (2021). pMGF505-7R determines pathogenicity of African swine fever virus infection by inhibiting IL-1β and type I IFN production. PLoS Pathog..

[219] Ding M., Dang W., Liu H., Zhang K., Xu F., Tian H., Huang H., Shi Z., Sunkang Y., Qin X. (2022). Sequential deletions of interferon inhibitors MGF110-9L and MGF505-7R result in sterile immunity against the Eurasia strain of Africa swine fever. J. Virol..

[220] Zhu G., Ren J., Li D., Ru Y., Qin X., Feng T., Tian H., Lu B., Shi D., Shi Z. (2023). Combinational deletions of MGF110-9L and MGF505-7R genes from the African swine fever virus inhibit TBK1 degradation by an autophagy activator PIK3C2B to promote type I interferon production. J. Virol..

[221] Hemmink J.D., Khazalwa E.M., Abkallo H.M., Oduor B., Khayumbi J., Svitek N., Henson S.P., Blome S., Keil G., Bishop R.P. (2022). Deletion of the CD2v gene from the genome of ASFV-Kenya-IX-1033 partially reduces virulence and induces protection in pigs. Viruses.

[222] Liu S., Ding P., Du Y., Ren D., Chen Y., Li M., Sun X., Wang S., Chang Z., Li R. (2022). Development and characterization of monoclonal antibodies against the extracellular domain of African swine fever virus structural protein, CD2v. Front. Microbiol..

[223] Kay-Jackson P.C., Goatley L.C., Cox L., Miskin J., Parkhouse R., Wienands J., Dixon L.K. (2004). The CD2v protein of African swine fever virus interacts with the actin-binding adaptor protein SH3P7. J. Gen. Virol..

[224] Pérez-Núñez D., García-Urdiales E., Martinez-Bonet M., Nogal M.L., Barroso S., Revilla Y., Madrid R. (2015). CD2v interacts with adaptor protein AP-1 during African swine fever infection. PLoS ONE.

[225] Borca M., Carrillo C., Zsak L., Laegreid W., Kutish G., Neilan J., Burrage T., Rock D. (1998). Deletion of a CD2-like gene, 8-DR, from African swine fever virus affects viral infection in domestic swine. J. Virol..

[226] Borca M.V., o’Donnell V., Holinka L.G., Risatti G.R., Ramirez-Medina E., Vuono E.A., Shi J., Pruitt S., Rai A., Silva E. (2020). Deletion of CD2-like gene from the genome of African swine fever virus strain Georgia does not attenuate virulence in swine. Sci. Rep..

[227] Monteagudo P.L., Lacasta A., López E., Bosch L., Collado J., Pina-Pedrero S., Correa-Fiz F., Accensi F., Navas M.J., Vidal E. (2017). BA71ΔCD2: A new recombinant live attenuated African swine fever virus with cross-protective capabilities. J. Virol..

[228] Koltsov A., Krutko S., Kholod N., Sukher M., Belov S., Korotin A., Koltsova G. (2023). Deletion of the CD2 gene in the virulent ASFV Congo strain affects Viremia in domestic swine, but not the virulence. Animals.

[229] Koltsova G., Koltsov A., Krutko S., Kholod N., Tulman E.R., Kolbasov D. (2021). Growth kinetics and protective efficacy of attenuated ASFV strain Congo with deletion of the EP402 gene. Viruses.

[230] Xie Z., Liu Y., Di D., Liu J., Gong L., Chen Z., Li Y., Yu W., Lv L., Zhong Q. (2022). Protection evaluation of a five-gene-deleted African swine fever virus vaccine candidate against homologous challenge. Front. Microbiol..

[231] Li H., Zheng X., Li Y., Zhu Y., Xu Y., Yu Z., Feng W.-H. (2023). African swine fever virus S273R protein antagonizes type I interferon production by interfering with TBK1 and IRF3 interaction. Virol. Sin..

[232] Ma C., Li S., Yang F., Cao W., Liu H., Feng T., Zhang K., Zhu Z., Liu X., Hu Y. (2022). FoxJ1 inhibits African swine fever virus replication and viral S273R protein decreases the expression of FoxJ1 to impair its antiviral effect. Virol. Sin..

[233] Li Y.-H., Peng J.-L., Xu Z.-S., Xiong M.-G., Wu H.-N., Wang S.-Y., Li D., Zhu G.-Q., Ran Y., Wang Y.-Y. (2023). African swine fever virus cysteine protease ps273R inhibits type I interferon signaling by mediating STAT2 degradation. J. Virol..

[234] Li T., Li X., Wang X., Chen X., Zhao G., Liu C., Bao M., Song J., Li J., Huang L. (2023). African swine fever virus pS273R antagonizes stress granule formation by cleaving the nucleating protein G3BP1 to facilitate viral replication. J. Biol. Chem..

[235] Yang J., Zhu R., Zhang Y., Zhou X., Yue H., Li Q., Ke J., Wang Y., Miao F., Chen T. (2024). Deleting the C84L Gene from the Virulent African Swine Fever Virus SY18 Does Not Affect Its Replication in Porcine Primary Macrophages but Reduces Its Virulence in Swine. Pathogens.

[236] Hübner A., Keßler C., Pannhorst K., Forth J.H., Kabuuka T., Karger A., Mettenleiter T.C., Fuchs W. (2019). Identification and characterization of the 285L and K145R proteins of African swine fever virus. J. Gen. Virol..

[237] Lu P., Zhou J., Wei S., Takada K., Masutani H., Okuda S., Okamoto K., Suzuki M., Kitamura T., Masujin K. (2023). Comparative genomic and transcriptomic analyses of African swine fever virus strains. Comput. Struct. Biotechnol. J..

[238] Gladue D.P., Gomez-Lucas L., Largo E., Velazquez-Salinas L., Ramirez-Medina E., Torralba J., Queralt M., Alcaraz A., Nieva J.L., Borca M.V. (2023). African swine fever virus gene B117L encodes a small protein endowed with low-pH-dependent membrane Permeabilizing activity. J. Virol..

[239] Fiori M.S., Ferretti L., Floris M., Loi F., Nardo A.D., Sechi A.M., Ladu A., Puggioni G., Sanna D., Scarpa F. (2021). First Genomic evidence of dual African swine fever virus infection: Case report from recent and historical outbreaks in Sardinia. Viruses.

[240] Yang J., Zhu R., Zhang Y., Fan J., Zhou X., Yue H., Li Q., Miao F., Chen T., Mi L. (2023). SY18ΔL60L: A new recombinant live attenuated African swine fever virus with protection against homologous challenge. Front. Microbiol..

