# Supplementary material for: Comprehensive Characterization of the Genetic Landscape of African Swine Fever Virus: Insights into Infection Dynamics, Immunomodulation, Virulence and Genes with Unknown Function
Source: Animals (Basel). 2024 Jul 26;14(15):2187. doi: 10.3390/ani14152187 (PMC11311002; doi:10.3390/ani14152187)
Supplement: Supplementary file 1 [file animals-14-02187-s001.zip › animals-3079750-supplementary/Supplementary table 2.pdf]

Gene	Mechanism	Deletion Mutant	Year	Result		
				Attenuation (No, Moderate, High, Complete)	Protection (No, Partial, High, Complete)	
					Homologous	Heterologous
Mediators of Immunomodulatory Pathways						
A151R	Inhibits of viral replication<br>Negatively regulates cGAS-STING-mediated IFN-β production	ASFV-G-ΔA151R	2022	High	High (ASFV-G)	-
A238L (5EL)	Inhibits NF-κB-p65 and action of calcineurin phosphatase<br>Controls c-Jun and suppresses TNF-α and COX-2	Δ5EL	1997	No	-	-
		E70ΔA238L	2008	No	-	-
		NH/P68ΔA238L	2018	High	Complete (L60)	No (Arm07)
		A238L from Pol18/28298/Out111 (CRISPR/Cas9)	2020	-	-	-
		ASFV-Ke-ΔA238L (CRISPR/Cas9)	2020	-	-	-
		Arm-ΔCD2v-ΔA238L	2022	Complete	-	Complete (Paju)
		ASFV-Ke-ΔA238L	2022	Moderate	-	-
		ASFV-Ke-ΔEP402RΔA238L	2022	High	Partial (ASFV-Ke)	-
DP96R (UK)	Inhibiting cGAS-STING-mediated IFN-I signaling pathway	ΔUK from E70	1998	High	Complete (E70)	-
		OUR T88/3ΔDP2	2013	-	Partial (OUR T88/1)	-
		ASFV-G-Δ9GL/ΔUK	2017	Complete	Complete (ASFV-G)	-
		ASFV-G ΔUK	2019	No	-	-
		HLJ/18-CD2v&UK-del	2020	Moderate	-	-
		HLJ/18-9GL&UK-del	2020	Complete	No	-
		BA71ΔCD2DP96R	2021	Low		No (ASFV-G)

		ASFV-GS-Δ18R/NL/UK	2023	Complete	Complete (ASFV-G)	-
E120R	Dissemination of virions<br>Inhibition of IFN-β promoter activity and NF-κB pathway	FLAG-E120R-Δ72-73aa	2021	-	-	-
I226R	Inhibits cGAS/STING-mediated IFN-β and ISRE reporter activation<br>Suppresses phosphorylation of NF-κB-p65<br>Targets NEMO	SY18ΔI226R	2021	Complete	Complete (SY18)	-
I267L	Interacts with Riplet to suppress RIG-I-mediated IFNB1 expression<br>Hemorrhage regulator	SY18ΔI267L	2021	No	-	-
		ASFVΔI267L	2022	Moderate	-	-
I329L	Modulator of of IFN-I, NF-κB and pro-inflammatory cytokines	OURT88/3ΔI329L	2020	No	No (OURT88/1)	-
		GeorgiaΔI329L	2020	No	-	-
I73R	Suppresses transcription of TNF-α, NF-κB-mediated IFN-β<br>Prevent TNF mRNA and IRF3 from moving to the cytoplasm	ASFV-GZΔI73R	2023	High	Complete (ASFV-GZ)	-
L83L	Suppresses IFN-β and ISRE expression levels through STING degradation using Tollip<br>Binds with IL-1β	ASFV-G-ΔL83L	2018	No	-	-
MGF-505-2R	Inhibits cGAS-STING-mediated IFN-β pathway by interfering with TBK1 phosphorylation	HLJ/18-6GD	2020	Complete	High	-
		HLJ/18-7GD	2020	Complete	High	-
		Arm/07-ΔMGF505-2R-GFP	2024	Complete	partial (Arm/07)	partial (Paju)
MGF-505-3R	Inhibits TBK1, IRF3, and IκBα phosphorylation, lowers ISRE and IFN-β activity<br>Targets TBK1 for degradation	HLJ/18-6GD	2020	Complete	High	-
		HLJ/18-7GD	2020	Complete	High	-
MGF-505-11R	Inhibits IFN-β, ISG15, and ISG56 mRNA, TBK1 and IRF3 phosphorylation, and blocks	-	-	-	-	-

	the activation of cGAS, STING, TBK1, IKKε, IRF7, and IRF3-5D					
MGF-360-9L	Inhibition of the JAK-STAT pathway and IFN cascade	ASFV-Δ360-9L	2022	Moderate	-	-
MGF-360-10L	Targets JAK1 for degradation	ASFV-Δ10L	2023	High	-	-
MGF-360-11L	Inhibits IFN-β, ISG15, and ISG56 mRNA, TBK1 and IRF3 phosphorylation, and blocks the activation of cGAS, STING, TBK1, IKKε, IRF7, and IRF3-5D	-	-	-	-	-
MGF-360-12L	Inhibits TBK1 and IRF3-5D's exogenous expression, ISRE promoter downstream of IFN-β signaling	HLJ/18-6GD	2020	Complete	High	-
		HLJ/18-7GD	2020	Complete	High	-
MGF-360-13L	inhibition of cGAS-STING-mediated IFN-I pathway, ISRE response and IFN-β activation	HLJ/18-6GD	2020	Complete	High	-
		HLJ/18-7GD	2020	Complete	High	-
MGF-360-14L	Inhibition of cGAS-STING pathway and K63-linked ubiquitination of IRF3	HLJ/18-6GD	2020	Complete	High	-
		HLJ/18-7GD	2020	Complete	High	-
A276R (MGF 360-15R)	Suppression of IFN-β through TLR3 and cGAS-STING pathways	NH/P68ΔA276R	2018	No	-	No (Arm07)
MGF300-2R	Degrades IKKα and IKKβ via selective autophagy pathway using Tollip	Δ2R	2023	Moderate	-	-
MGF-110-9L	Function unknown	ASFV-D9L	2021	Moderate	-	-
QP383R	Interferes with NLRP3 and AIM2-mediated inflammation<br>Triggers upregulation of palmitoylation levels of cGAS, affects its DNA binding activity	ASFV-ΔQP383R	2022	Moderate	No (ASFV-G)	-
		ASFV-ΔQP509L/QP383R	2022	Moderate	No (CN/GS/2018)	-
Mediators of Apoptosis and Cell Autophagy						
A179L	Inhibitor of apoptosis, interacts with Bcl-2 proteins and enhances necroptosis	BeninΔA179L	2023	High	No (Benin 97/1)	-
A224L (IAPv)		Ba71VΔIAP	2001	-	-	-

	Inhibitor of apoptosis, binds with caspase-3 and stimulates IKK	NH/P68ΔA224L	2018	High	Complete (L60)	No (Arm07)
DP71L (NL)	Regulates ER stress-mediated apoptosis, recruits PP1 to dephosphorylate eIF2α	E70/43 recombinant	1996	High	Complete (E70)	-
		OUR T88/3ΔDP2	2013	Moderate	-	-
		ASFV-G-ΔNL	2019	Moderate	-	-
		ASFV-G-Δ9GL/ΔNL/ΔUK	2019	Complete	No (ASFV-G)	-
		ASFV-GS-Δ18R/NL/UK	2023	High	Complete (ASFV-G)	-
EP153R	Hemadsorption, inhibition of apoptosis through caspase 3 suppression	ΔEP153R	2004	-	-	-
		NH/P68ΔEP153R	2018	High	Complete (L60)	No (Arm07)
		ASFV-G-Δ9GL/ΔCD2v/ΔEP153R	2020	High	No (ASFV-G)	-
		BA71ΔCD2EP153R	2021	Low	-	No (ASFV-G)
		BeninΔDP148RΔEP153R	2022	No	-	-
		BeninΔDP148RΔEP153RΔEP402R	2022	Complete	Partial (Benin 97/1)	-
E199L	Enhances complete cell autophagy Triggers cell apoptosis through the mitochondrial apoptotic pathway	-	-	-	-	-
K205R	Aid in ER stress-triggered autophagy Phosphorylates NF-κB subunits	-	-	-	-	-
Mediators of Infection Cycle and Other Functions						
A240L (TK)	Involved in synthesis of deoxynucleoside triphosphates	ASFV v5.3	1998	-	Partial (Malawi LiL-20/1V)	-
		ASFV vH53	1998	-	-	-
		ASFV-G/P-ΔTK	2016	Complete	No (ASFV-G)	-
B119L	Thiol-disulfide exchange reactions and virion maturation	Δ9GL mutant	2000	High	Complete (Malawi Lil-20/1)	-
		Pr4Δ9GL mutant	2004	High	Complete	-

		ASFV-G-Δ9GL	2015	High	Complete (ASFV-G)	-
		ASFV-G-Δ9GL/ΔUK	2017	Complete	Complete (ASFV-G)	-
		9GL1Δ and 9GL2Δ	2020	-	-	-
		HLJ/18-9GL&UK-del	2020	Complete	No (HLJ/18)	-
DP148R (MGF 360-18R)	-	BeninΔDP148R	2017	High	Complete (Benin 97/1)	
		HLJ/18-DP148R-del	2020	No	-	-
		GeorgiaΔDP148R	2021	No	-	-
		ASFV-GS-Δ18R/NL/UK	2023	High	Complete (ASFV-G)	
H108R	Encodes transmembrane protein	ASFV-G-ΔH108R	2022	High	Complete (ASFV-G)	-
O61R	Aids viral adsorption through cellular receptor recognition	-	-	-	-	-
Multifunctional Genes						
A528R (MGF505-7R)	Leads STING and JAK1-JAK2 into degradation<br>Inhibits ISRE. Also suppresses phosphorylation and translocation of p65<br>Binds to NLRP3 to inhibit inflammasome complex	ASFV-Δ7R	2021	Moderate	-	-
		ASFV-Δ110-9L/505-7R	2022	High	Complete (CN/GS/2018)	-
EP402R	Involved in Hemadsorption<br>Inhibits IFN-I production by interacting with STING's transmembrane domain and hindering its translocation to the Golgi apparatus<br>Interacts with SH3P7 and AP-1	BA71ΔCD2	2017	High	Complete (BA71)	High (E75,ASF-G)
		ASFV-ΔEP402R	2023	-	Partial (HLJ/18)	-
		ASFV-G-Δ8DR	2020	High	No (ASF-G)	-
		ASFV-G-Δ9GL/ΔCD2v and ASFV-G-Δ9GL/ΔCD2v/ΔEP153R	2020	High	No (ASF-G)	-

		ASFV-Ke-ΔEP402R	2022	Moderate	High (ASFV-Kenya-1033)	-
		ASFV-Ke-ΔEP402RΔA238L	2022	Moderate	Partial (ASFV-Kenya-1033)	-
		ΔCongoCD2v	2021	-	No (Congo-v)	-
		ΔCongo-v_CD2v	2023	-	No (Congo-v)	-
		BeninΔDP148RΔEP402R	2022	Moderate	Complete (Benin 97/1)	-
		BeninΔDP148RΔEP153RΔEP402R	2022	Complete	No (Benin 97/1)	-
		ASFV-ΔECM3	2022	Complete	No (GZ201801)	-
S273R	Inhibition of cGAS-STING-mediated IFN-β production, IKKε de-sumoylation<br>Degrades FoxJ1<br>Interrupts IRF3 phosphorylation and JAK-STAT pathway<br>Regulates pyroptosis<br>Targets cytoplasmic stress granules	-	-	-	-	-
